# Rhein–Amino Acid Ester Conjugates as Potential Antifungal Agents: Synthesis and Biological Evaluation

**DOI:** 10.3390/molecules28052074

**Published:** 2023-02-22

**Authors:** Shunshun Chen, Meimei Wang, Linhua Yu, Jinchao Shi, Yong Zhang, Yao Tian, Li Li, Xiang Zhu, Junkai Li

**Affiliations:** 1 Institute of Pesticides, College of Agriculture, Yangtze University, Jingzhou 434025, China; 2 Hubei Engineering Technology Center for Pest Forewarning and Management, College of Agriculture, Yangtze University, Jingzhou 434025, China; 3 State Key Laboratory Breeding Base of Green Pesticide and Agricultural Bioengineering, Key Laboratory of Green Pesticide and Agricultural Bioengineering, Ministry of Education, Guizhou University, Guiyang 550025, China

**Keywords:** rhein, amino acid ester, conjugate, antifungal activity

## Abstract

In the search for crop protectants, amino acid ester conjugates have been widely investigated as potential antifungal agents. In this study, a series of rhein–amino acid ester conjugates were designed and synthesized in good yields, and their structures were confirmed by ^1^H-NMR, ^13^C-NMR and HRMS. The bioassay results revealed that most of the conjugates exhibited potent inhibitory activity against *R. solani* and *S. sclerotiorum*. In particular, conjugate **3c** had the highest antifungal activity against *R. solani* with an EC_50_ value of 0.125 mM. For *S. sclerotiorum*, conjugate **3m** showed the highest antifungal activity with an EC_50_ value of 0.114 mM. Satisfactorily, conjugate **3c** exhibited better protective effects than that of the positive control, physcion, against powdery mildew in wheat. This research supports the role of rhein–amino acid ester conjugates as potential antifungal agents for plant fungal diseases.

## 1. Introduction

Fungicides play an irreplaceable role in protecting crops from pathogenic fungal infections and ensuring crop yield and quality [1,2]. Due to the long-term use of traditional fungicides, the emergence of drug-resistant strains and problems caused by the residue of these fungicides in the environment have become increasingly prominent [3,4]. Therefore, new antifungal agents with high efficiency, low toxicity and low environmental pollution must urgently be developed to address these challenges.

**Figure 1:**
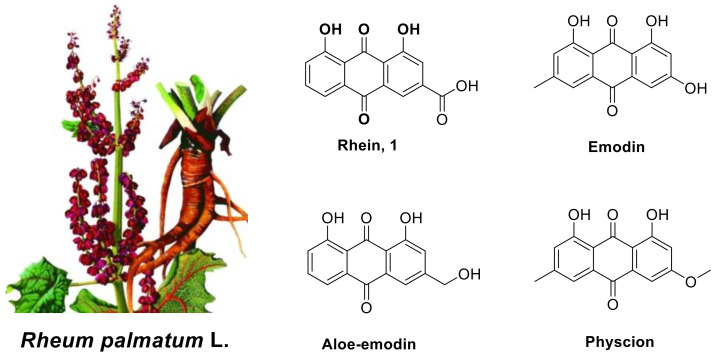
The *Rheum palmatum* L. and the structures of rhein (**1**) and its active analogues emodin, aloe-emodin and physcion.

Natural products (NPs) have high chemical structure diversity, a wide range of biological activities and can easily be degraded by the environment, meaning that they play an important role in the development of crop protection agents [5,6]. The tuber of *Rheum palmatum* L. (Figure 1) is rich in 1,8-dihydroxy anthraquinones, and it is often used as a traditional folk medicine to treat rheumatoid arthritis, inflammation, cancer and cardiovascular diseases [7,8,9,10]. The active 1,8-dihydroxy anthraquinones in the tuber of *Rheum palmatum* L. are mainly rhein (1) and its analogs, which include emodin, aloeemodin, physcion (Figure 1) and so on [11,12,13]. This research mainly focused on the modification of the chemical structure and pharmacological activities of rhein. Our previous studies have shown that various simple derivatives of rhein exhibit certain insecticidal and antifungal activities, especially against some plant pathogens [14]. These findings suggest that rhein could be used as a lead structure for the discovery of new antifungal agents.

Amino acids are a class of active small molecules in organisms with important physiological and structural functions [15,16]. In addition, a significant number of amino acid or amino acid ester conjugates have been found in plants, in which a carboxylic group is usually conjugated with biologically active molecules via the amide bonds, such as hormones, flavonoids, vitamins, steroids and several heterocyclic compounds [17,18,19,20]. For this reason, many researchers have tried to conjugate active lead structures with amino acid (esters) moieties to find structures with better pharmacological or antifungal activities.

Herein, in order to find higher antifungal rhein derivatives, a series of rhein–amino acid ester conjugates were designed and synthesized by conjugating rhein with L- or D-amino acid esters via an amide bond. The antifungal activities of all the synthesized compounds were tested in vitro and in vivo, and the primary structure–activity relationship was discussed.

## 2. Results and Discussion

### 2.1. Chemistry

**Figure 2:**
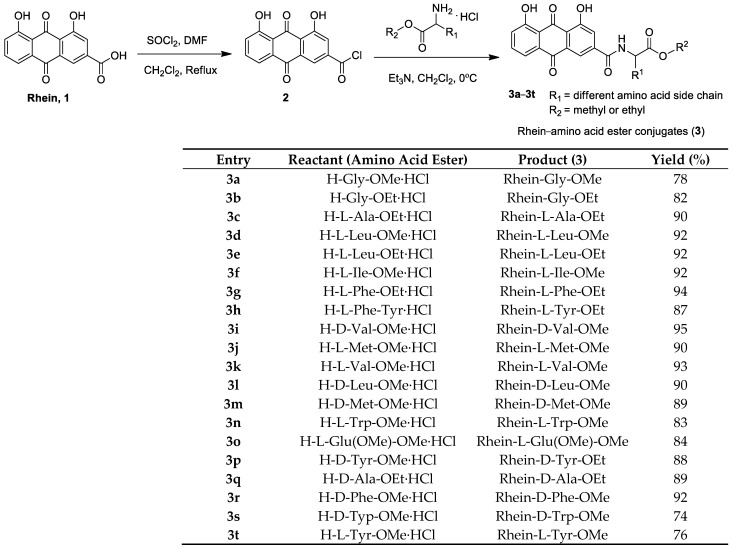
Preparation of rhein–amino acid ester conjugates **3a–3t**.

The synthetic route of rhein–amino acid ester conjugates **3a**–**3t** is shown in Figure 2. Rhein was treated with SOCl_2_ at the reflux temperature in CH_2_Cl_2_ solution for 8 h, and intermediate **2** was afforded after the evaporation of the solvent. Then, intermediate **2** was allowed to react with the corresponding L- or D-amino acid esters in CH_2_Cl_2_ at 0 °C for 2 h to yield target compounds **3a**–**3t**. The corresponding reactant (amino acid ester) was used in the synthesis of rhein–amino acid ester conjugates, and the yields are listed in Figure 2, indicating that the rhein–amino acid ester conjugates were obtained in good yields and varied from 74% to 94%.

The structures of all the synthesized rhein–amino acid ester conjugates **3a**–**3t** were effectively characterized by ^1^H NMR, ^13^C NMR and HRMS analyses (the corresponding spectra are available in the Supplementary Materials).

### 2.2. In Vitro Antifungal Activity

**Figure 3:**
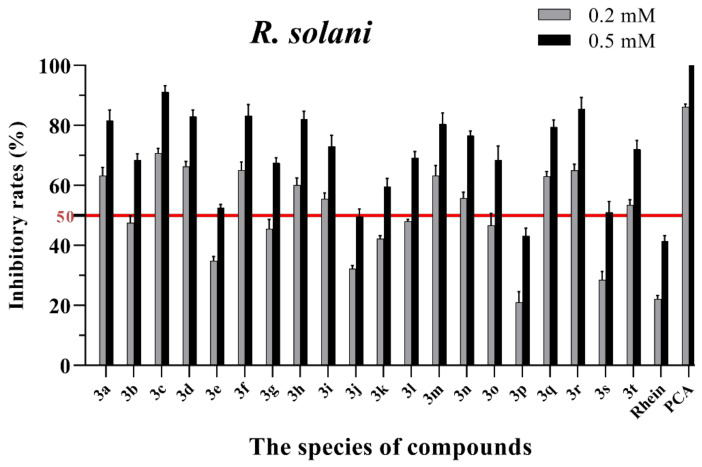
Mycelial growth % inhibition of chemicals against *R. solani*. The bold red line is the identity line with the inhibition rate of 50%.

**Figure 4:**
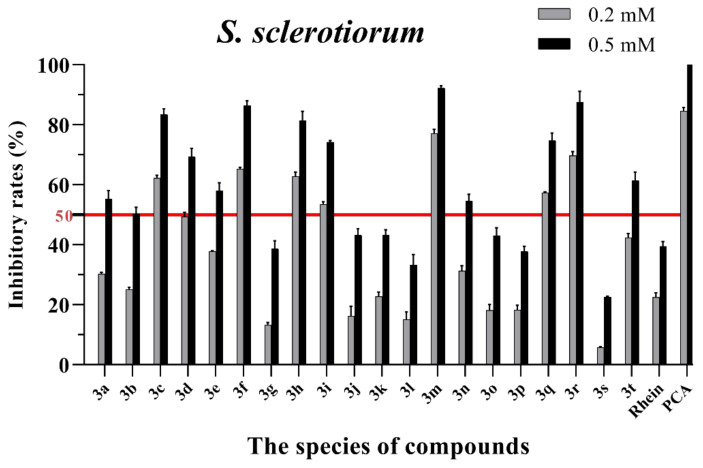
Mycelial growth % inhibition of chemicals against *S. sclerotiorum*. The bold red line is the identity line with the inhibition rate of 50%.

**Figure 5:**
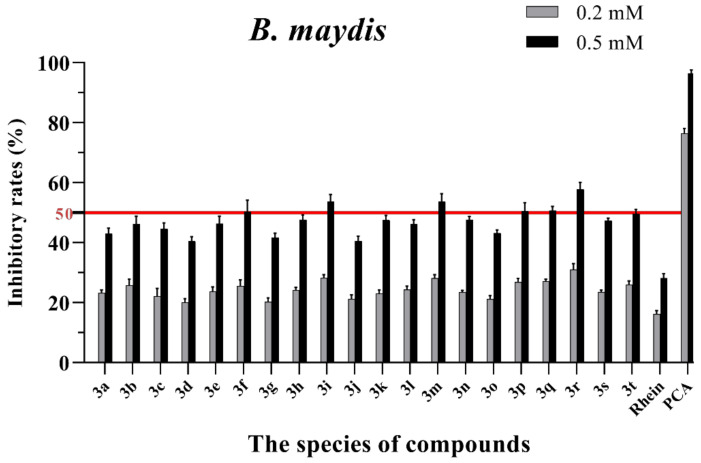
Mycelial growth % inhibition of chemicals against *B. maydis*. The bold red line is the identity line with the inhibition rate of 50%.

**Figure 6:**
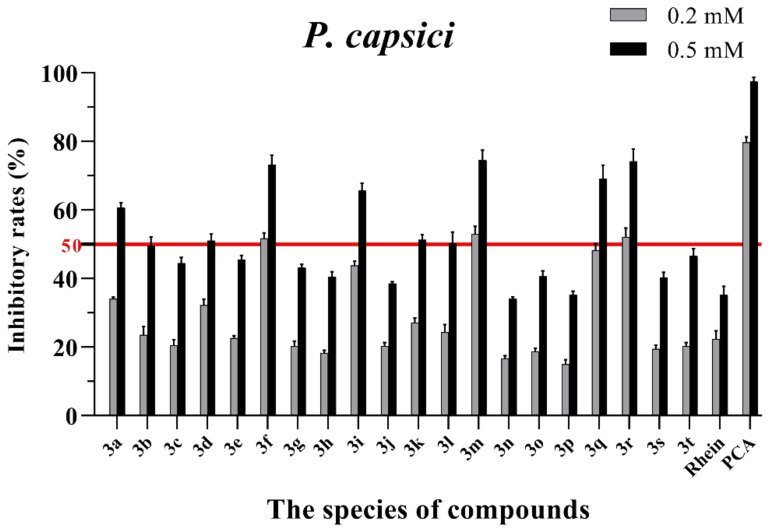
Mycelial growth % inhibition of chemicals against *P. capsici*. The bold red line is the identity line with the inhibition rate of 50%.

The in vitro antifungal activities of these rhein–amino acid ester conjugates **3a–3t** were initially screened at 0.2 mM and 0.5 mM against four phytopathogenic fungi (*Rhizoctonia solani*, *Sclerotinia sclerotiorum*, *Bipolaris maydis* and *Phytophthora capsici*) using the mycelial growth rate method. The natural lead compound, rhein, was used as reference control. A commercial biofungicide, phenazine-1-carboxylic acid (PCA), was used as the positive control. The preliminary bioassay results of these conjugates against four phytopathogenic fungi are summarized in Table S1 and Figure 3, Figure 4, Figure 5 and Figure 6, respectively.

As seen in Figure 3, all of the conjugates exhibited moderate-to-good antifungal activities against *R. solani* at the concentration of 0.2 mM (this concentration of rhein was equal to 56.8 mg/L). Among all 20 conjugates, 11 conjugates, **3a**, **3c**, **3d**, **3f**, **3h**, **3i**, **3m**, **3n**, **3q**, **3r** and **3t**, showed an efficacy over 50% against *R. solani* at 0.2 mM. In particular, at 0.5 mM (this concentration of rhein was equal to 142.1 mg/L), most of the conjugates showed great antifungal activities against *R. solani*, and compound **3c** (rhein-L-Ala-OEt) exhibited excellent antifungal activities against *R. solani* with an inhibition rate of more than 90%. The data in Figure 4 indicated that seven conjugates (**3c**, **3f**, **3h**, **3i**, **3m**, **3q** and **3r**) out of the twenty tested conjugates showed moderate-to-strong antifungal activities against *S. sclerotiorum* with an efficacy over 50% at 0.2 mM. In particular, compound **3m** (rhein-D-Met-OMe) exhibited excellent antifungal activities against *S. sclerotiorum* with an inhibition rate of more than 90% at 0.5 mM. As seen in Figure 5 and Figure 6, almost all the conjugates presented poor antifungal activities against *B. maydis* and *P. capsici*, among which only compounds **3f**, **3m** and **3r** showed an efficacy over 50% against *P. capsici* at 0.2 mM. Through this analysis of the preliminary antifungal activities results, it was inferred that these rhein–amino acid ester conjugates can be used as antifungal lead structures against *R. solani* and *S. sclerotiorum*.

In order to understand the antifungal activity of the more active conjugates against *R. solani* and *S. sclerotiorum* more clearly, the conjugates with inhibition rates of >50% at a concentration of 0.2 mM against these two fungi (shown in Figure 3 and Figure 4) were further assayed to determine their median effective concentrations (EC_50_). The results are presented in Table 1. The results showed that 11 conjugates (**3a**, **3c**, **3d**, **3f**, **3h**, **3i**, **3m**, **3n**, **3q**, **3r** and **3t**) exhibited potent antifungal activity against *R. solani* with EC_50_ values between 0.125 and 0.197 mM, but these values were lower than that of PCA (EC_50_ = 0.083 mM). Additionally, conjugate **3c** had the highest antifungal activity with an EC_50_ value of 0.125 mM. For *S. sclerotiorum*, conjugate **3m** (rhein-D-Met-OMe) showed the highest antifungal activity with an EC_50_ value of 0.114 mM, but this value was lower than PCA (EC_50_ = 0.088 mM).

### 2.3. In Vivo Antifungal Activity against Powdery Mildew in Wheat

The antifungal activity of conjugate **3c** against powdery mildew in wheat was evaluated at 0.4 mM and 0.2 mM. As shown in Table 2, in terms of its curative activity, conjugate **3c** exhibited potent antifungal activity against powdery mildew in wheat with control efficiencies of 84.4% and 61.2% at 0.4 mM and 0.2 mM, respectively, which were similar values to those of the control agent, physcion (87.4% at 0.4 mM, and 67.2% at 0.2 mM). In terms of its protective activity, conjugate **3c** had antifungal activity of 66.7% and 53.8% against powdery mildew in wheat at 0.4 mM and 0.2 mM, respectively, which were higher values than those of the control agent, physcion (54.2% at 0.4 mM, and 38.4% at 0.2 mM).

Presently, extensive studies regarding the biological activities of amino acid ester conjugates are being carried out based on the conjugation of amino acid esters with an active lead structure. Zhang designed and synthesized a series of novel amino acid ester-coupled caffeoylquinic acid derivatives, and the biological evaluation suggested that some amino acid ester-coupled derivatives exhibited varying degrees of lipid-lowering effects on oleic acid-elicited lipid accumulation in HepG2 liver cells [18]. Studies have shown that some curcumin–amino acid conjugates exhibit enhanced anti-inflammatory properties with potency higher than that of standard NSAID references (non-steroidal anti-inflammatory drugs, indomethacin, and ibuprofen) [21]. In our previous research, to improve the bioactivities of PCA (a biofungicide), a series of PCA–amino acid ester conjugates have been successfully prepared, some of which were shown to be more effective against *Rhizoctonia solani* Kuhn than PCA [22]. In this study, using natural rhein as a lead structure, 20 rhein–amino acid ester conjugates were successfully prepared, and the antifungal activities of these conjugates were initially determined. The current results confirmed that some rhein–amino acid ester conjugates have potent antifungal activities against *R. solani* and *S. sclerotiorum*, and they can be used as lead structures for the development of antifungal agents.

## 3. Materials and Methods

### 3.1. Chemicals and Instruments

All chemicals and reagents were commercially purchased and used directly without further purification. Flash column chromatography and analytical thin layer chromatography (TLC) were performed using silica gel 60 (200–300 mesh) and silica gel aluminum sheets F254 (Qingdao Marine Chemical Ltd., Qingdao, China), respectively. The melting points of all the compounds were determined using a WRR melting point apparatus (Shanghai Jingke Industrial Co. Ltd., Shanghai, China) and were uncorrected. Then, ^1^H NMR and ^13^C NMR spectra were recorded in CDCl_3_ or DMSO-*d_6_* solution at 400 MHz for ^1^H and 101 MHz for ^13^C on a Bruker Avance III HD 500 MHz NMR Spectrometer (Bruker (Beijing) Scientific Technology Co. Ltd., Beijing, China) using tetramethylsilane (TMS) as an internal standard. High-resolution mass spectra were obtained using a Bruker APEX IV Fourier-transform mass spectrometer (Bruker (Beijing) Scientific Technology Co. Ltd., Beijing, China).

### 3.2. Test Fungus

Plant pathogenic fungi, including *Rhizoctonia solani*, *Sclerotinia sclerotiorum*, *Bipolaris maydis*, and *Phytophthora capsici*, were provided by the Institute of Pesticide Research, Yangtze University, China. These four fungi were grown on potato dextrose agar (PDA) plates at 25 °C and maintained at 4 °C with periodic subculturing. The susceptible wheat variety, Chanceller and the tested pathogenic fungi, *Blumeria graminis*, isolated from a wheat plant infected with powdery mildew, were provided by the Institute for Plant Protection and Soil Sciences, Hubei Academy of Agricultural Sciences.

### 3.3. Preparation of 4,5-Dihydroxy-9,10-dioxo-9,10-dihydroanthracene-2-carbonyl Chloride (***2***)

According to our previous literature report [23], rhein **1** (10 mmol), 30 mL of anhydrous dichloromethane, and 2~3 drops of DMF as a catalyst were added into a single-port bottle for stirring. Then, thionyl chloride (15 mmol) was slowly added to the flask and heated to reflux until the solid disappeared completely. The reflux reaction continued for 8 h, and the solvent was removed on a rotary evaporator to obtain **2** for the next step.

### 3.4. General Procedure for Preparation of Rhein–Amino Acid Ester Conjugates (***3a***–***3t***)

4,5-Dihydroxy-9,10-dioxo-9,10-dihydroanthracene-2-carbonyl chloride **2** (10 mmol) dissolved in 10 mL of anhydrous CH_2_Cl_2_ was added dropwise to a solution of glycine methyl ester hydrochloride (10 mmol), and triethylamine (22 mmol) was used as the attaching acid agent in 50 mL of CH_2_Cl_2_ at 0 °C. The mixture was refluxed for 2 h until the reaction was complete. Then, the solvent was evaporated under vacuum, and the crude product was purified via column chromatography to obtain a pure rhein–glycine methyl ester conjugate (**3a**). Conjugates **3b**–**3t** were also obtained in the same way.

#### 3.4.1. Rhein-Gly-OMe (**3a**): Methyl 2-(4,5-Dihydroxy-9,10-dioxo-9,10-dihydroanthracene-2-carboxamido)acetate

Yellow solid; yield: 78%; m.p, 219~222 °C; ^1^H NMR (400 MHz, CDCl_3_) δ 12.05 (s, 1H, anthraquinone-OH), 11.98 (s, 1H, anthraquinone-H), 8.16 (s, 1H, anthraquinone-H), 7.88 (d, *J* = 7.2 Hz, 1H, anthraquinone-H), 7.79 (s, 1H, anthraquinone-H), 7.74 (t, *J* = 7.9 Hz, 1H, anthraquinone-H), 7.35 (d, *J* = 8.4 Hz, 1H, anthraquinone-H), 6.82 (s, 1H, CONH), 4.29 (d, *J* = 4.8 Hz, 2H, acylamino-CH_2_), 3.84 (s, 3H, CH_3_). ^13^C-NMR (101 MHz, CDCl_3_) δ 209.29, 192.69, 164.30, 162.84, 162.72, 161.46, 137.72, 137.37, 133.49, 133.48, 125.04, 123.45, 120.46, 117.42, 111.67, 96.50, 52.70, 41.91. HRMS calcd for C_18_H_13_NO_7_ [M + H]^+^: 356.0765, found 356.0765.

#### 3.4.2. Rhein-Gly-OMe (**3b**): Ethyl 2-(4,5-Dihydroxy-9,10-dioxo-9,10-dihydroanthracene-2-carboxamido)acetate

Yellow solid; yield: 82%; m.p, 211~214 °C; ^1^H NMR (400 MHz, CDCl_3_) δ 12.05 (s, 1H, anthraquinone-OH), 11.98 (s, 1H, anthraquinone-OH), 8.15 (d, *J* = 1.6 Hz, 1H, anthraquinone-H), 7.87 (dd, *J =* 7.6, *J* = 1.2 Hz, 1H, anthraquinone-H), 7.78 (d, *J =* 1.6 Hz, 1H, anthraquinone-H), 7.76–7.70 (m, 1H, anthraquinone-H), 7.34 (dd, *J =* 8.4, *J* = 1.2 Hz, 1H, anthraquinone-H), 6.85 (s, 1H, CONH), 4.33–4.28 (m, 2H, C*H*_2_CH_3_), 4.27 (d, *J =* 4.8 Hz, 2H, acylamino-CH_2_), 1.34 (t, *J* = 7.2 Hz, 3H, CH_3_). ^13^C-NMR (101 MHz, CDCl_3_) δ 192.69, 181.01, 169.54, 164.91, 162.82, 162.70, 141.55, 137.71, 134.11, 133.44, 125.01, 123.42, 120.44, 117.63, 117.44, 115.80, 61.95, 42.06, 14.18.

HRMS calcd for C_19_H_15_NO_7_ [M + H]^+^: 370.0921, found 370.0923.

#### 3.4.3. Rhein-L-Ala-OEt (**3c**): (R)-Ethyl 2-(4,5-Dihydroxy-9,10-dioxo-9,10-dihydroanthracene-2-carboxamido)propanoate

Yellow solid; yield: 90%; m.p, 223~225 °C; ^1^H NMR (400 MHz, CDCl_3_) δ 12.04 (s, 1H, anthraquinone-OH), 11.98 (s, 1H, anthraquinone-OH), 8.14 (d, *J* = 1.6 Hz, 1H, anthraquinone-H), 7.88 (dd, *J* = 7.6, *J* =1.2 Hz, 1H, anthraquinone-H), 7.77 (d, *J* = 1.6 Hz, 1H, anthraquinone-H), 7.76–7.70 (m, 1H, anthraquinone-H), 7.34 (dd, *J* = 8.4, J =1.2 Hz, 1H, anthraquinone-H), 6.92 (d, *J* = 7.2 Hz, 1H, CONH), 4.79 (t, *J* = 7.2 Hz, 1H, CH), 4.28 (q, *J* = 7.2 Hz, 2H, C*H*_2_CH_3_), 1.58 (d, *J* = 4.8, 3H, CHC*H*_3_), 1.36–1.30 (t, *J* = 7.2 Hz, 3H, CH_2_C*H*_3_). ^13^C-NMR (101 MHz, CDCl_3_) δ 192.70, 181.08, 172.73, 164.30, 162.82, 162.71, 141.87, 137.69, 134.08, 133.46, 125.01, 123.42, 120.44, 117.56, 117.44, 115.82, 61.90, 48.91, 18.53, 14.17. HRMS calcd for C_20_H_17_NO_7_ [M + H]^+^: 384.1078, found 384.1080.

#### 3.4.4. Rhein-L-Leu-OMe (**3d**): (R)-Methyl 2-(4,5-Dihydroxy-9,10-dioxo-9,10-dihydroanthracene-2-carboxamido)-4-methylpentanoate

Yellow solid; yield: 92%; m.p, 177~179 °C; ^1^H NMR (400 MHz, CDCl_3_) δ 12.05 (s, 1H, anthraquinone-OH), 11.98 (s, 1H, anthraquinone-OH), 8.13 (d, *J* = 1.6 Hz, 1H, anthraquinone-H), 7.88 (dd, *J* = 7.6, *J* = 0.8 Hz, 1H, anthraquinone-H), 7.77 (d, *J* = 1.6 Hz, 1H, anthraquinone-H), 7.76–7.71 (m, 1H, anthraquinone-H), 7.35 (dd, *J* = 8.4, *J* = 1.2 Hz, 1H, anthraquinone-H), 6.70 (d, *J* = 8.0 Hz, 1H, CONH), 4.88 (dd, *J* = 11.2, *J* = 5.6 Hz, 1H, acylamino-CH), 3.80 (s, 3H, CH_3_), 1.81–1.70 (m, 3H, C*H*_2_C*H*(CH_3_)_2_), 1.04–0.97 (m, 6H, CH(C*H*_3_)_2_). ^13^C-NMR (101 MHz, CDCl_3_) δ 192.68, 181.11, 173.18, 164.61, 162.83, 162.71, 141.76, 137.71, 134.08, 133.44, 125.04, 123.50, 120.45, 117.59, 117.38, 115.82, 52.59, 51.47, 41.76, 25.03, 22.82, 22.02. HRMS calcd for C_22_H_21_NO_7_ [M + H]^+^: 412.1391, found 412.1393.

#### 3.4.5. Rhein-L-Leu-OEt (**3e**): (R)-Ethyl 2-(4,5-Dihydroxy-9,10-dioxo-9,10-dihydroanthracene-2-carboxamido)-4-methylpentanoate

Yellow solid; yield: 92%; m.p, 145~147 °C; ^1^H NMR (400 MHz, CDCl_3_) δ 12.02 (s, 1H, anthraquinone-OH), 11.97 (s, 1H, anthraquinone-OH), 8.11 (d, *J* = 1.6 Hz, 1H, anthraquinone-H), 7.86 (dd, *J* = 7.6, *J* =1.2 Hz, 1H, anthraquinone-H), 7.76–7.68 (m, 2H, anthraquinone-H), 7.33 (dd, *J* = 8.4, *J* = 1.2 Hz, 1H, anthraquinone-H), 6.82 (d, *J* = 8.0 Hz, 1H, CONH), 4.85 (m, 1H, acylamino-CH), 4.26 (q, *J* = 7.2 Hz, 2H, C*H*_2_CH_3_), 1.83–1.67 (m, 3H, C*H*_2_C*H*(CH_3_)_2_), 1.33 (t, *J* = 7.2 Hz, 3H, CH_2_*CH*_3_), 1.01 (dd, *J* = 6.0, *J =* 4.4 Hz, 6H, CH(C*H*_3_)_2_). ^13^C-NMR (101 MHz, CDCl_3_) δ 192.63, 181.04, 172.85, 164.61, 162.79, 162.65, 141.83, 137.68, 134.00, 133.42, 125.00, 123.43, 120.42, 117.50, 117.44, 115.79, 61.73, 51.56, 41.74, 25.04, 22.86, 22.03, 14.19. HRMS calcd for C_23_H_23_NO_7_ [M + H]^+^: 426.1547, found 426.1552.

#### 3.4.6. Rhein-L-Ile-OMe (**3f**): (2R,3R)-Methyl 2-(4,5-Dihydroxy-9,10-dioxo-9,10-dihydroanthracene-2-carboxamido)-3-methylpentanoate

Yellow solid; yield: 92%; m.p, 162~164 °C; ^1^H NMR (400 MHz, CDCl_3_) δ 12.06 (s, 1H, anthraquinone-OH), 11.98 (s, 1H, anthraquinone-OH), 8.14 (d, *J* = 1.6 Hz, 1H, anthraquinone-H), 7.89 (dd, *J* = 7.6, *J* = 1.2 Hz, 1H, anthraquinone-H), 7.74 (dd, *J* = 12.0, *J* = 4.8 Hz, 2H, anthraquinone-H), 7.35 (dd, *J* = 8.4, *J* = 1.2 Hz, 1H, anthraquinone-H), 6.79 (d, *J* = 8.0 Hz, 1H, CONH), 4.83 (dd, *J* = 8.4, *J* = 4.8 Hz, 1H, acylamino-CH), 3.80 (s, 3H, CH_3_), 2.11–2.01 (m, 1H, acylamino-CHC*H*, 1.26 (s, 2H, CH_2_), 1.05–0.95 (m, 6H, C*H*_3_CH(CH_2_C*H*_3_)). ^13^C-NMR (101 MHz, CDCl_3_) δ 192.70, 181.08, 172.14, 165.93, 164.67, 162.83, 162.70, 142.00, 137.72, 134.12, 133.46, 125.03, 123.36, 120.45, 117.47, 99.99, 57.15, 52.40, 38.24, 25.42, 15.53, 11.61. HRMS calcd for C_22_H_21_NO_7_ [M + H]^+^: 412.1391, found 412.1397.

#### 3.4.7. Rhein-L-Phe-OEt (**3g**): (R)-Ethyl 2-(4,5-Dihydroxy-9,10-dioxo-9,10-dihydroanthracene-2-carboxamido)-3-phenylpropanoate

Yellow solid; yield: 94%; m.p, 178~180 °C; ^1^H NMR (400 MHz, CDCl_3_) δ 12.04 (s, 1H, anthraquinone-OH), 11.98 (s, 1H, anthraquinone-OH), 8.06 (d, *J* = 1.6 Hz, 1H, anthraquinone-H), 7.87 (dd, *J* = 7.6, *J* = 1.2 Hz, 1H, anthraquinone-H), 7.76–7.69 (m, 1H, anthraquinone-H), 7.66 (d, *J* = 1.6 Hz, 1H, Ar-H), 7.37–7.26 (m, 4H, Ar-H), 7.20–7.14 (m, 2H, anthraquinone-H), 6.74 (d, *J* = 7.6 Hz, 1H, CONH), 5.10–5.01 (m, 1H, acylamino-CH), 4.25 (q, *J* = 7.2 Hz, 2H, C*H*_2_CH_3_), 3.36–3.25 (m, 2H, acylamino-CHC*H*_2_), 1.30 (t, *J* = 7.2 Hz, 3H, CH_3_). ^13^C-NMR (101 MHz, CDCl_3_) δ 192.68, 180.96, 171.19, 164.43, 162.80, 162.66, 141.88, 137.70, 135.56, 134.11, 133.45, 129.35, 128.74, 127.40, 124.98, 123.18, 120.42, 117.58, 117.52, 115.79, 61.92, 53.84, 37.90, 14.16. HRMS calcd for C_26_H_21_NO_7_ [M + H]^+^: 460.1391, found 460.1398.

#### 3.4.8. Rhein-L-Tyr-OEt (**3h**): (R)-Ethyl 2-(4,5-Dihydroxy-9,10-dioxo-9,10-dihydroanthracene-2-carboxamido)-3-(4-hydroxyphenyl)propanoate

Yellow solid; yield: 87%; m.p, 192~194 °C; ^1^H NMR (400 MHz, CDCl_3_) δ 12.04 (s, 1H, anthraquinone-OH), 11.98 (s, 1H, anthraquinone-OH), 8.07 (s, 1H, anthraquinone-H), 7.87 (d, *J* = 7.6 Hz, 1H, anthraquinone-H), 7.76–7.71 (m, 1H, anthraquinone-H), 7.68 (d, *J* = 1.6 Hz, 1H, anthraquinone-H), 7.34 (d, *J* = 8.4 Hz, 1H, anthraquinone-H), 7.03 (d, *J* = 8.4 Hz, 2H, Ar-H), 6.79 (d, *J* = 8.4 Hz, 2H, Ar-H), 6.70 (d, *J* = 7.6 Hz, 1H, CONH), 5.03 (d, *J* = 7.6 Hz, 1H, acylamino-CH), 4.25 (d, *J* = 7.2 Hz, 2H, acylamino-CHC*H*_2_), 3.22 (dd, *J* = 14.8, *J* = 5.6 Hz, 2H, C*H*_2_CH_3_), 1.31 (t, *J* = 7.2 Hz, 3H, CH_3_). ^13^C-NMR (101 MHz, CDCl_3_) δ 192.68, 180.96, 171.19, 164.43, 162.80, 162.66, 141.88, 137.70, 135.56, 134.11, 133.45, 129.35, 128.74, 127.40, 124.98, 124.84, 123.18, 120.42, 117.52, 115.79, 61.92, 53.84, 37.90, 14.16. HRMS calcd for C_26_H_21_NO_8_ [M + H]^+^: 476.134, found 476.1348.

#### 3.4.9. Rhein-D-Val-OMe (**3i**): (S)-Methyl 2-(4,5-Dihydroxy-9,10-dioxo-9,10-dihydroanthracene-2-carboxamido)-3-methylbutanoate 

Yellow solid; yield: 95%; m.p, 177~180 °C; ^1^H NMR (400 MHz, CDCl_3_) δ 12.05 (s, 1H, anthraquinone-OH), 11.97 (s, 1H, anthraquinone-OH), 8.14 (d, *J* = 1.6 Hz, 1H, anthraquinone-H), 7.88 (dd, *J* = 7.5, *J* =1.2 Hz, 1H, anthraquinone-H), 7.74 (dd, *J* = 11.6, *J* = 4.8 Hz, 2H, anthraquinone-H), 7.34 (dd, *J* = 8.4, *J* = 1.2 Hz, 1H, anthraquinone-H), 6.80 (d, *J* = 8.4 Hz, 1H, CONH), 4.80 (dd, *J* = 8.4, *J* = 4.8 Hz, 1H, acylamino-CH), 3.81 (s, 3H, CH_3_), 2.39–2.23 (m, 1H, acylamino-CHC*H*), 1.04 (dd, *J* = 6.8, *J* = 5.6 Hz, 6H, CH(C*H*_3_)_2_). ^13^C-NMR (101 MHz, CDCl_3_) δ 192.67, 181.05, 172.20, 164.87, 162.81, 162.68, 142.01, 137.72, 134.10, 133.44, 125.02, 123.36, 120.44, 117.57, 117.49, 115.80, 57.83, 52.46, 31.64, 19.02, 18.07. HRMS calcd for C_21_H_19_NO_7_ [M + H]^+^: 398.1234, found 398.1242.

#### 3.4.10. Rhein-L-Met-OMe (**3j**): (R)-Methyl 2-(4,5-Dihydroxy-9,10-dioxo-9,10-dihydroanthracene-2-carboxamido)-4-(methylthio)butanoate

Yellow solid; yield: 90%; m.p, 184~186 °C; ^1^H NMR (400 MHz, CDCl_3_) δ 12.04 (s, 1H, anthraquinone-OH), 11.97 (s, 1H, anthraquinone-OH), 8.16 (d, *J* = 1.6 Hz, 1H, anthraquinone-H), 7.90–7.84 (m, 1H, anthraquinone-H), 7.79–7.70 (m, 2H, anthraquinone-H), 7.37–7.31 (m, 1H, anthraquinone-H), 7.18 (d, *J* = 7.6 Hz, 1H, CONH), 5.02–4.94 (m, 1H, acylamino-CH), 3.83 (s, 3H, CH_3_), 2.63 (t, *J* = 7.2 Hz, 2H, C*H*_2_SCH_3_), 2.38–2.16 (m, 2H, acylamino-CHC*H*), 2.14 (d, *J* = 6.8 Hz, 3H, SCH_3_). ^13^C-NMR (101 MHz, CDCl_3_) δ 192.67, 181.04, 172.08, 164.63, 162.82, 162.69, 141.55, 137.74, 134.09, 133.42, 125.04, 123.45, 120.46, 117.63, 117.47, 115.79, 52.87, 52.44, 31.25, 30.13, 15.59. HRMS calcd for C_21_H_19_NO_7_S [M + H]^+^: 430.0955, found 430.0966.

#### 3.4.11. Rhein-L-Val-OMe (**3k**): (R)-Methyl 2-(4,5-Dihydroxy-9,10-dioxo-9,10-dihydroanthracene-2-carboxamido)-4-methylbutanoate

Yellow solid; yield: 93%; m.p, 194~195 °C; ^1^H NMR (400 MHz, CDCl_3_) δ 12.04 (s, 1H, anthraquinone-OH), 11.97 (s, 1H, anthraquinone-OH), 8.14 (d, *J* = 1.6 Hz, 1H, anthraquinone-H), 7.90–7.84 (m, 1H, anthraquinone-H), 7.73 (dd, *J* = 10.4, *J* = 4.8 Hz, 2H, anthraquinone-H), 7.34 (dd, *J* = 8.4, *J* = 0.8 Hz, 1H, anthraquinone-H), 6.79 (d, *J* = 8.4 Hz, 1H, CONH), 4.79 (dd, *J* = 8.8, *J* = 4.8 Hz, 1H, acylamino-CH), 3.81 (s, 3H, CH_3_), 2.40–2.28 (m, 1H, acylamino-CHC*H*), 1.04 (dd, *J* = 6.8, *J* = 5.6 Hz, 6H, CH(CH_3_)_2_). ^13^C-NMR (101 MHz, CDCl_3_) δ 192.66, 181.04, 172.19, 164.87, 162.81, 162.67, 142.00, 137.73, 134.09, 133.43, 125.03, 123.36, 120.45, 117.56, 117.48, 115.79, 57.82, 52.48, 31.64, 19.03, 18.07. HRMS calcd for C_21_H_19_NO_7_ [M + H]^+^: 398.1234, found 398.1241

#### 3.4.12. Rhein-D-Leu-OMe (**3l**): (S)-Methyl 2-(4,5-dihydroxy-9,10-dioxo-9,10-dihydroanthracene-2-carboxamido)-4-methylpentanoate

Yellow solid; yield: 90%; m.p, 177~178 °C; ^1^H NMR (400 MHz, CDCl_3_) δ 12.01 (s, 1H, anthraquinone-OH), 11.96 (s, 1H, anthraquinone-OH), 8.11 (d, *J* = 1.6 Hz, 1H, anthraquinone-H), 7.86 (d, *J* = 7.2 Hz, 1H, anthraquinone-H), 7.73 (dd, *J* = 11.2, *J* = 4.8 Hz, 2H, anthraquinone-H), 7.34 (d, *J* = 8.4 Hz, 1H, anthraquinone-H), 6.78 (d, *J* = 8.4 Hz, 1H, CONH), 4.92–4.80 (m, 1H, acylamino-CH), 3.80 (s, 3H, CH_3_), 1.84–1.68 (m, 3H, acylamino-CHC*H*_2_C*H*), 1.01 (dd, *J* = 5.6, *J* = 4.0 Hz, 6H, CH(C*H*_3_)_2_). ^13^C-NMR (101 MHz, CDCl_3_) δ 192.62, 181.06, 173.28, 164.63, 162.80, 162.65, 141.71, 137.71, 134.01, 133.40, 125.04, 123.49, 120.44, 117.53, 117.41, 115.78, 52.62, 51.47, 41.68, 25.03, 22.85, 21.99. HRMS calcd for C_22_H_21_NO_7_ [M + H]^+^: 412.139, found 412.1395.

#### 3.4.13. Rhein-D-Met-OMe (**3m**): (S)-Methyl 2-(4,5-Dihydroxy-9,10-dioxo-9,10-dihydroanthracene-2-carboxamido)-4-(methylthio)butanoate

Yellow solid; yield: 89%; m.p, 190~193 °C; ^1^H NMR (400 MHz, CDCl_3_) δ 12.02 (s, 1H, anthraquinone-OH), 11.96 (s, 1H, anthraquinone-OH), 8.14 (d, *J* = 1.6 Hz, 1H, anthraquinone-H), 7.86 (d, *J* = 6.8 Hz, 1H, anthraquinone-H), 7.78–7.69 (m, 2H, anthraquinone-H), 7.34 (d, *J* = 7.6 Hz, 1H, anthraquinone-H), 7.22 (d, *J* = 7.6 Hz, 1H, CONH), 5.01–4.94 (m, 1H, acylamino-CH), 3.83 (s, 3H, CH_3_), 2.63 (t, *J* = 7.2 Hz, 2H, C*H*_2_SCH_3_), 2.38–2.17 (m, 2H, acylamino-CHC*H*), 2.15 (s, 3H, SCH_3_). ^13^C-NMR (101 MHz, CDCl_3_) δ 192.63, 181.00, 172.13, 164.65, 162.81, 162.66, 141.54, 137.73, 134.06, 133.39, 125.03, 123.44, 120.45, 117.60, 117.48, 115.77, 52.87, 52.43, 31.25, 30.14, 15.58. HRMS calcd for C_21_H_19_NO_7_S [M + H]^+^: 430.0955, found 430.0958.

#### 3.4.14. Rhein-L-Trp-OMe (**3n**): (R)-Methyl 2-(4,5-Dihydroxy-9,10-dioxo-9,10-dihydroanthracene-2-carboxamido)-3-(1H-indol-3-yl)propanoate

Yellow solid; yield: 83%; m.p, 173~175 °C; ^1^H NMR (400 MHz, DMSO-*d_6_*) δ 11.89 (s, 2H, anthraquinone-OH), 10.87 (s, 1H, indole-NH), 9.35 (d, *J* = 7.6 Hz, 1H, indole-H), 8.12 (d, *J* = 1.6 Hz, 1H, anthraquinone-H), 7.81 (d, *J* = 8.0 Hz, 1H, anthraquinone-H), 7.76–7.71 (m, 2H, anthraquinone-H), 7.57 (s, 1H, indole-H), 7.40 (dd, *J* = 8.4, *J* = 1.2 Hz, 1H, anthraquinone-H), 7.34 (d, *J* = 8.0 Hz, 1H, indole-H), 7.23 (d, *J* = 2.4 Hz, 1H, indole-H), 7.07 (s, 1H, indole-H), 7.00 (s, 1H, CONH), 4.76–7.70 (m, 1H, acylamino-CH), 3.67 (s, 3H, CH_3_), 3.34–3.26 (m, 2H, *CH*_2_-indole). ^13^C-NMR (101 MHz, DMSO-*d_6_*) δ 191.93, 181.46, 172.61, 164.77, 161.85, 161.53, 141.27, 138.02, 136.59, 134.05, 133.71, 127.50, 124.97, 124.15, 123.11, 121.50, 119.90, 118.94, 118.48, 118.21, 116.50, 111.96, 110.31, 54.54, 52.53, 26.97. HRMS calcd for C_27_H_20_N_2_O_7_ [M + H]^+^: 485.1343, found 485.1349.

#### 3.4.15. Rhein-L-Glu(OMe)-OMe (**3o**): (R)-Dimethyl 2-(4,5-Dihydroxy-9,10-dioxo-9,10-dihydroanthracene-2-carboxamido)pentanedioate

Yellow solid; yield: 84%; m.p, 171~173 °C; ^1^H NMR (400 MHz, CDCl_3_) δ 11.95 (s, 1H, anthraquinone-OH), 11.91 (s, 1H, anthraquinone-OH), 8.09 (d, *J* = 1.2 Hz, 1H, anthraquinone-H), 7.81 (d, *J* = 7.2 Hz, 1H, anthraquinone-H), 7.70 (dd, *J* = 10.4, *J* = 4.8 Hz, 2H, anthraquinone-H), 7.50 (d, *J* = 7.2 Hz, 1H, anthraquinone-H), 7.30 (d, *J* = 8.4 Hz, 1H, CONH), 4.85–4.78 (m, 1H, acylamino-CH), 3.81 (d, *J* = 8.4 Hz, 3H, CH_3_), 3.73 (s, 3H, CH_3_), 2.58–2.50 (m, 2H, CHCH_2_C*H*_2_), 2.33–2.28 (m, 2H, CHC*H*_2_). ^13^C-NMR (101 MHz, CDCl_3_) δ 192.52, 180.84, 173.73, 172.02, 164.86, 162.71, 162.54, 141.48, 137.67, 133.89, 133.31, 124.94, 123.43, 120.37, 117.60, 117.44, 115.68, 52.82, 52.69, 52.14, 30.26, 26.80. HRMS calcd for C_22_H_19_NO_9_ [M + H]^+^: 442.1133, found 442.1136.

#### 3.4.16. Rhein-D-Tyr-OEt (**3p**): (S)-Ethyl 2-(4,5-Dihydroxy-9,10-dioxo-9,10-dihydroanthracene-2-carboxamido)-3-(4-hydroxyphenyl)propanoate

Yellow solid; yield: 88%; m.p, 221~222 °C; ^1^H NMR (400 MHz, DMSO-*d_6_*) δ 11.89 (s, 2H, anthraquinone-OH), 9.30 (s, 1H, anthraquinone-H) 9.26 (s, 1H, anthraquinone-H), 8.10 (d, *J* = 1.6 Hz, 1H, anthraquinone-H), 7.82 (t, *J* = 8.0 Hz, 1H, anthraquinone-H), 7.78–7.68 (m, 2H, Ar-H), 7.40 (d, *J* = 7.6 Hz, 1H, Ar-H), 7.10 (d, *J* = 8.4 Hz, 2H, Ar-H), 6.67 (s, 1H, Ar-H), 6.65 (s, H, CONH), 4.66–4.53 (m, 1H, acylamino-CH), 4.11 (d, *J* = 7.2 Hz, 2H, acylamino-CHC*H*_2_), 3.07–3.01 (m, 2H, C*H*_2_CH_3_), 1.16 (t, *J* = 7.2 Hz, 3H, CH_3_). ^13^C-NMR (101 MHz, DMSO-*d_6_*) δ 191.98, 181.59, 171.89, 164.81, 161.86, 161.53, 156.44, 141.36, 138.05, 134.18, 133.82, 130.49, 127.91, 125.01, 123.03, 119.92, 118.35, 118.14, 116.62, 115.51, 61.13, 55.38, 35.92, 14.50. HRMS calcd for C_26_H_21_NO_8_ [M + H]^+^: 476.134, found 476.1346.

#### 3.4.17. Rhein-D-Ala-OEt (**3q**) (S)-Ethyl 2-(4,5-Dihydroxy-9,10-dioxo-9,10-dihydroanthracene-2-carboxamido)propanoate

Yellow solid; yield: 89%; m.p, 236~237 °C; ^1^H NMR (400 MHz, CDCl_3_) δ 12.03 (s, 1H, anthraquinone-OH), 11.98 (s, 1H, anthraquinone-OH), 8.14 (s, 1H, anthraquinone-H), 7.87 (s, 1H, anthraquinone-H), 7.75 (d, *J* = 15.2 Hz, 2H, anthraquinone-H), 7.34 (d, *J* = 8.0 Hz, 1H, anthraquinone-H), 6.93 (s, 1H, CONH), 4.79 (s, 1H, acylamino-CH), 4.28 (d, *J* = 6.8 Hz, 2H, *CH*_2_CH_3_), 1.57 (d, *J* = 6.4 Hz, 3H, acylamino-CHC*H*_3_), 1.33 (t, *J* = 6.4 Hz, 3H, CH_3_). ^13^C-NMR (101 MHz, DMSO*-d_6_*) δ 191.96, 181.54, 172.76, 164.57, 161.87, 161.58, 141.33, 138.05, 134.11, 133.76, 125.00, 123.13, 119.92, 118.26, 118.19, 116.56, 61.07, 49.13, 17.02, 14.55. HRMS calcd for C_20_H_17_NO_7_ [M + H]^+^: 384.1078, found 384.1079.

#### 3.4.18. Rhein-D-Phe-OMe (**3r**) (S)-Methyl 2-(4,5-Dihydroxy-9,10-dioxo-9,10-dihydroanthracene-2-carboxamido)-3-phenylpropanoate

Yellow solid; yield: 92%; m.p, 201~202 °C; ^1^H NMR (400 MHz, CDCl_3_) δ 12.03 (s, 1H, anthraquinone-OH), 11.96 (s, 1H, anthraquinone-OH), 8.05 (d, *J* = 1.6 Hz, 1H, anthraquinone-H), 7.86 (dd, *J* = 7.6, *J* = 1.2 Hz, 1H, anthraquinone-H), 7.76–7.69 (m, 1H, anthraquinone-H), 7.66 (d, *J* = 1.6 Hz, 1H, anthraquinone-H), 7.37–7.27 (m, 4H, Ar-H), 7.16 (s, 1H, anthraquinone-H), 7.15 (s, 1H, Ar-H), 6.72 (d, *J* = 7.6 Hz, 1H, CONH), 5.17–5.04 (m, 1H, acylamino-CH), 3.81 (s, 3H, CH_3_), 3.31– 3.25 (m, 2H, acylamino-CH_2_). ^13^C-NMR (101 MHz, CDCl_3_) δ 192.66, 180.95, 171.62, 164.45, 162.80, 162.64, 141.77, 137.73, 135.48, 134.09, 133.42, 129.28, 128.82, 127.47, 125.00, 123.22, 120.43, 117.59, 117.50, 115.77, 53.82, 52.66, 37.85. HRMS calcd for C_25_H_19_NO_7_ [M + H]^+^: 446.1234, found 446.1236.

#### 3.4.19. Rhein-D-Trp-OMe (**3s**) (S)-Methyl 2-(4,5-Dihydroxy-9,10-dioxo-9,10-dihydroanthracene-2-carboxamido)-3-(1H-indol-3-yl)propanoate

Yellow solid; yield: 74%; m.p, 196~198 °C; ^1^H NMR (400 MHz, DMSO-*d_6_*) δ 11.89 (s, 2H, anthraquinone-OH), 10.87 (s, 1H, indole-NH), 9.35 (d, *J* = 7.6 Hz, 1H, indole-H), 8.12 (d, *J* = 1.2 Hz, 1H, anthraquinone-H), 7.82 (t, *J* = 8.0 Hz, 1H, anthraquinone-H), 7.78–7.70 (m, 2H, anthraquinone-H), 7.58 (d, *J* = 8.0 Hz, 1H, indole-H), 7.40 (d, *J* = 7.6 Hz, 1H, anthraquinone-H), 7.34 (d, *J* = 8.0 Hz, 1H, indole-H), 7.23 (d, *J* = 2.0 Hz, 1H, indole-H), 7.07 (t, *J* = 7.2 Hz, 1H, indole-H), 7.00 (t, *J* = 7.2 Hz, 1H, CONH), 4.78–4.68 (m, 1H, acylamino-CH), 3.67 (s, 3H, CH_3_), 3.33–3.25 (m, 2H, *CH*_2_-indole). ^13^C-NMR (101 MHz, DMSO-*d_6_*) δ 191.99, 181.59, 172.58, 164.81, 161.87, 161.54, 141.30, 138.05, 136.59, 134.16, 133.82, 127.51, 125.01, 124.13, 123.11, 121.49, 119.93, 118.94, 118.48, 118.34, 118.18, 116.63, 111.96, 110.32, 54.53, 52.53, 26.96. HRMS calcd for C_27_H_20_N_2_O_7_ [M + H]^+^: 485.1343, found 485.1350.

#### 3.4.20. Rhein-L-Tyr-OMe (**3t**) (R)-Methyl 2-(4,5-Dihydroxy-9,10-dioxo-9,10-dihydroanthracene-2-carboxamido)-3-(4-hydroxyphenyl)propanoate

Yellow solid; yield: 76%; m.p, 233~234 °C; ^1^H NMR (400 MHz, DMSO-*d_6_*) δ 11.90 (s, 2H, anthraquinone-OH), 9.31 (s, 1H, anthraquinone-H), 9.27 (s, 1H, anthraquinone-H), 8.09 (d, *J* = 1.6 Hz, 1H, anthraquinone-H), 7.88–7.80 (m, 1H, anthraquinone-H), 7.76 (dd, *J* = 7.6, *J* = 1.0 Hz, 1H, anthraquinone-H), 7.71 (d, *J* = 1.6 Hz, 1H, Ar-H), 7.41 (dd, *J* = 8.4, *J* = 0.8 Hz, 1H, Ar-H), 7.09 (d, *J* = 8.4 Hz, 2H, Ar-H), 6.67 (s, H, Ar-OH), 6.65 (s, H, CONH), 4.66–4.60 (m, 1H, acylamino-CH), 3.66 (s, 3H, CH_3_), 3.08–3.02 (m, 2H, acylamino-CHC*H*_2_). ^13^C-NMR (101 MHz, DMSO-*d_6_*) δ 191.92, 181.64, 172.37, 164.80, 161.91, 161.64, 156.44, 141.27, 138.02, 134.21, 133.84, 130.46, 127.94, 125.04, 123.10, 119.89, 118.40, 118.06, 116.66, 115.54, 55.27, 52.50, 35.86. HRMS calcd for C_25_H_19_NO_8_ [M + H]^+^: 462.1183, found 462.1186.

### 3.5. In Vitro Antifungal Bioassay

The primary antifungal activities of all the target compounds against *Rhizoctonia solani*, *Sclerotinia sclerotiorum*, *Bipolaris maydis* and *Phytophthora capsici* were evaluated using a mycelium growth rate method reported in our previously published literature report [24,25]. Briefly, a solution of the tested compound (25 or 10 mmol) in 5 mL of sterile water containing 0.1% Tween 80 and 0.1 mL of DMSO was fully mixed with 45 mL of 50 °C melted PDA agar to provide a culture medium containing a 0.5 mM or 0.2 mM tested compound and 0.2% (*v*/*v*) DMSO. Then, it was poured into a sterilized Petri dish (ca. 16 mL on each plate). The control blank assay was performed with 0.2% DMSO in sterile aqueous 1% Tween 80. A biofungicide phenazine-1-carboxylic acid (PCA) was used as the positive control. A hyphal plug (d = 6 mm) was taken from the growing margin of the test fungus on each subcultured Petri dish and placed on the amended PDA. Each experiment was repeated three times. The inoculated Petri dishes were kept in an incubator at 25 °C for 72 h, after which, the diameter of each fungal colony was measured, and the percentage inhibition was calculated. The relative inhibitory rates (I) of the tested compounds were calculated using the following formula: I (%) = [(C − T)/(C − 6)] × 100, where C represents the average colony diameter (mm) during the blank assay, and T represents the average colony diameter (mm) during the test.

The target compounds with higher initial activities were further assayed to determine the EC_50_ values according to the method described above. Based on the screening results, a series of test concentrations of the compound was set and evaluated to determine its inhibitory rate against the fungi. The log dose–response curves allowed for the determination of the EC_50_ value for the bioassay using probit analysis.

### 3.6. In Vivo Protective and Curative Antifungal Bioassay

The control efficacy (protective and curative activity) of compound **3c** against powdery mildew in wheat was assessed with pot experiments according to the method described in the literature with some modifications [26]. First, wheat seeds were grown in plastic pots (20 cm diameter) with about 15 plants per pot. After three weeks, plants in the three-leaf stage were used in antifungal activity experiments. For the protection assay, compound **3c** solutions as well as the positive control physcion with different concentrations (0.4 and 0.2 mM) (containing 0.1% Tween 80 as the surfactant) were sprayed on the leaves of wheat on the first day. After 24 h, powdery mildew spores were inoculated into the leaves of wheat. Wheat seedlings sprayed with water were used as negative controls. Then, the plants were placed in a greenhouse at 25 °C with 100% relative humidity. After 7 days of greenhouse culture, the disease index of the wheat seedlings was measured. The grading standard of powdery mildew in wheat was used according to the method presented in the literature: Disease index (CK or PT) = ∑ (the number of leaves at each grade × the corresponding grade)/(the total number of leaves × the superlative grade). The protective efficacy of the tested compound was calculated according to the following formula: Relative control efficacy I (%) = (CK − PT)/CK × 100, where CK is the disease index of the negative control and PT is the disease index of the treatment group. For the curative assay, the powdery mildew spores were inoculated into the leaves for 24 h before the leaves were sprayed with the solutions under examination. The rest of the procedures were the same as the above.

### 3.7. Statistical Analysis

The collected data were analyzed using GraphPad Prism (Version 8.3.0) software.

## 4. Conclusions

In summary, a series of rhein–amino acid ester conjugates were obtained in good yields by conjugating rhein with L- or D-amino acid esters via an amide bond, and their structures were confirmed by ^1^H-NMR, ^13^C-NMR and HRMS. The bioassay results revealed that most of the conjugates exhibited potent inhibitory activity against *R. solani* and *S. sclerotiorum*. In particular, conjugate **3c** had the highest antifungal activity against *R. solani*, with an EC_50_ value of 0.125 mM. For *S. sclerotiorum*, conjugate **3m** showed the highest antifungal activity, with an EC_50_ value of 0.114 mM. Satisfactorily, conjugate **3c** exhibited better protective effects than those of the positive control physcion against wheat powdery mildew. This research provides support for rhein–amino acid esters conjugates as potential antifungal agents for plant fungal diseases.

## Figures and Tables

**Table:** 

Entry	Reactant (Amino Acid Ester)	Product (3)	Yield (%)
**3a**	H-Gly-OMe·HCl	Rhein-Gly-OMe	78
**3b**	H-Gly-OEt·HCl	Rhein-Gly-OEt	82
**3c**	H-L-Ala-OEt·HCl	Rhein-L-Ala-OEt	90
**3d**	H-L-Leu-OMe·HCl	Rhein-L-Leu-OMe	92
**3e**	H-L-Leu-OEt·HCl	Rhein-L-Leu-OEt	92
**3f**	H-L-Ile-OMe·HCl	Rhein-L-Ile-OMe	92
**3g**	H-L-Phe-OEt·HCl	Rhein-L-Phe-OEt	94
**3h**	H-L-Phe-Tyr·HCl	Rhein-L-Tyr-OEt	87
**3i**	H-D-Val-OMe·HCl	Rhein-D-Val-OMe	95
**3j**	H-L-Met-OMe·HCl	Rhein-L-Met-OMe	90
**3k**	H-L-Val-OMe·HCl	Rhein-L-Val-OMe	93
**3l**	H-D-Leu-OMe·HCl	Rhein-D-Leu-OMe	90
**3m**	H-D-Met-OMe·HCl	Rhein-D-Met-OMe	89
**3n**	H-L-Trp-OMe·HCl	Rhein-L-Trp-OMe	83
**3o**	H-L-Glu(OMe)-OMe·HCl	Rhein-L-Glu(OMe)-OMe	84
**3p**	H-D-Tyr-OMe·HCl	Rhein-D-Tyr-OEt	88
**3q**	H-D-Ala-OEt·HCl	Rhein-D-Ala-OEt	89
**3r**	H-D-Phe-OMe·HCl	Rhein-D-Phe-OMe	92
**3s**	H-D-Typ-OMe·HCl	Rhein-D-Trp-OMe	74
**3t**	H-L-Tyr-OMe·HCl	Rhein-L-Tyr-OMe	76

**Table 1:** EC_50_ values of effective compounds against *R. solani* and *S. sclerotiorum* in vitro (mM).

Compd.	Conjugated Amino Acid Ester	*R. solani*	*S. sclerotiorum*
**3a**	Gly-OMe	0.157	0.486
**3c**	L-Ala-OEt	0.125	0.156
**3d**	L-Leu-OMe	0.152	0.210
**3f**	L-Ile-OMe	0.153	0.132
**3h**	L-Tyr-OEt	0.167	0.155
**3i**	D-Val-OMe	0.185	0.192
**3m**	D-Met-OMe	0.159	0.114
**3n**	L-Trp-OMe	0.176	0.457
**3q**	D-Ala-OEt	0.154	0.179
**3r**	D-Phe-OMe	0.147	0.129
**3t**	L-Tyr-OMe	0.197	0.327

**Table 2:** Protective and curative activities of compound **3c** in vivo.

Compd.	Concentration (mM)	Curative Activity		Protective Activity	
		Disease Index (%)	Control Efficiency (%)	Disease Index (%)	Control Efficiency (%)
**3c**	0.4	12.9	84.4 ± 2.9	33.3	66.7 ± 3.7
	0.2	32.1	61.2 ± 3.0	46.2	53.8 ± 3.4
Physcion	0.4	10.4	87.4 ± 2.4	45.8	54.2 ± 2.9
	0.2	27.1	67.2 ± 2.5	61.6	38.4 ± 3.3
Control	-	82.7	-	100	-

## Data Availability

Data is contained within the article or the Supplementary Materials.

## Supplementary Materials

The following supporting information can be downloaded at: https://www.mdpi.com/article/10.3390/molecules28052074/s1, Tables S1 and S2: Fungicidal activity; Figures S1–S60: 1H-NMR, 13C-NMR, and HRMS spectra of compounds **3a**–**3t**.

## Sample Availability

Samples of the compounds are available from the authors.
