# Supplementary material for: Rhein–Amino Acid Ester Conjugates as Potential Antifungal Agents: Synthesis and Biological Evaluation
Source: Molecules. 2023 Feb 22;28(5):2074. doi: 10.3390/molecules28052074 (PMC10004406; doi:10.3390/molecules28052074)
Supplement: Supplementary file 1 [file molecules-28-02074-s001.zip › molecules-2183171-supplementary.pdf]

Supplementary Information

Rhein–Amino Acid Ester Conjugates as Potential Antifungal Agents: Synthesis and Biological Evaluation

Shunshun Chen [1,†], Meimei Wang [1,2,†], Linhua Yu [1,2], Jinchao Shi [1,2], Yong Zhang [1,2,3], Yao Tian [1], Li Li [1,2], Xiang Zhu [1,2,3,*] and Junkai Li [1,2,*]

1 Institute of Pesticides, College of Agriculture, Yangtze University, Jingzhou 434025, China
2 Hubei Engineering Technology Center for Pest Forewarning and Management, College of Agriculture, Yangtze University, Jingzhou 434025, China
3 State Key Laboratory Breeding Base of Green Pesticide and Agricultural Bioengineering, Key Laboratory of Green Pesticide and Agricultural Bioengineering, Ministry of Education, Guizhou University, Guiyang 550025, China
* Correspondence: xiangzhu1992@yangtzeu.edu.cn (X.Z.); junkaili@yangtzeu.edu.cn (J.L.); Tel./Fax: +86-716-8066314 (X.Z.); +86-716-8066767 (J.L.)
† These authors contributed equally to this work.

Table of Contents

Inhibitory rates of target compounds against four pathogenic fungi *in vitro*···**Table S1**

EC_{50} values of tested compounds with regression equation······················**Table S2**

^{1}H-NMR Spectrum of compound **3a**···**Figure S1**

^{13}C-NMR Spectrum of compound **3a**···**Figure S2**

HRMS Spectrum of compound **3a**···**Figure S3**

^{1}H-NMR Spectrum of compound **3b**···**Figure S4**

^{13}C-NMR Spectrum of compound **3b**···**Figure S5**

HRMS Spectrum of compound **3b**···**Figure S6**

^{1}H-NMR Spectrum of compound **3c**···**Figure S7**

^{13}C-NMR Spectrum of compound **3c**···**Figure S8**

HRMS Spectrum of compound **3c**···**Figure S9**

^{1}H-NMR Spectrum of compound **3d**···**Figure S10**

^{13}C-NMR Spectrum of compound **3d**···**Figure S11**

HRMS Spectrum of compound **3d**···**Figure S12**

^{1}H-NMR Spectrum of compound **3e**···**Figure S13**

^{13}C-NMR Spectrum of compound **3e**···**Figure S14**

HRMS Spectrum of compound **3e**···**Figure S15**

^{1}H-NMR Spectrum of compound **3f**···**Figure S16**

^{13}C-NMR Spectrum of compound **3f**···**Figure S17**

HRMS Spectrum of compound **3f**···**Figure S18**

^{1}H-NMR Spectrum of compound **3g**···**Figure S19**

^{13}C-NMR Spectrum of compound **3g**···**Figure S20**

HRMS Spectrum of compound **3g**···**Figure S21**

^{1}H-NMR Spectrum of compound **3h**···**Figure S22**

^{13}C-NMR Spectrum of compound **3h**···**Figure S23**

HRMS Spectrum of compound **3h**···**Figure S24**

^{1}H-NMR Spectrum of compound **3i**···**Figure S25**

^{13}C-NMR Spectrum of compound **3i**···**Figure S26**

HRMS Spectrum of compound **3i**······**Figure S27**

^{1}H-NMR Spectrum of compound **3j**······**Figure S28**

^{13}C-NMR Spectrum of compound **3j**······**Figure S29**

HRMS Spectrum of compound **3j**······**Figure S30**

^{1}H-NMR Spectrum of compound **3k**······**Figure S31**

^{13}C-NMR Spectrum of compound **3k**······**Figure S32**

HRMS Spectrum of compound **3k**······**Figure S33**

^{1}H-NMR Spectrum of compound **3l**······**Figure S34**

^{13}C-NMR Spectrum of compound **3l**······**Figure S35**

HRMS Spectrum of compound **3l**······**Figure S36**

^{1}H-NMR Spectrum of compound **3m**······**Figure S37**

^{13}C-NMR Spectrum of compound **3m**······**Figure S38**

HRMS Spectrum of compound **3m**······**Figure S39**

^{1}H-NMR Spectrum of compound **3n**······**Figure S40**

^{13}C-NMR Spectrum of compound **3n**······**Figure S41**

HRMS Spectrum of compound **3n**······**Figure S42**

^{1}H-NMR Spectrum of compound **3o**······**Figure S43**

^{13}C-NMR Spectrum of compound **3o**······**Figure S44**

HRMS Spectrum of compound **3o**······**Figure S45**

^{1}H-NMR Spectrum of compound **3p**······**Figure S46**

^{13}C-NMR Spectrum of compound **3p**······**Figure S47**

HRMS Spectrum of compound **3p**······**Figure S48**

^{1}H-NMR Spectrum of compound **3q**······**Figure S49**

^{13}C-NMR Spectrum of compound **3q**······**Figure S50**

HRMS Spectrum of compound **3q**······**Figure S51**

^{1}H-NMR Spectrum of compound **3r**······**Figure S52**

^{13}C-NMR Spectrum of compound **3r**······**Figure S53**

HRMS Spectrum of compound **3r**······**Figure S54**

^{1}H-NMR Spectrum of compound **3s**······**Figure S55**

^{13}C-NMR Spectrum of compound **3s**······**Figure S56**

HRMS Spectrum of compound **3s**··**Figure S57**

^{1}H-NMR Spectrum of compound **3t**······································**Figure S58**

^{13}C-NMR Spectrum of compound **3t**······································**Figure S59**

HRMS Spectrum of compound **3t**··**Figure S60**

Table S1. Inhibitory rates of target compounds against four pathogenic fungi *in vitro*

Compd.	Average inhibition rate ±SD (%) (n=3)							
	R. solani		*S. sclerotiorum*		*B. maydis*		*P. capsici*	
	0.2 (mM)	0.5 (mM)	0.2 (mM)	0.5 (mM)	0.2 (mM)	0.5 (mM)	0.2 (mM)	0.5 (mM)
**3a**	**63.3±2.7**	**81.7±3.5**	30.3±0.6	55.4±2.7	23.3±1.0	43.1±1.8	34.1±0.5	60.7±1.5
**3b**	47.5±2.4	68.5±2.1	25.1±0.8	50.3±2.2	25.8±2.1	46.3±2.6	23.6±2.4	49.7±2.5
**3c**	**70.8±1.6**	**91.2±2.1**	**62.3±0.9**	83.5±1.9	22.2±2.6	44.7±1.9	20.6±1.6	44.5±1.7
**3d**	**66.4±1.6**	**83.1±2.6**	49.4±1.5	69.4±2.8	20.1±1.3	40.5±1.5	32.3±1.7	51.1±1.9
**3e**	34.9±1.5	52.6±1.1	37.9±0.1	58.1±2.6	23.8±1.5	46.4±2.5	22.7±0.7	45.6±1.2
**3f**	**65.1±2.8**	**83.3±3.7**	**65.4±0.5**	**86.5±1.5**	25.6±2.0	50.3±3.9	**51.8±1.6**	73.2±2.8
**3g**	45.5±3.2	67.5±1.8	13.3±0.8	38.7±2.7	20.3±1.3	41.8±1.4	20.3±1.5	43.2±1.0
**3h**	**60.2±2.3**	**82.1±2.7**	**62.8±1.5**	**81.5±3.0**	24.3±0.8	47.7±1.6	18.4±0.7	40.6±1.4
**3i**	**55.5±2.0**	**73.1±3.7**	**53.5±0.9**	**74.3±0.7**	28.4±1.0	53.7±2.4	43.9±1.2	65.7±2.2
**3j**	32.3±1.1	49.7±2.5	16.3±3.2	43.3±2.1	21.3±2.3	40.5±1.7	20.4±1.0	38.6±0.5
**3k**	42.3±1.0	59.7±2.7	22.8±1.5	43.4±1.6	23.1±1.2	47.6±1.5	27.1±1.4	51.4±1.5
**3l**	48.1±0.6	69.3±2.1	15.2±2.4	33.3±3.5	24.4±1.1	46.3±1.4	24.4±2.2	50.3±3.3
**3m**	**63.4±3.3**	**80.5±3.7**	**77.1±1.4**	**92.3±0.7**	28.2±1.2	53.8±2.6	**53.1±2.2**	74.6±2.9
**3n**	**55.9±1.9**	**76.7±1.5**	31.4±1.6	54.7±2.2	23.5±0.6	47.7±1.1	16.7±0.8	34.2±0.5
**3o**	46.8±3.9	68.5±4.7	18.2±1.9	43.1±2.6	21.2±1.2	43.2±1.1	18.7±1.0	40.7±1.6
**3p**	21.1±3.5	43.2±2.6	18.4±1.5	37.8±1.7	26.9±1.2	50.6±2.8	15.0±1.4	35.4±1.0
**3q**	**63.0±1.7**	**79.5±2.4**	**57.4±0.3**	**74.8±2.5**	27.2±0.7	50.7±1.4	48.4±1.9	69.2±3.9
**3r**	**65.0±2.1**	**85.6±3.8**	**69.8±1.3**	**87.6±3.6**	31.1±1.9	57.8±2.3	**52.1±2.7**	74.3±3.6
**3s**	28.6±2.8	51.1±3.6	<10	22.6±0.3	23.6±0.6	47.4±0.8	19.5±1.1	40.3±1.6
**3t**	**53.5±1.8**	**72.1±2.9**	42.4±1.4	61.5±2.8	26.0±1.3	49.7±1.4	20.3±1.1	46.7±2.1
**Rhein**	22.1±1.3	41.5±1.8	22.5±1.5	39.5±1.6	16.2±1.2	28.2±1.5	22.4±2.4	35.4±2.4
**PCA**	**86.3±0.9**	**100±0.0**	**84.6±1.2**	**100±0.0**	**76.5±1.6**	**96.5±1.1**	**79.8±1.6**	**97.5±1.3**

Table S2. EC_{50} values of tested compounds with regression equation

Fungi	Compd.	Regression equation	R^2	EC_{50} (mM)
R. solani	**3a**	y=6.7028+2.1185x	0.9938	0.157
	**3c**	y=6.8850+2.0847x	0.9837	0.125
	**3d**	y=6.8010+2.1998x	0.9951	0.152
	**3f**	y=6.8741+2.2957x	0.9956	0.153
	**3h**	y=6.7141+2.2075x	0.9956	0.167
	**3i**	y=6.5468+2.1075x	0.9980	0.185
	**3m**	y=6.5922+1.9941x	0.9942	0.159
	**3n**	y=6.6098+2.1356x	0.9993	0.176
	**3q**	y=6.6233+2.0014x	0.9839	0.154
	**3r**	y=6.6921+2.0300x	0.9927	0.147
	**3t**	y=6.4272+2.0245x	0.9983	0.197
	**PCA**	y=7.8846+2.6655x	0.9994	0.083
S. sclerotiorum	**3a**	y=5.3992+1.2755x	0.9928	0.486
	**3c**	y=6.6932+2.1003x	0.9942	0.156
	**3d**	y=6.2483+1.8442x	0.9971	0.210
	**3f**	y=7.2837+2.5992x	0.9922	0.132
	**3h**	y=7.7827+2.1993x	0.9976	0.155
	**3i**	y=7.4894+2.0756x	0.9988	0.192
	**3m**	y=7.1205+2.2522x	0.9957	0.114
	**3n**	y=5.4753+1.3958x	0.9916	0.457
	**3q**	y=6.3567+1.8183x	0.9888	0.179
	**3r**	y=6.9448+2.1896x	0.9924	0.129
	**3t**	y=5.7095+1.4617x	0.9924	0.327
	**3a**	y=8.1056+2.9426x	0.9773	0.088

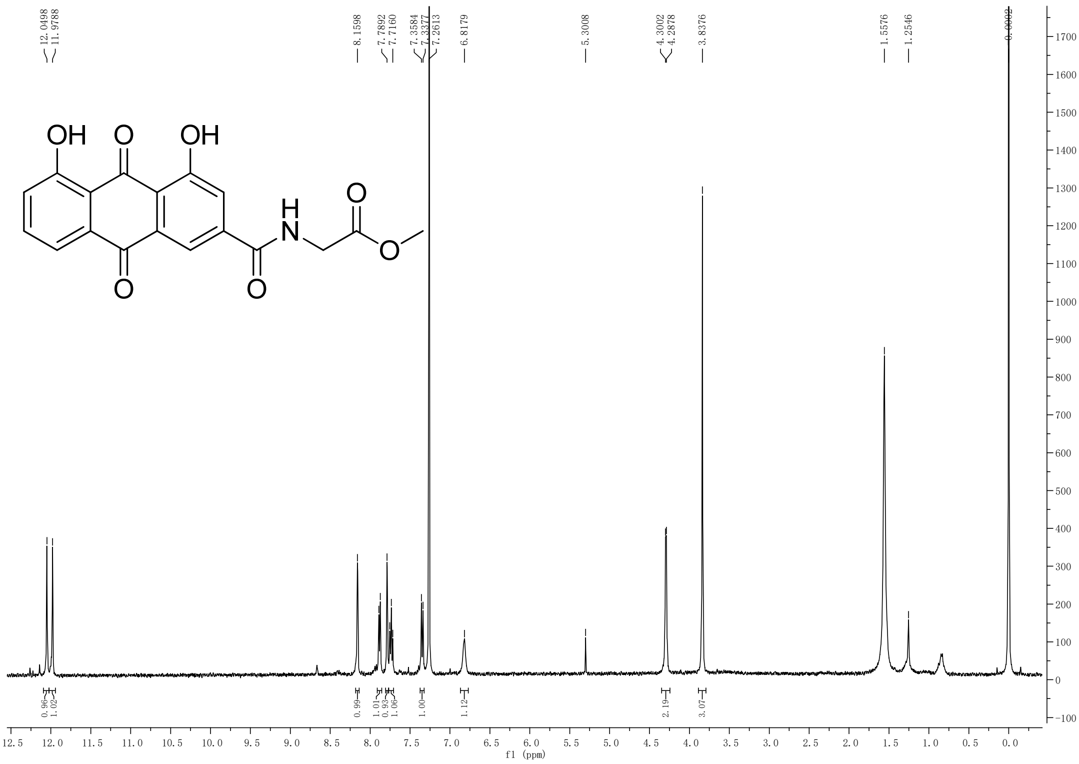

Figure S1. ^{1}H-NMR Spectrum of compound **3a**

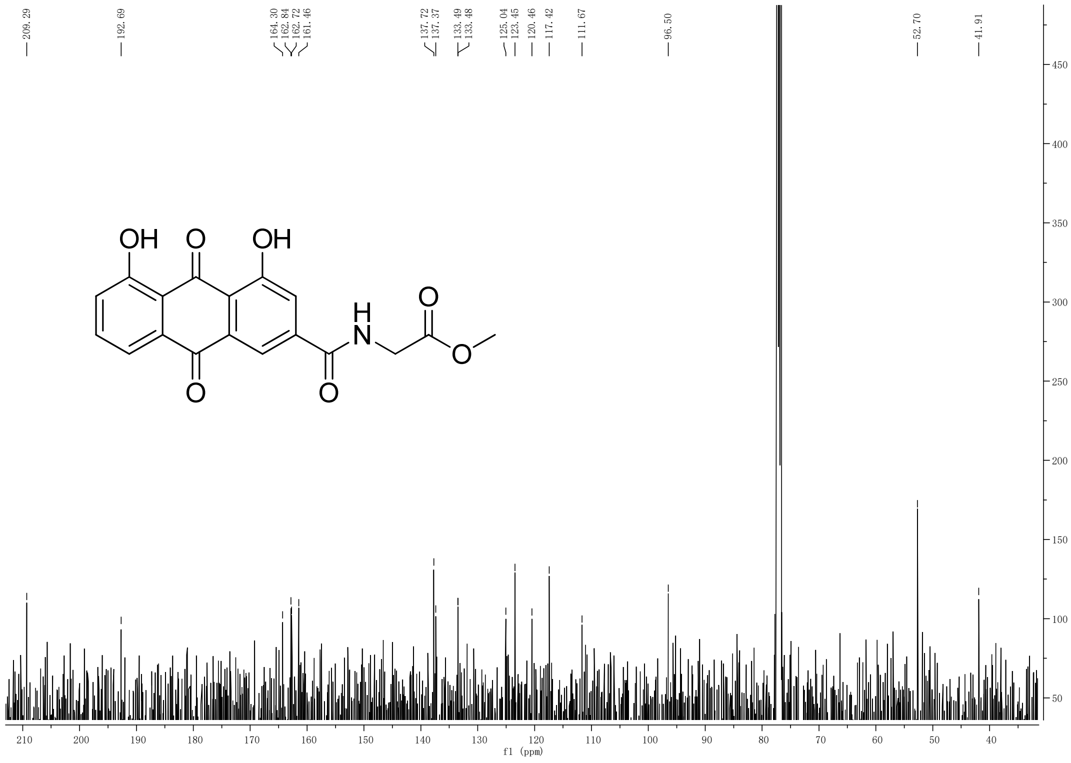

Figure S2. ^{13}C-NMR Spectrum of compound **3a**

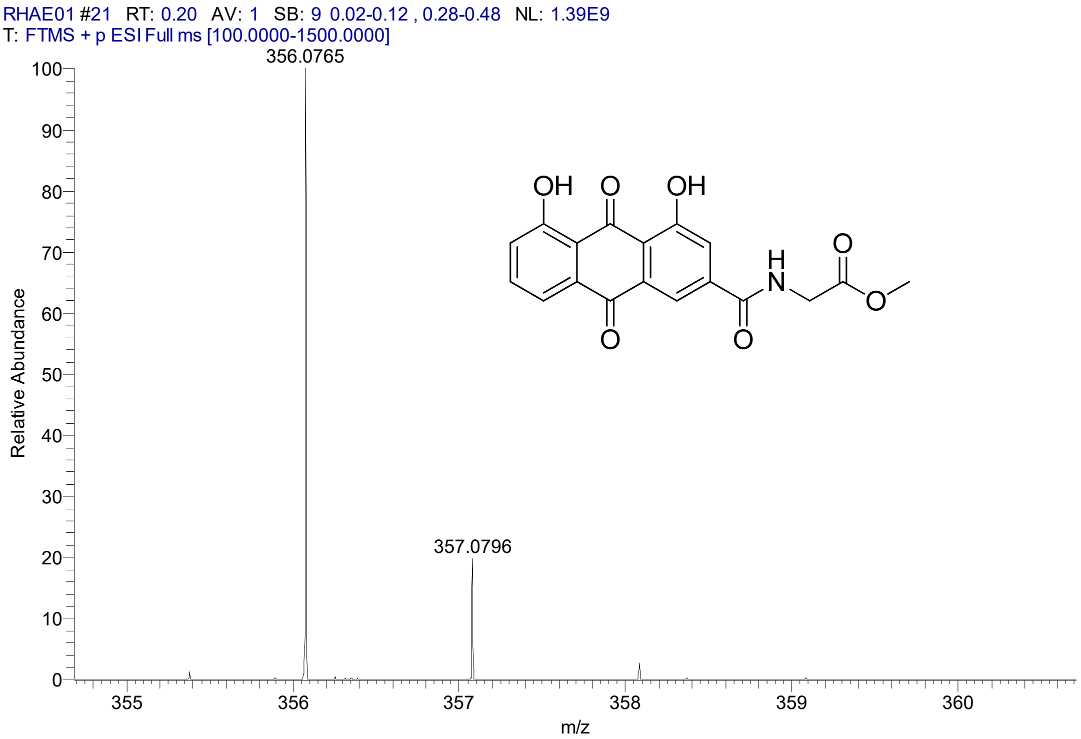

Figure S3. HRMS Spectrum of compound **3a**

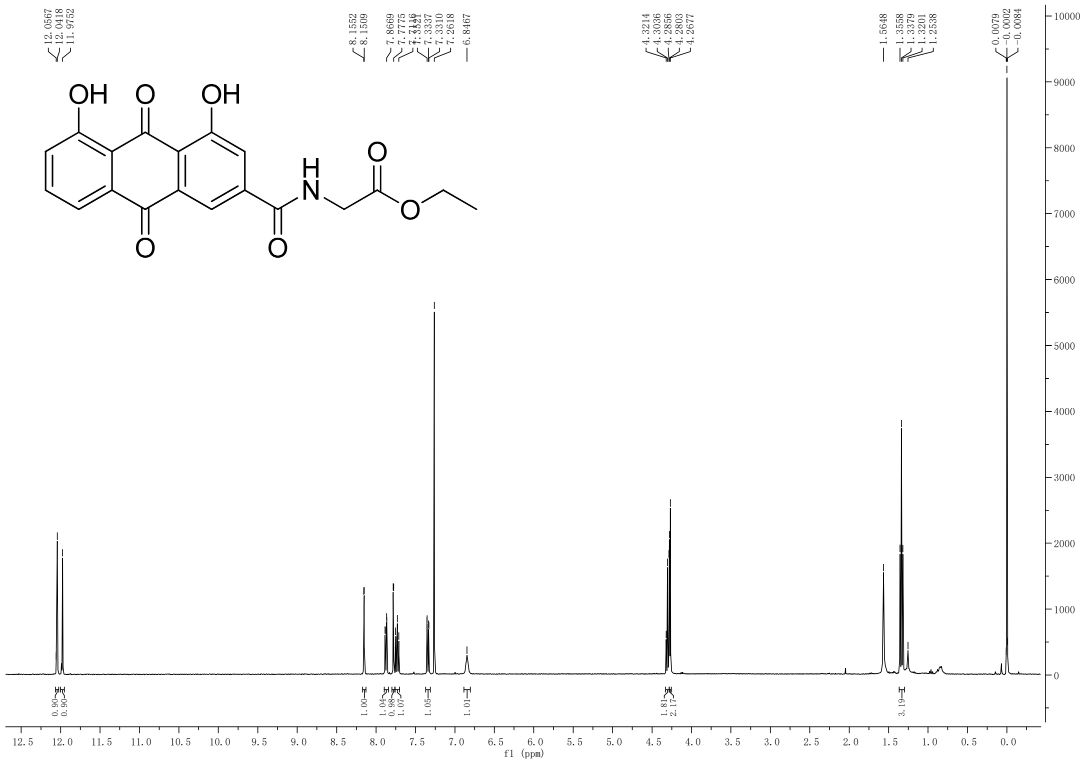

Figure S4. ^{1}H-NMR Spectrum of compound **3b**

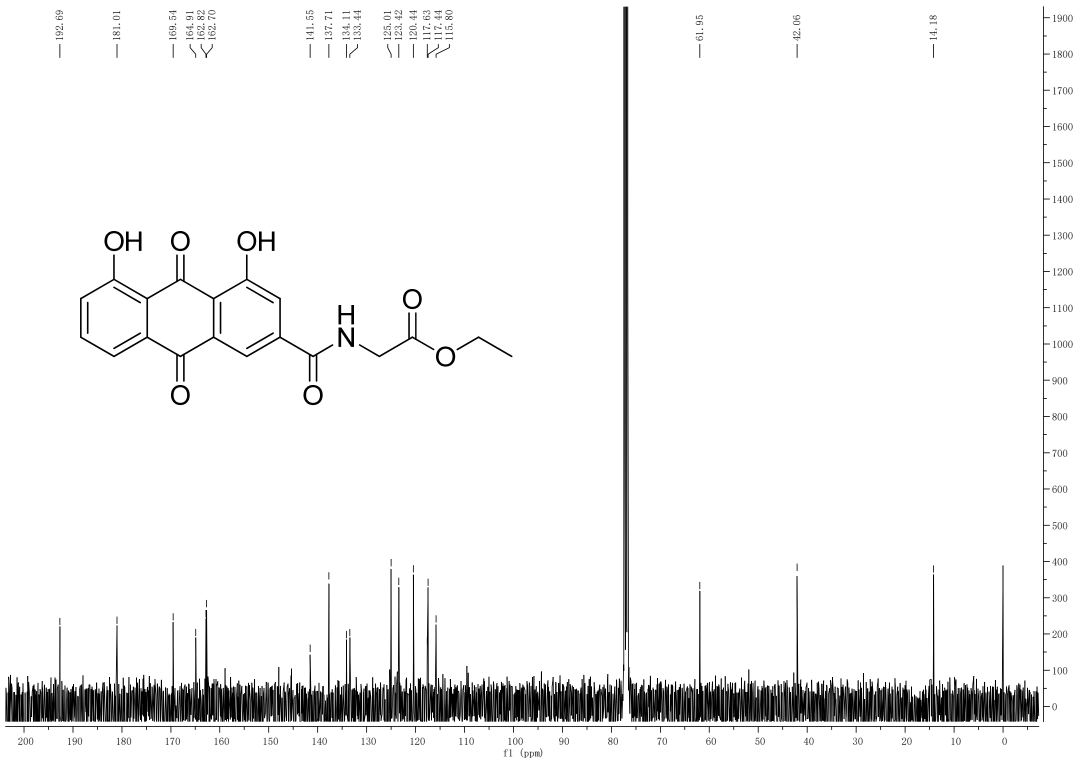

Figure S5. ^{13}C-NMR Spectrum of compound **3b**

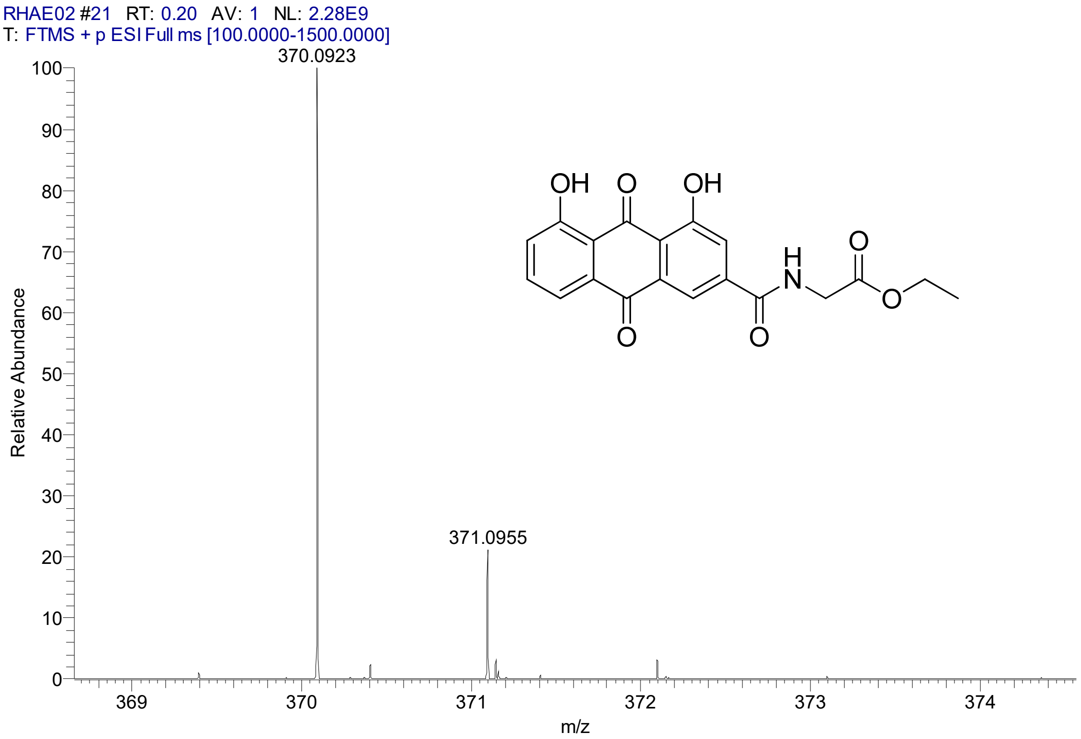

Figure S6. HRMS Spectrum of compound **3b**

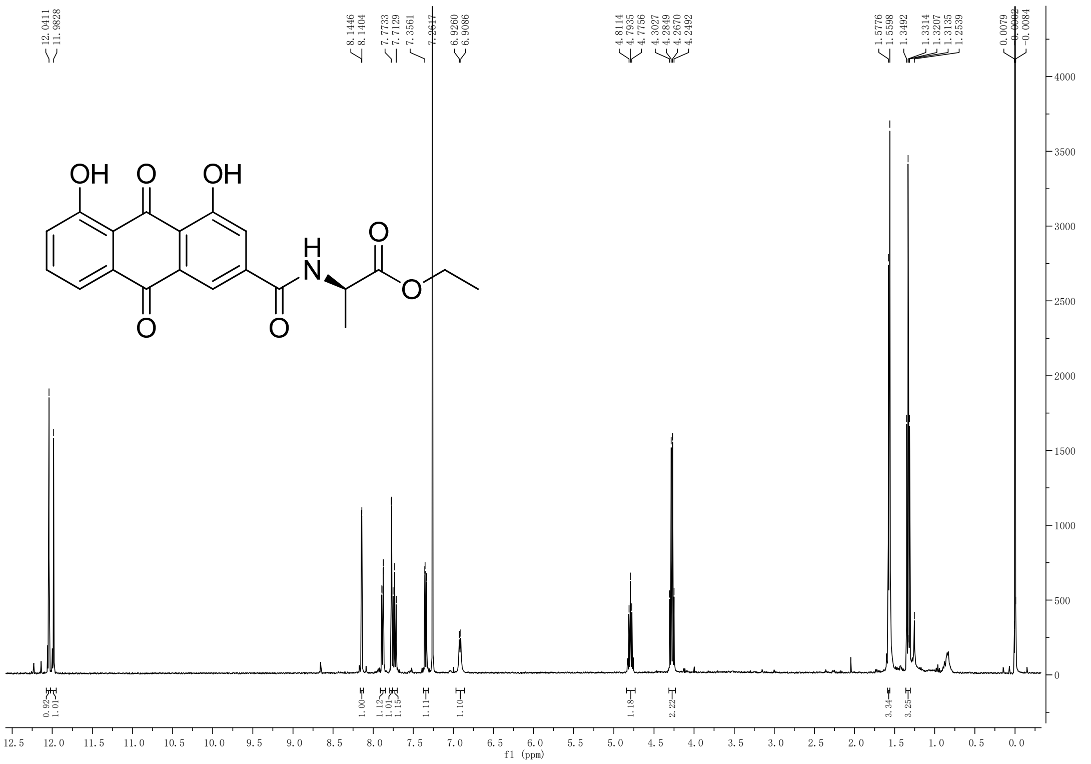

Figure S7. ^{1}H-NMR Spectrum of compound **3c**

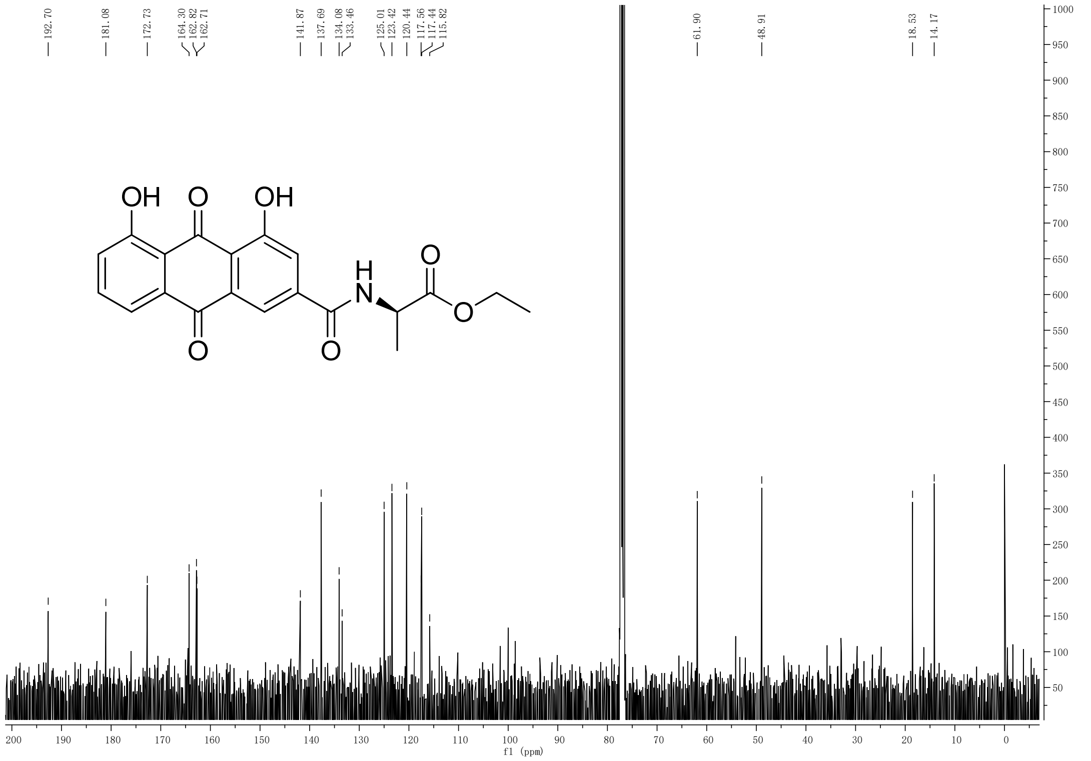

Figure S8. ^{13}C-NMR Spectrum of compound **3c**

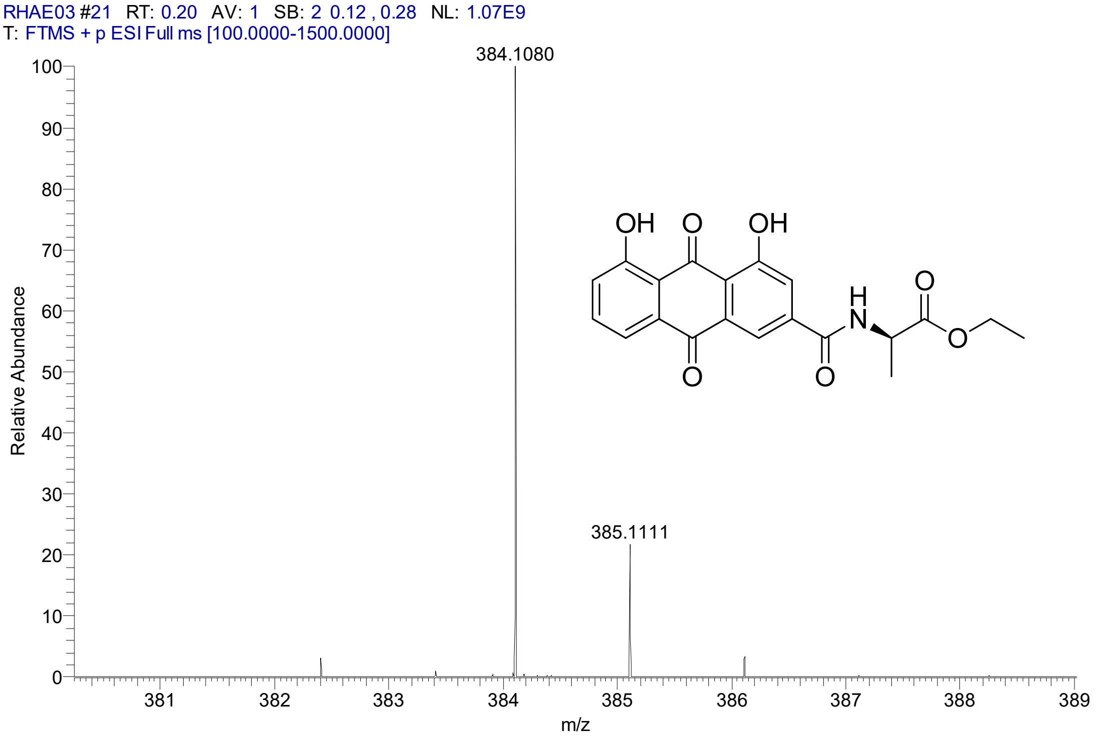

Figure S9. HRMS Spectrum of compound **3c**

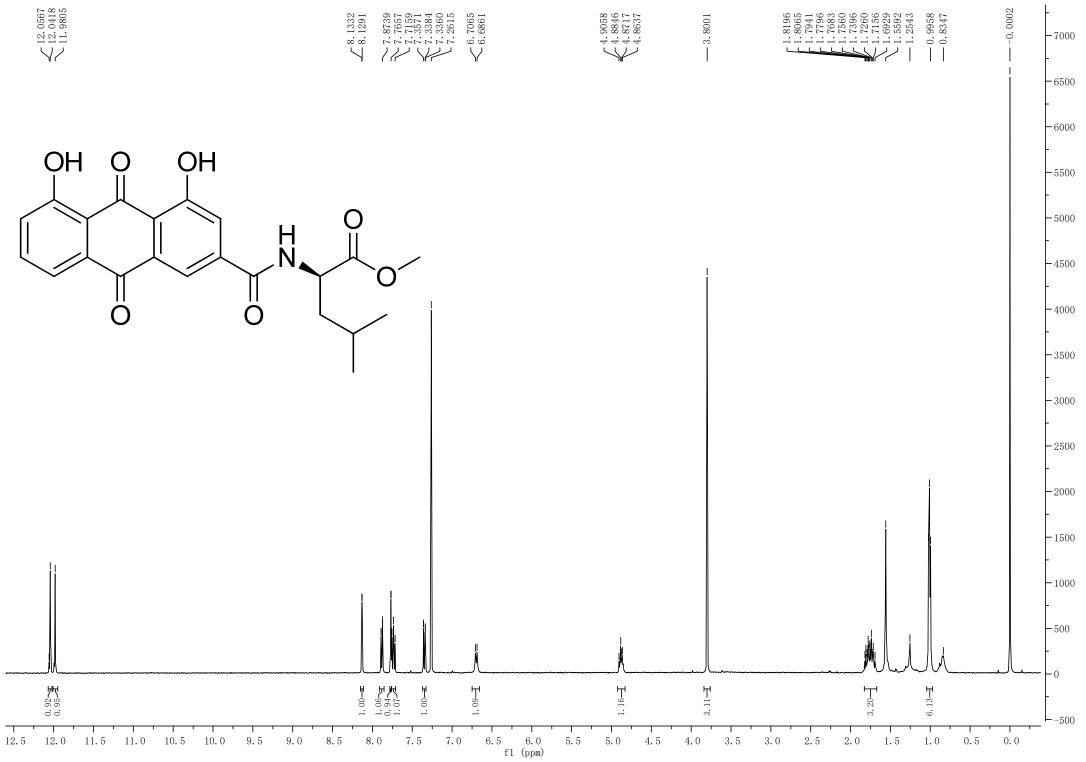

Figure S10. ^{1}H-NMR Spectrum of compound **3d**

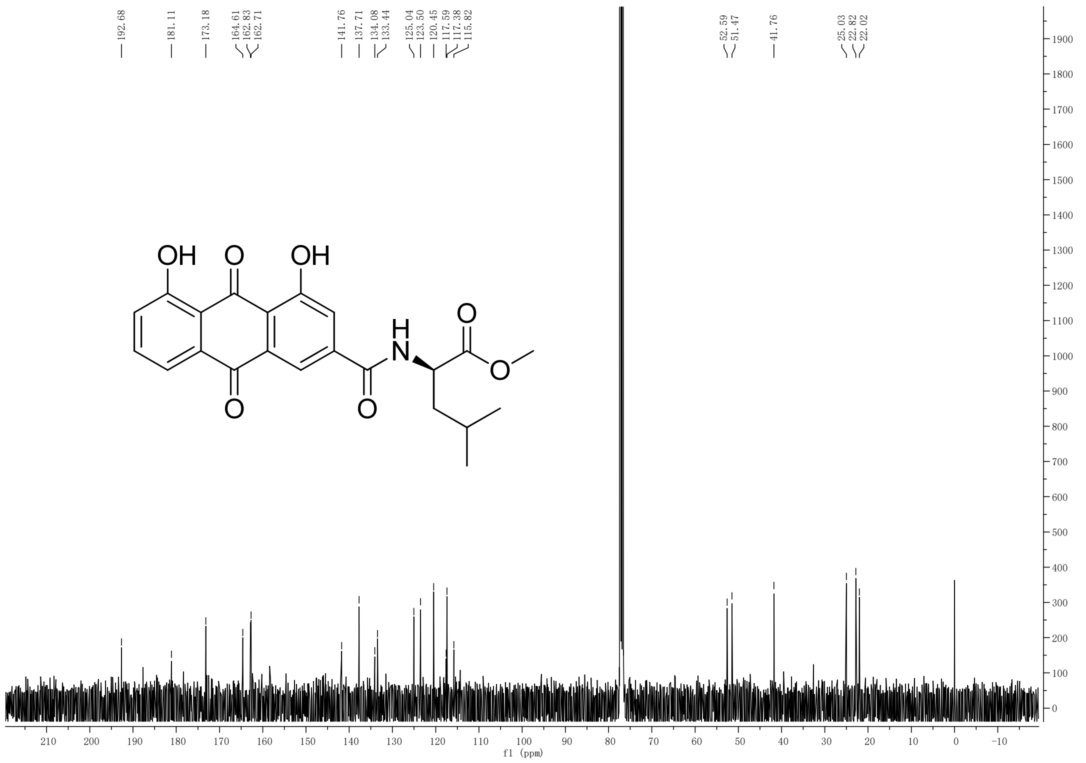

Figure S11. ^{13}C-NMR Spectrum of compound **3d**

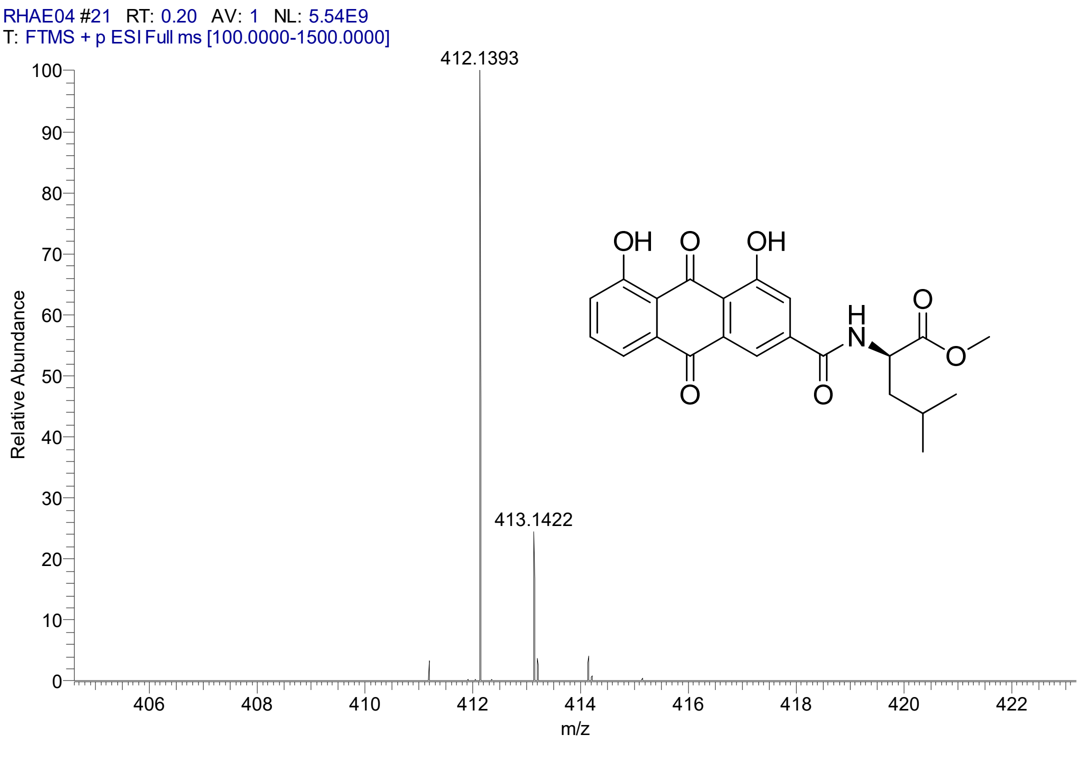

Figure S12. HRMS Spectrum of compound **3d**

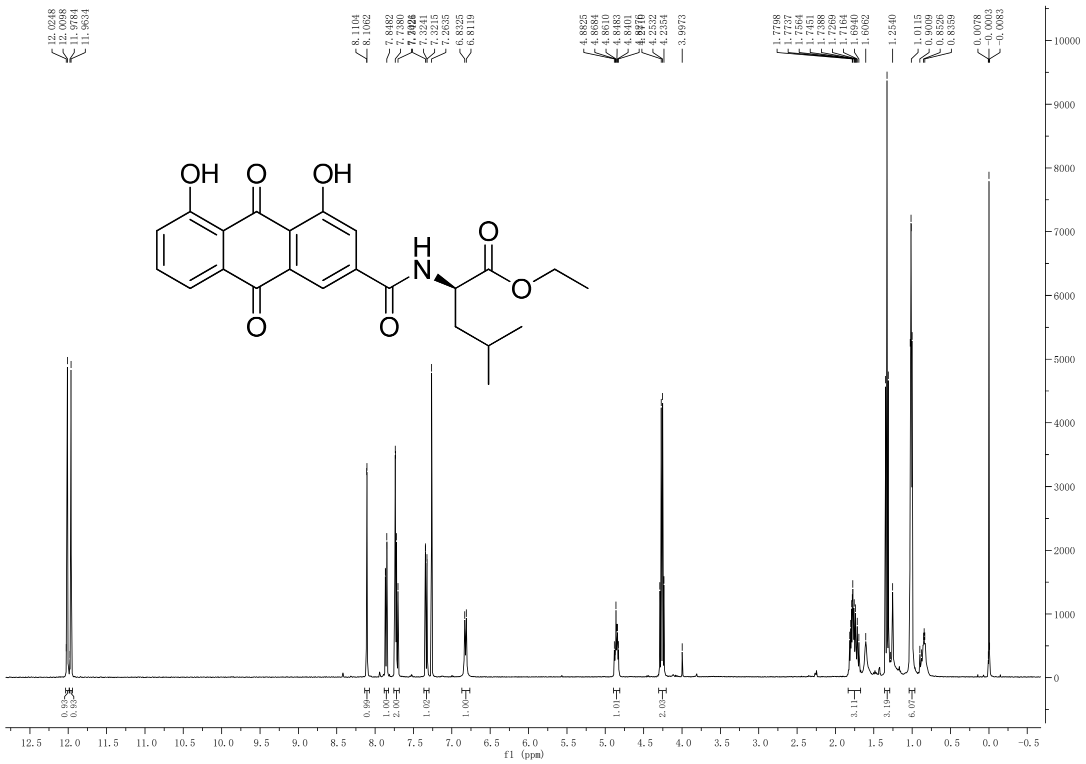

Figure S13. ^{1}H-NMR Spectrum of compound **3e**

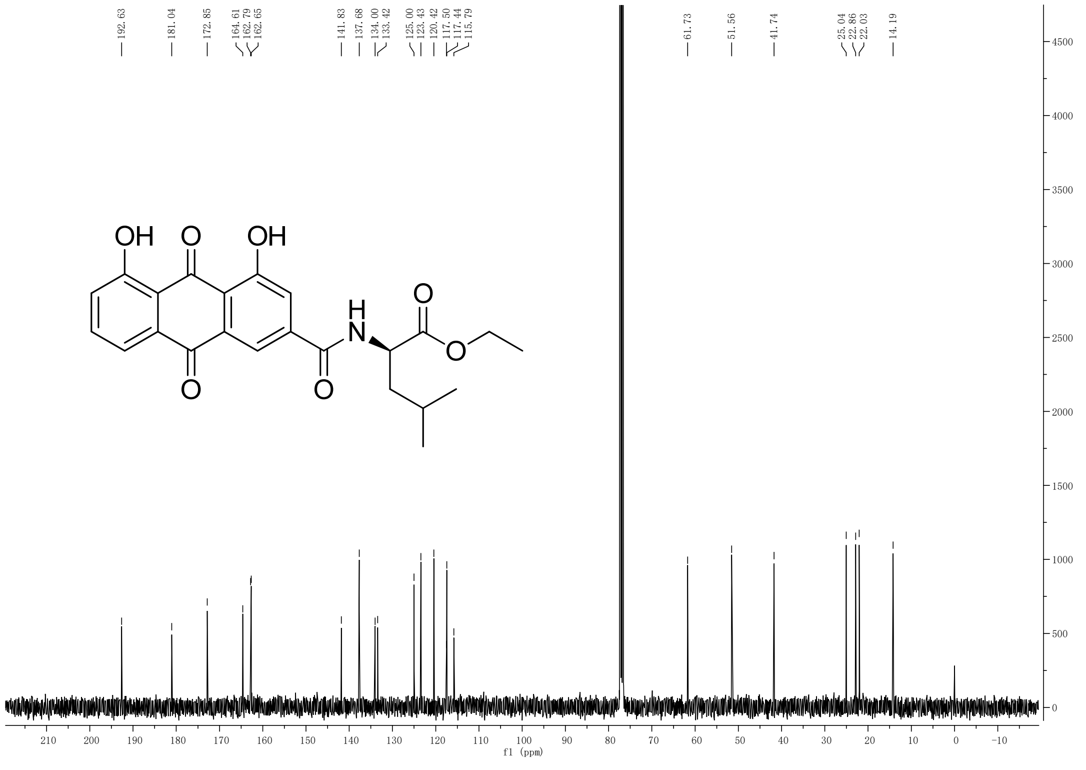

Figure S14. ^{13}C-NMR Spectrum of compound **3e**

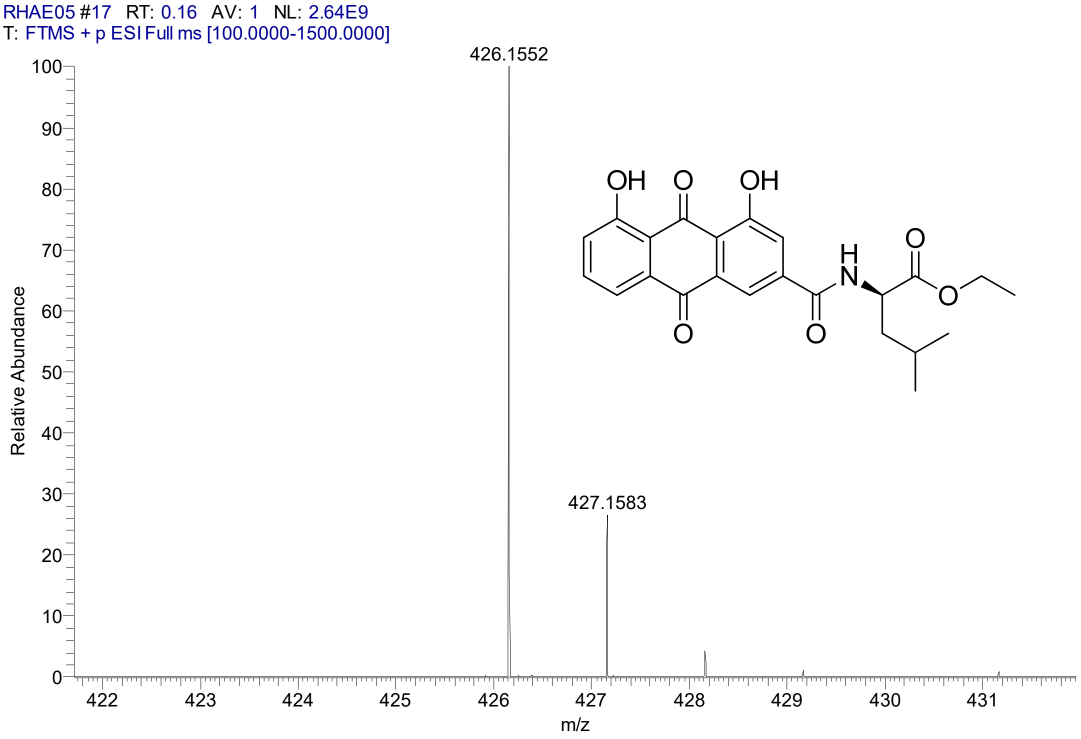

Figure S15. HRMS Spectrum of compound **3e**

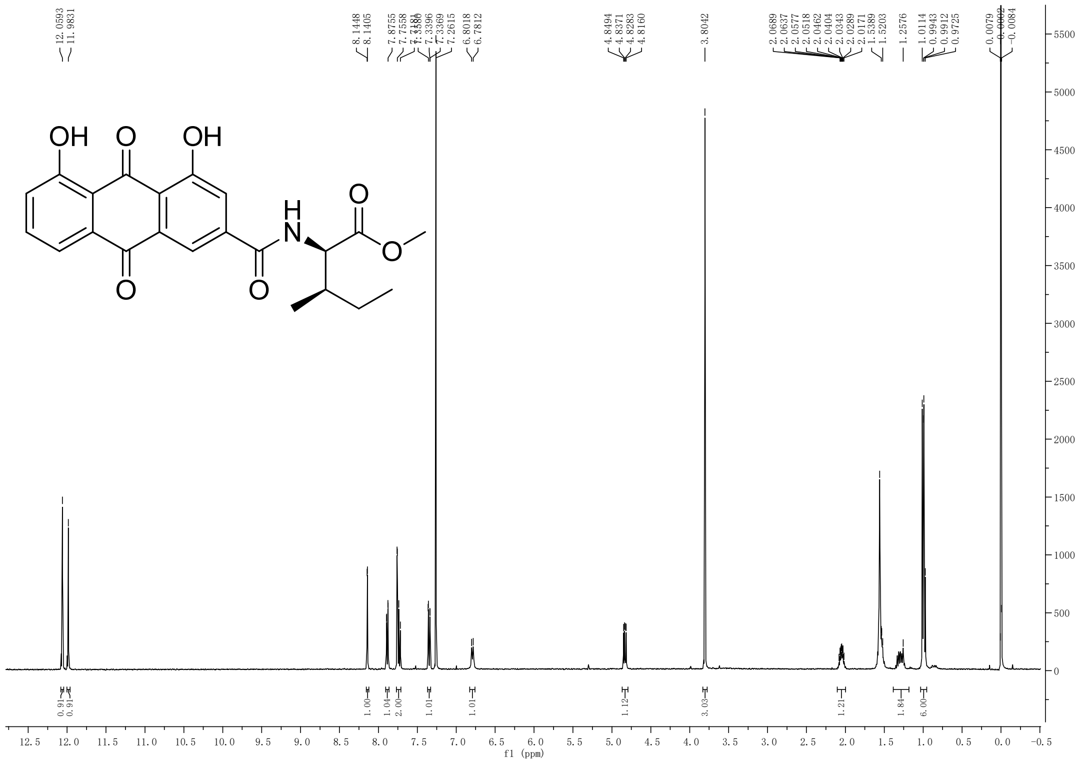

Figure S16 ^{1}H-NMR Spectrum of compound **3f**

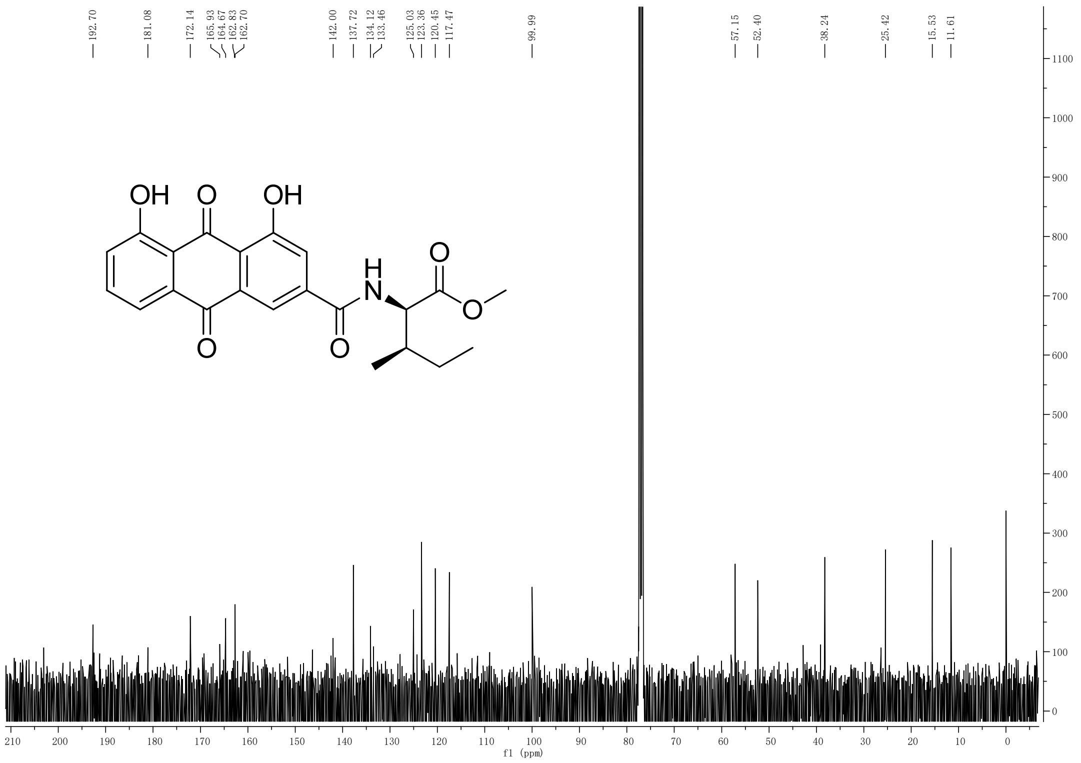

Figure S17. ^{13}C-NMR Spectrum of compound **3f**

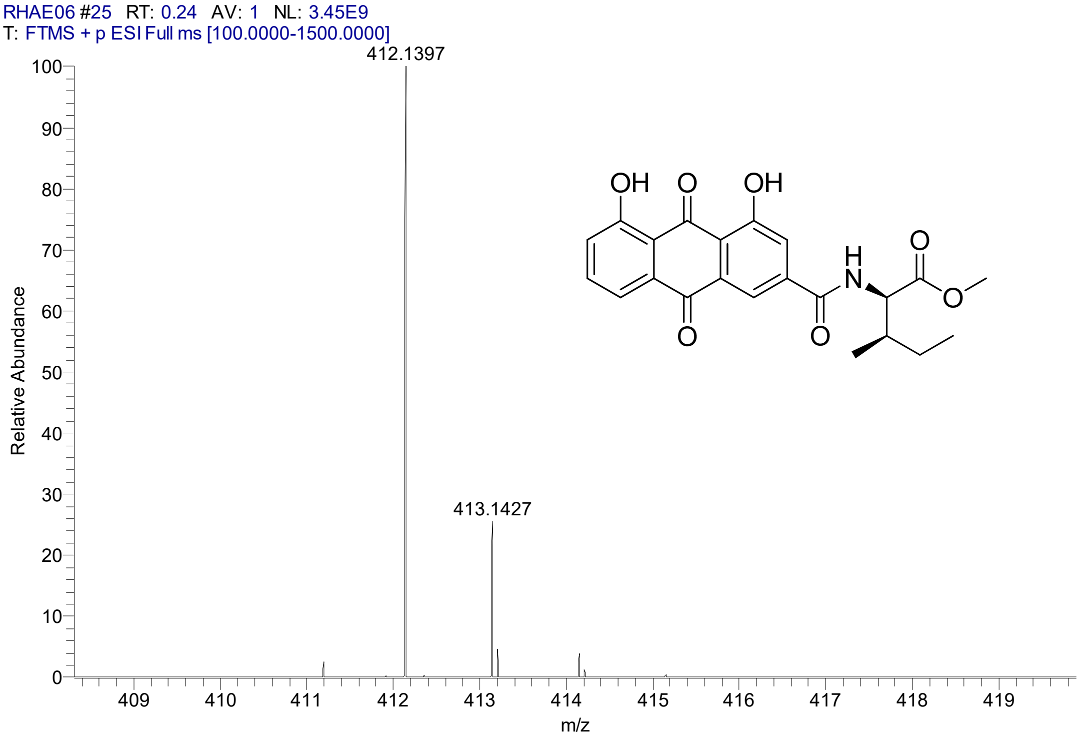

Figure S18. HRMS Spectrum of compound **3f**

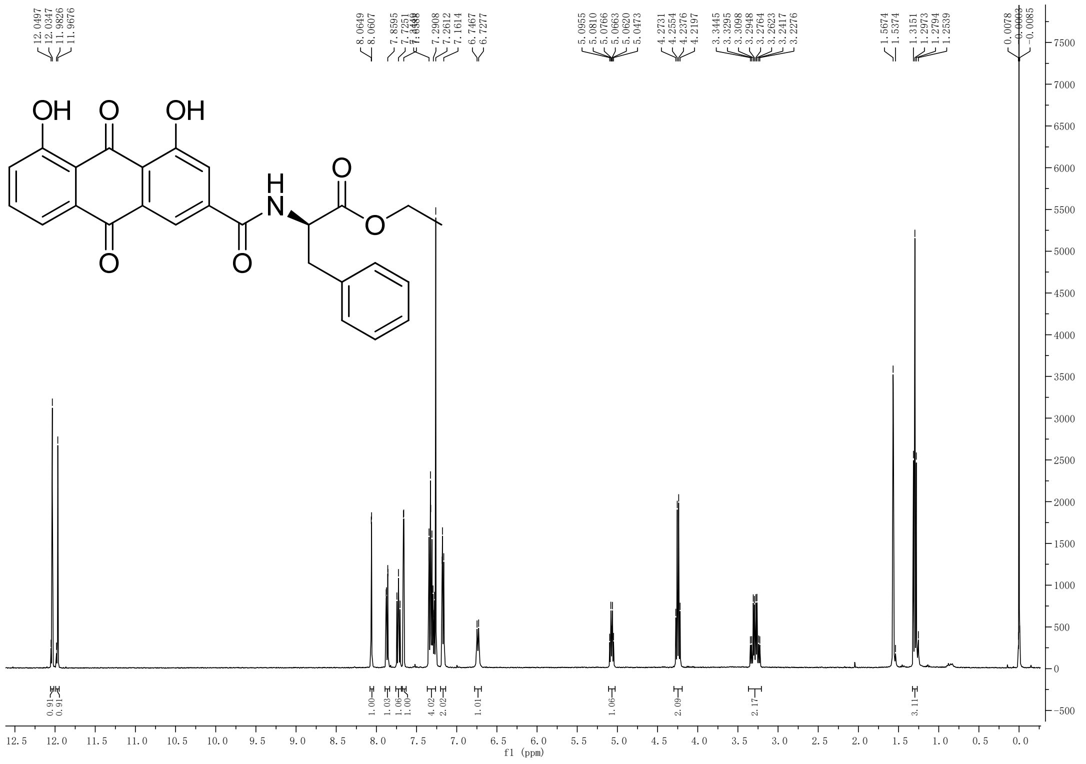

Figure S19. ^{1}H-NMR Spectrum of compound **3g**

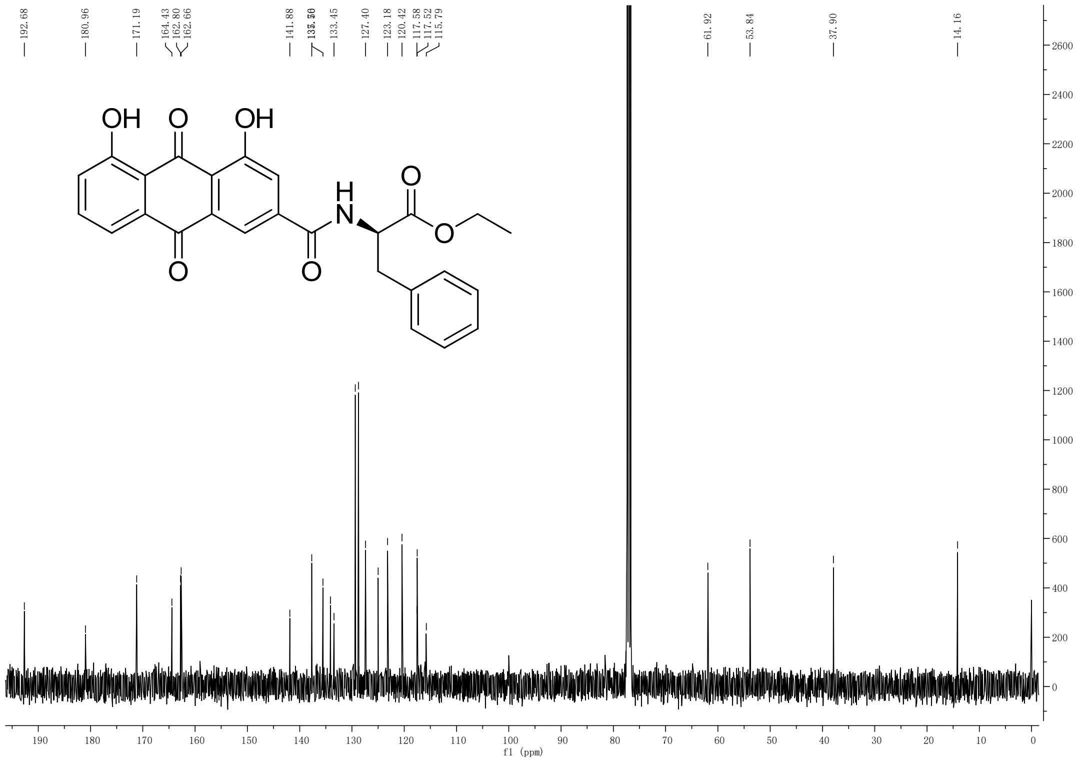

Figure S20. ^{13}C-NMR Spectrum of compound **3g**

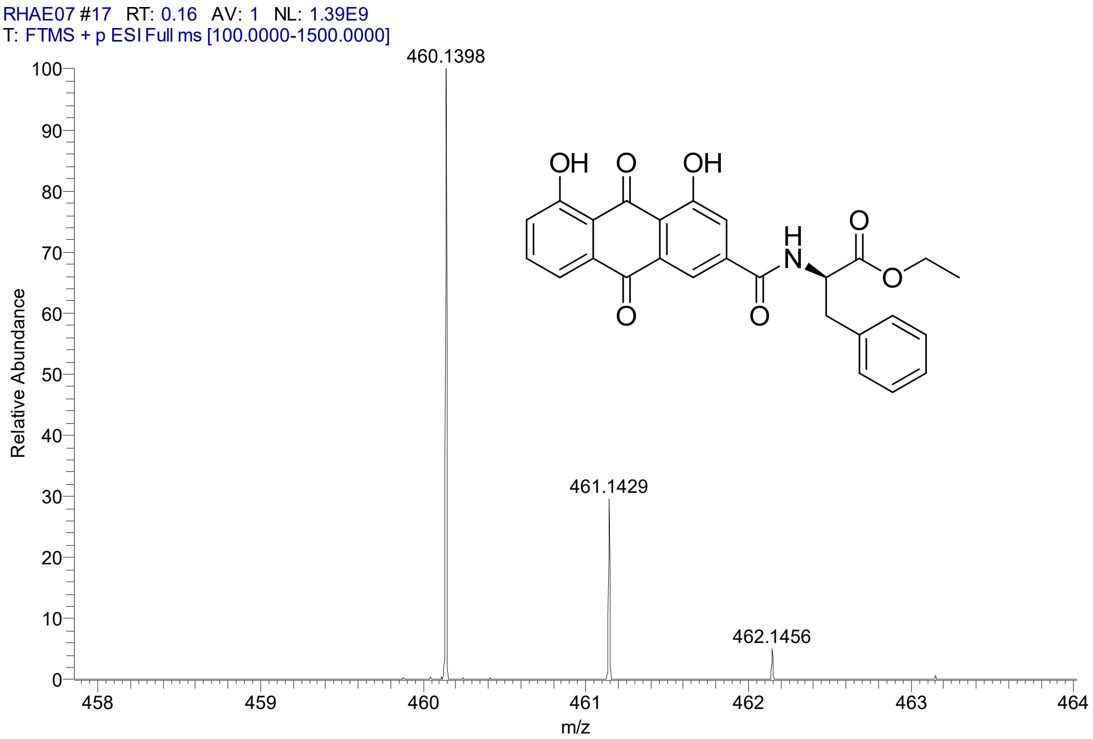

Figure S21. HRMS Spectrum of compound **3g**

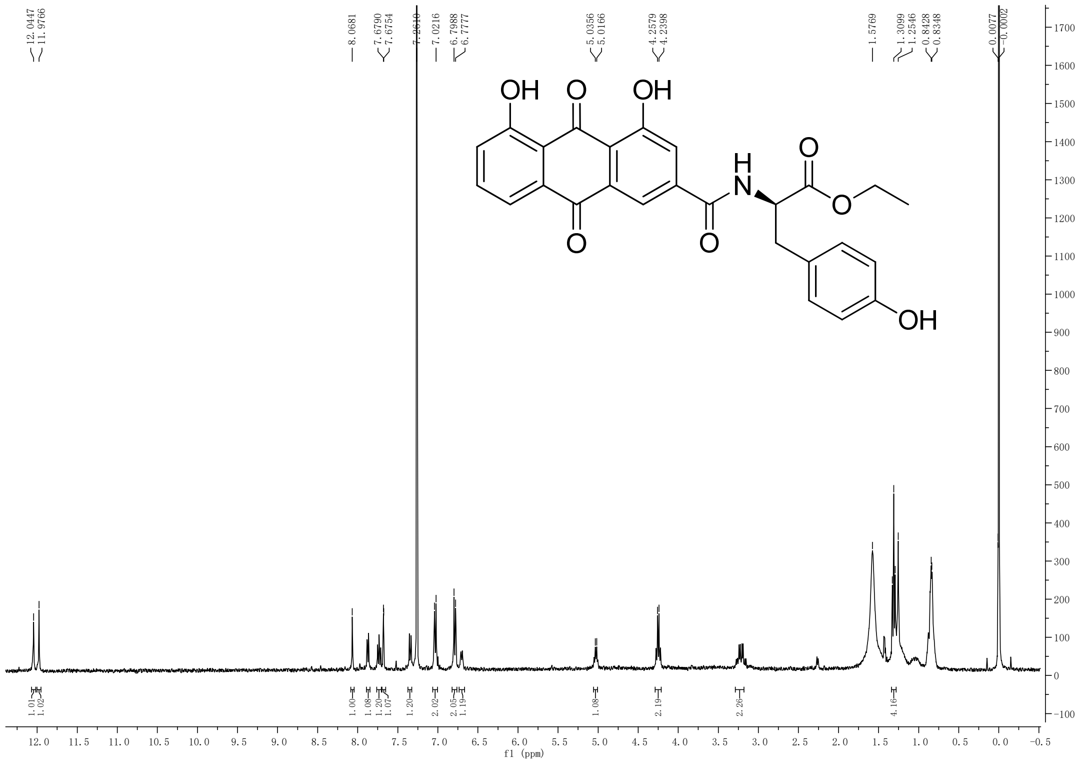

Figure S22. ^{1}H-NMR Spectrum of compound **3h**

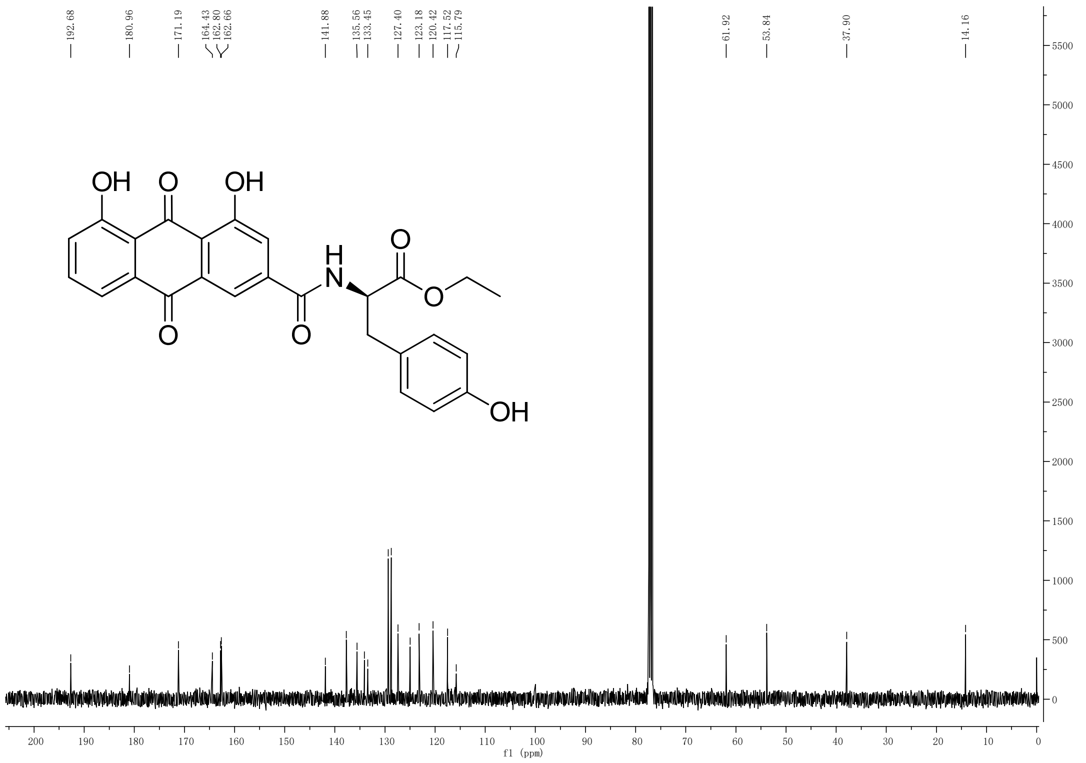

Figure S23. ^{13}C-NMR Spectrum of compound **3h**

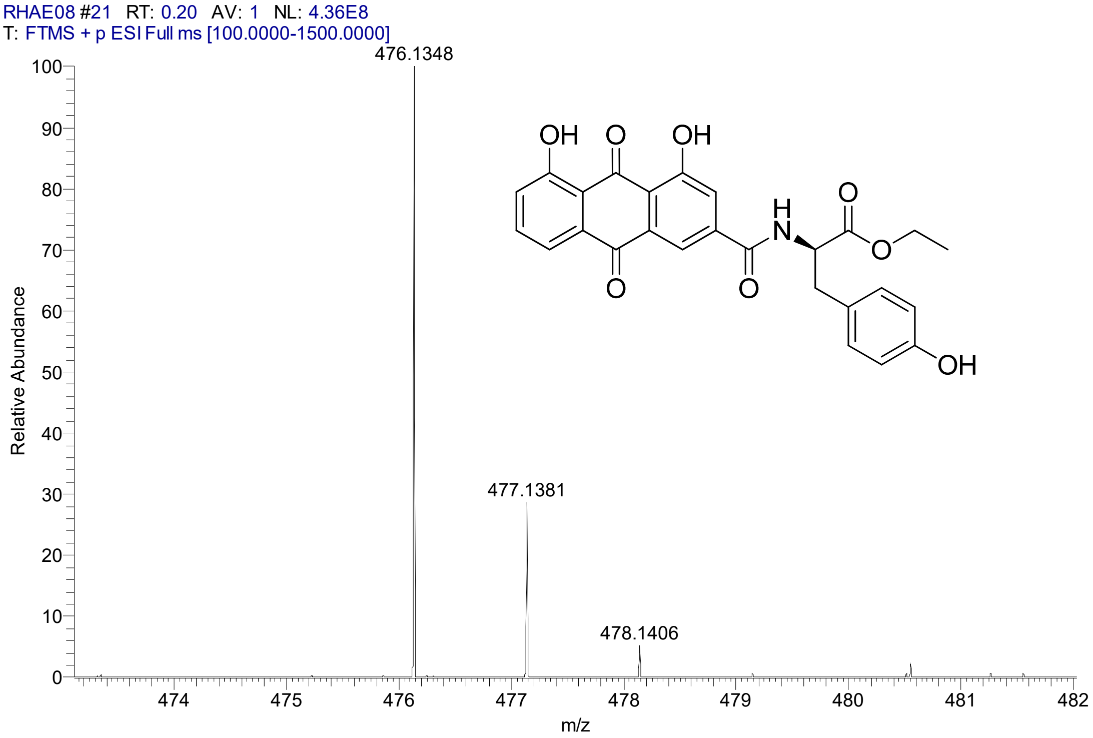

Figure S24. HRMS Spectrum of compound **3h**

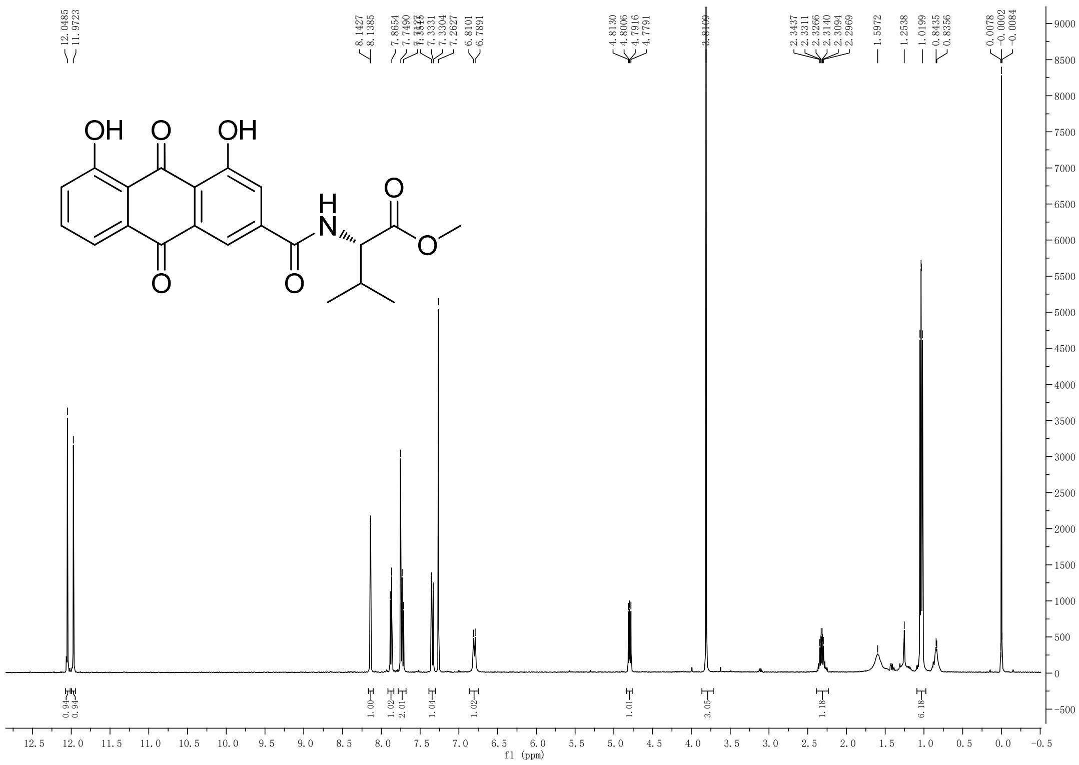

Figure S25. ^{1}H-NMR Spectrum of compound **3i**

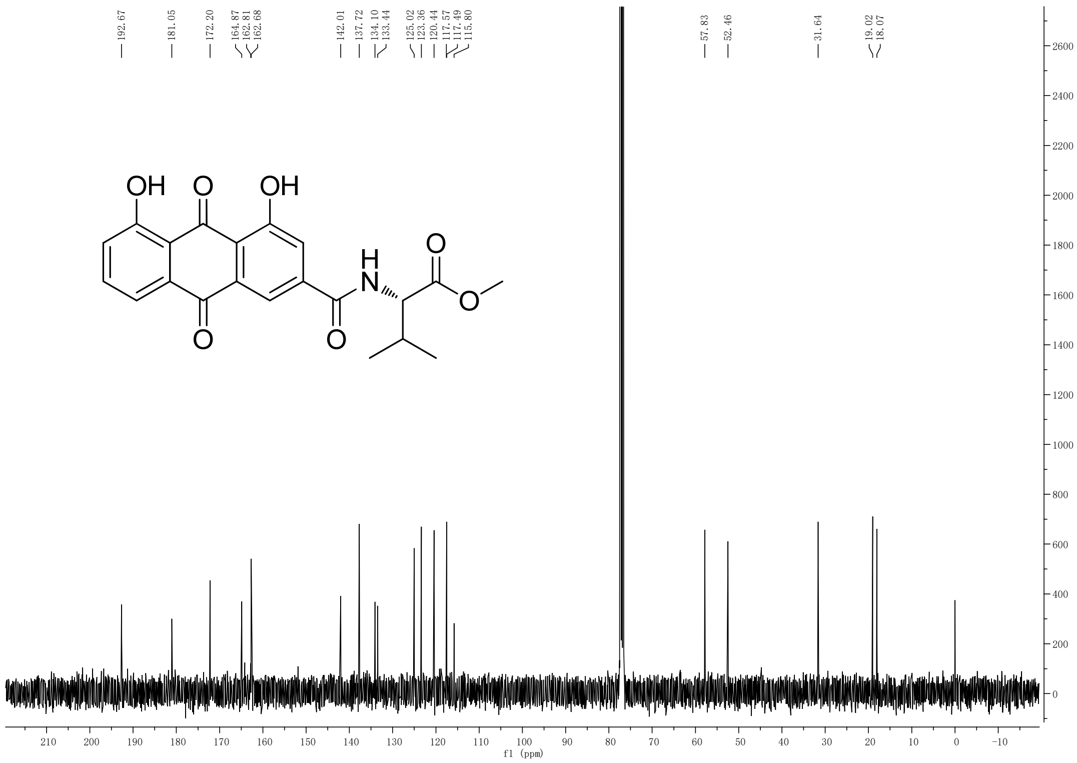

Figure S26. ^{13}C-NMR Spectrum of compound **3i**

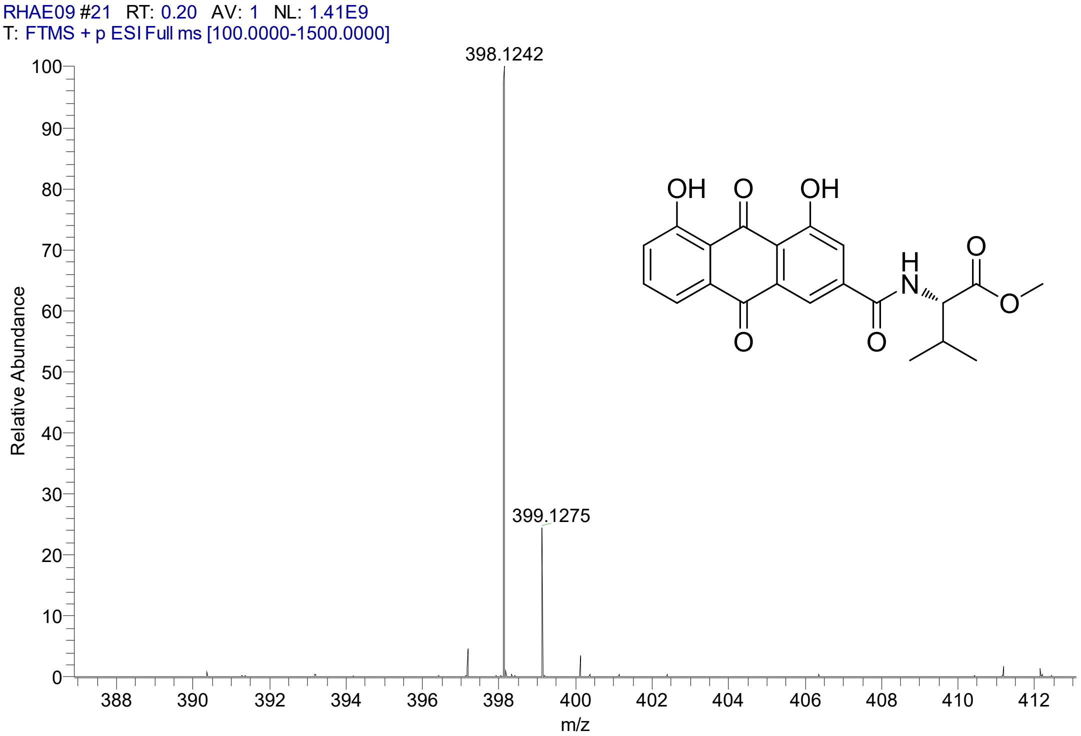

Figure S27. HRMS Spectrum of compound **3i**

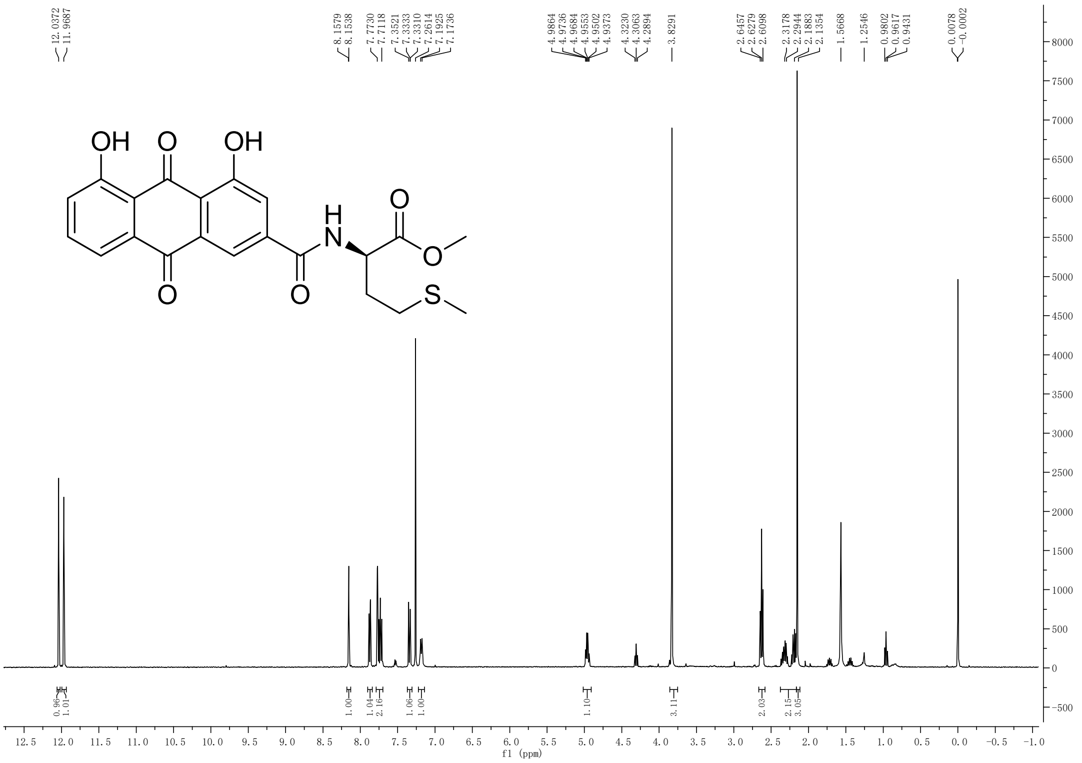

Figure S28. ^{1}H-NMR Spectrum of compound **3j**

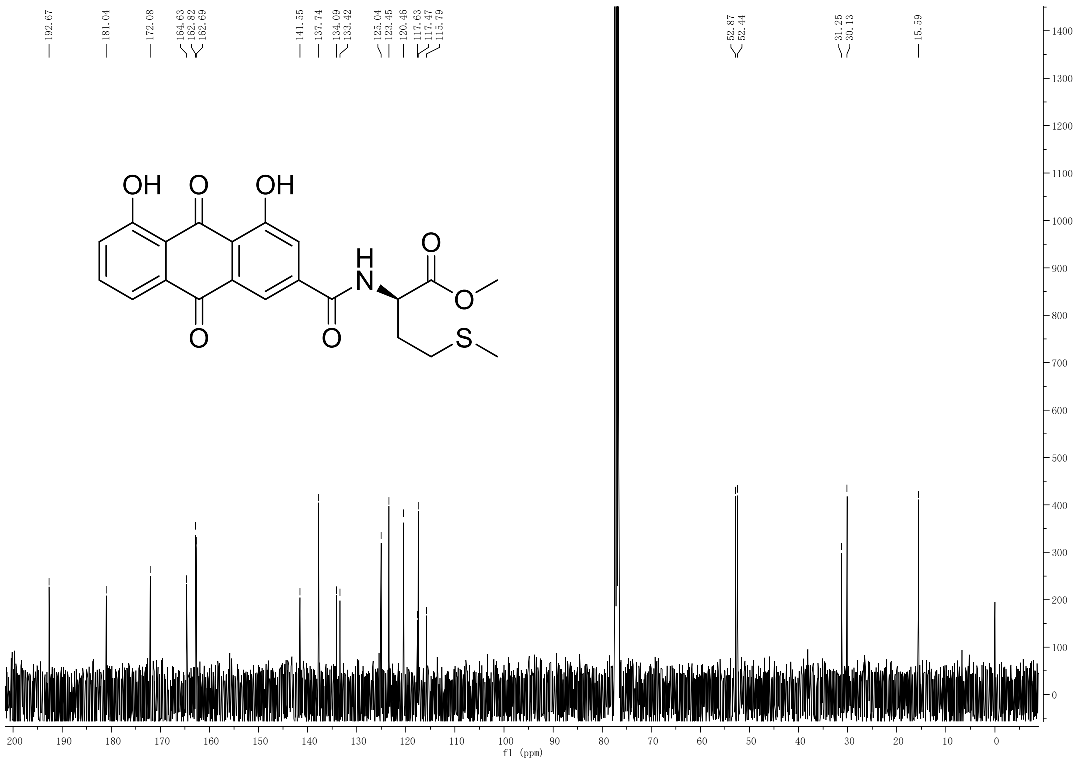

Figure S29. ^{13}C-NMR Spectrum of compound **3j**

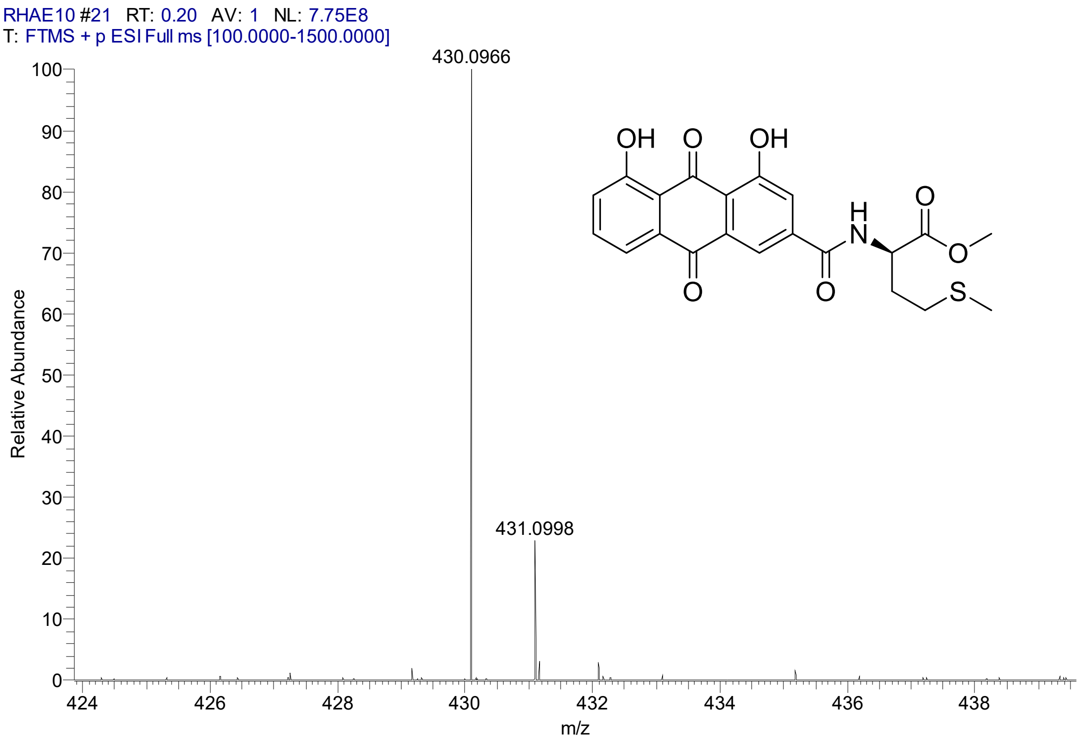

Figure S30. HRMS Spectrum of compound **3j**

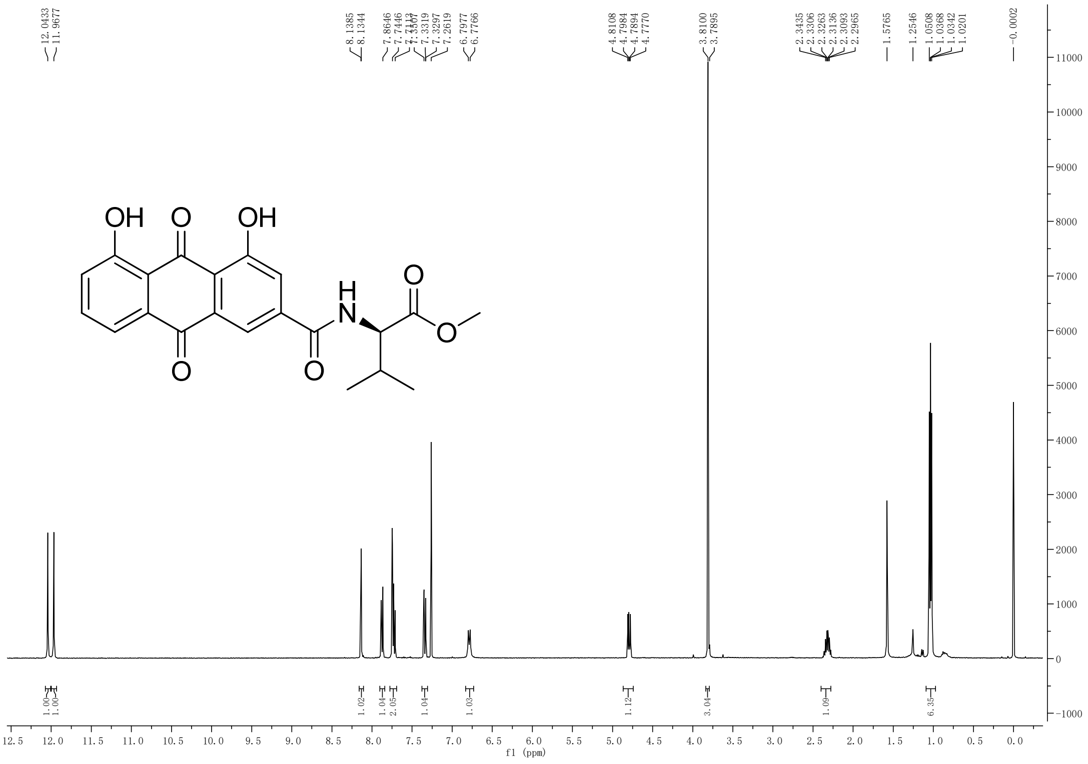

Figure S31. ^{1}H-NMR Spectrum of compound **3k**

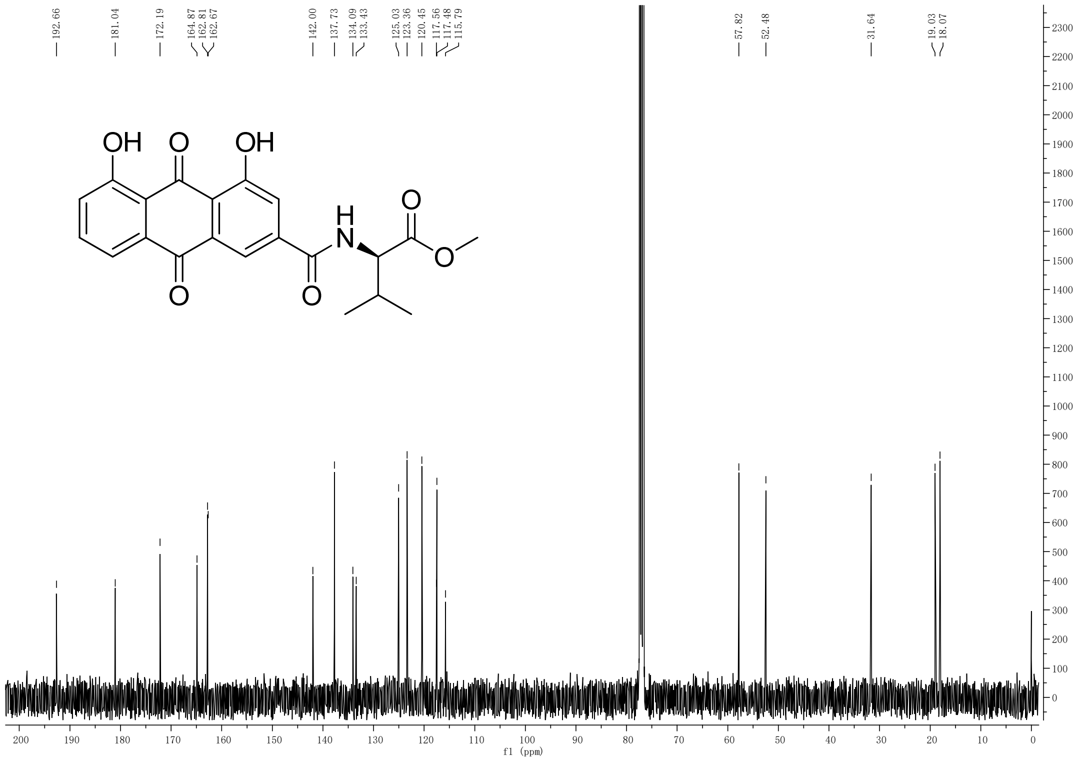

Figure S32. ^{13}C-NMR Spectrum of compound **3k**

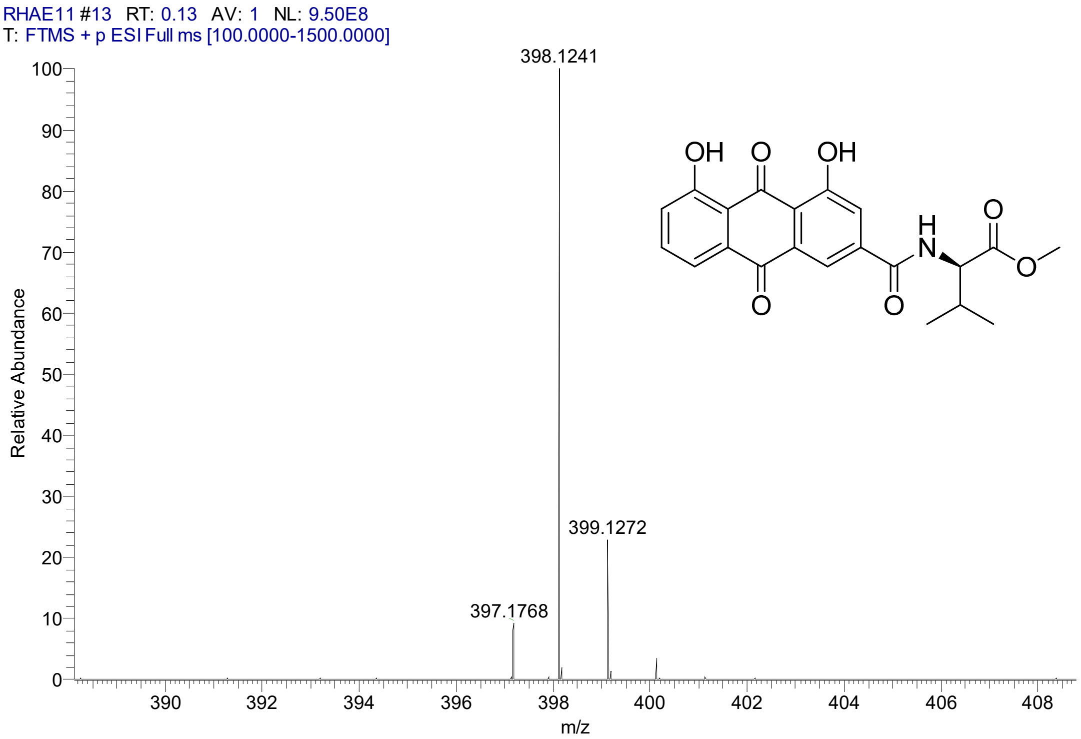

Figure S33. HRMS Spectrum of compound **3k**

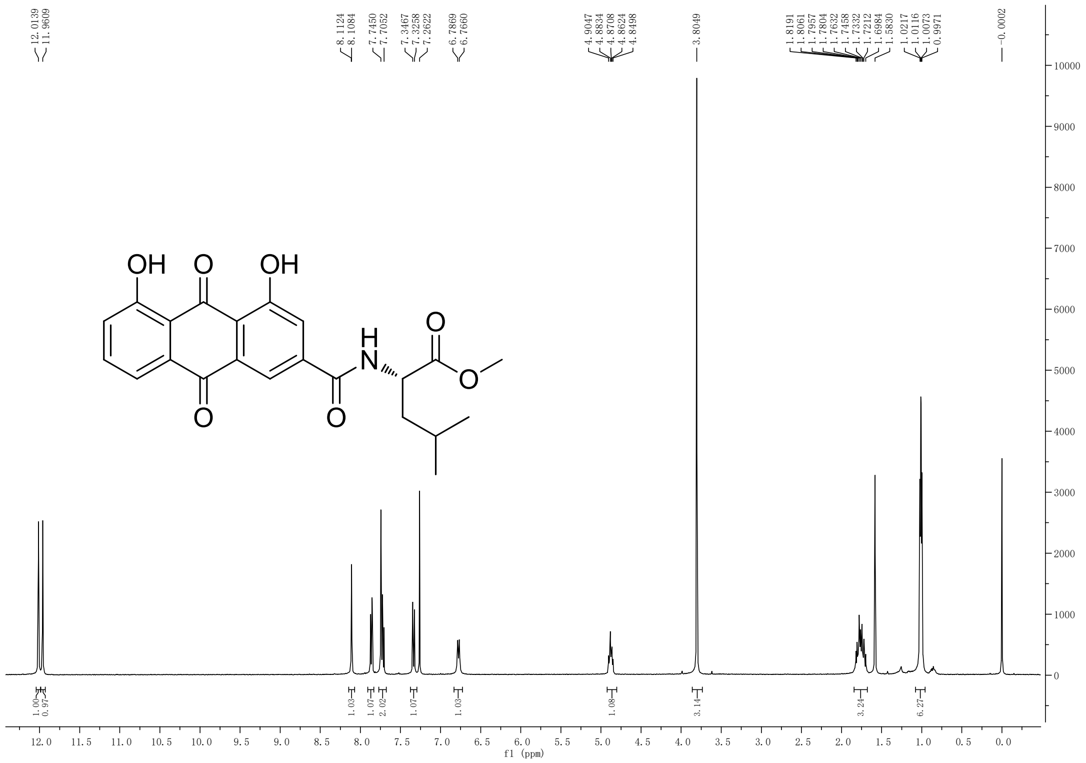

Figure S34. ^{1}H-NMR Spectrum of compound **3l**

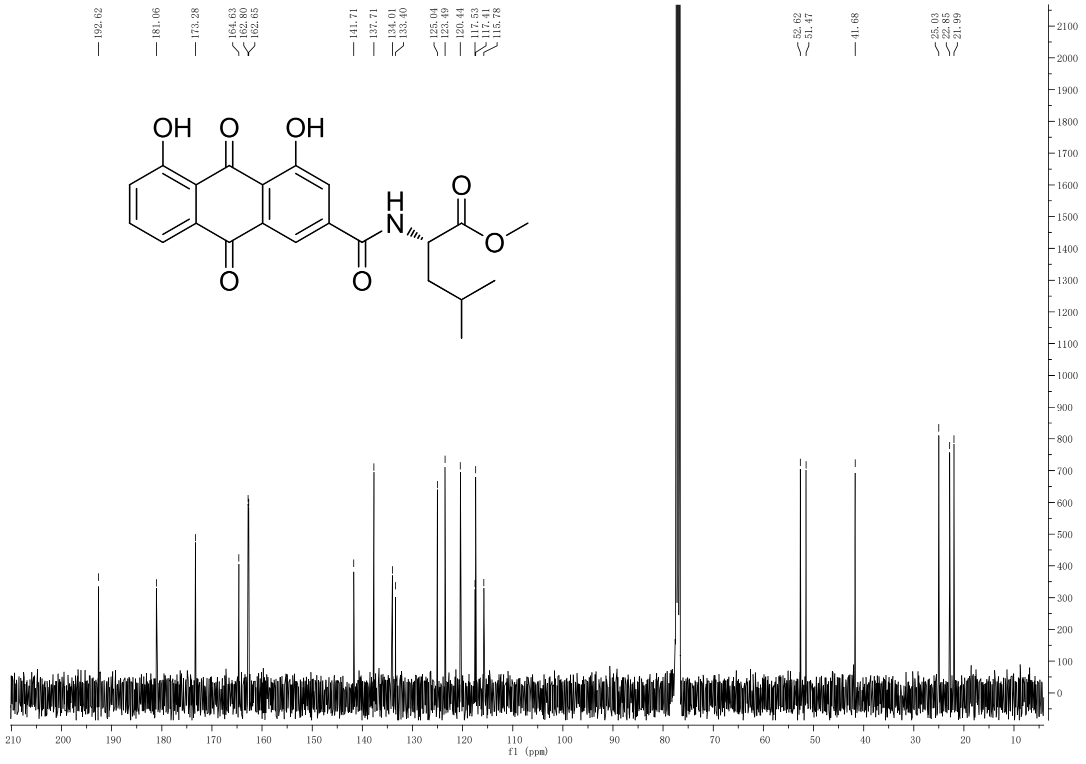

Figure S35. ^{13}C-NMR Spectrum of compound **3l**

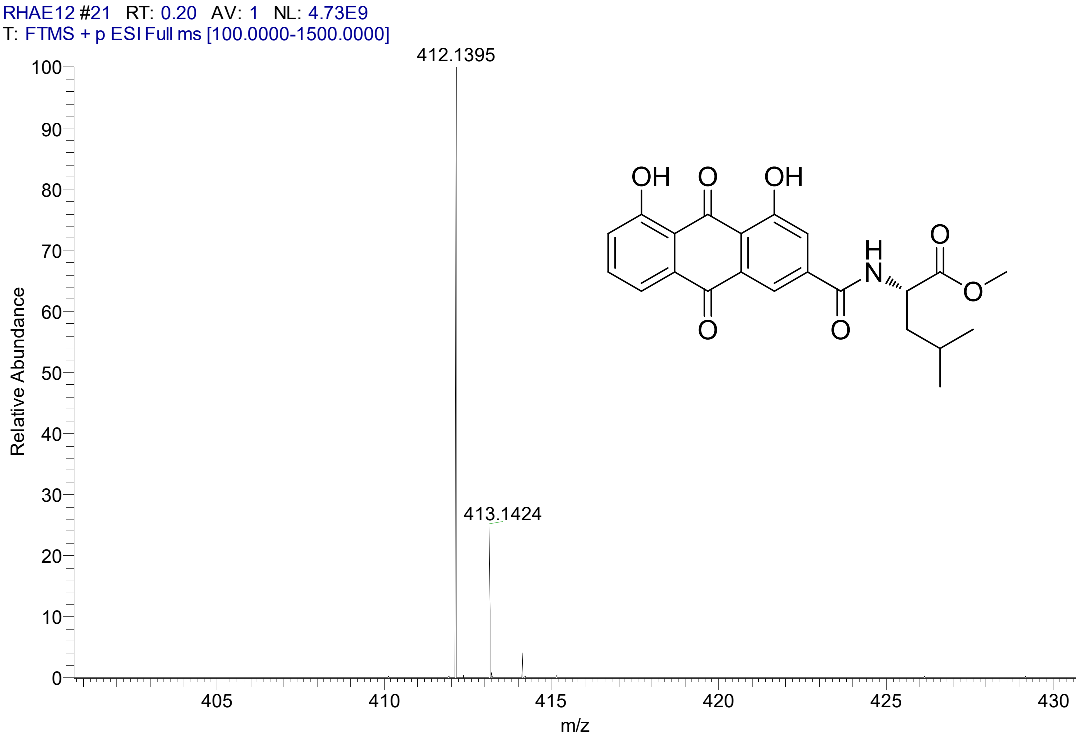

Figure S36. HRMS Spectrum of compound **3l**

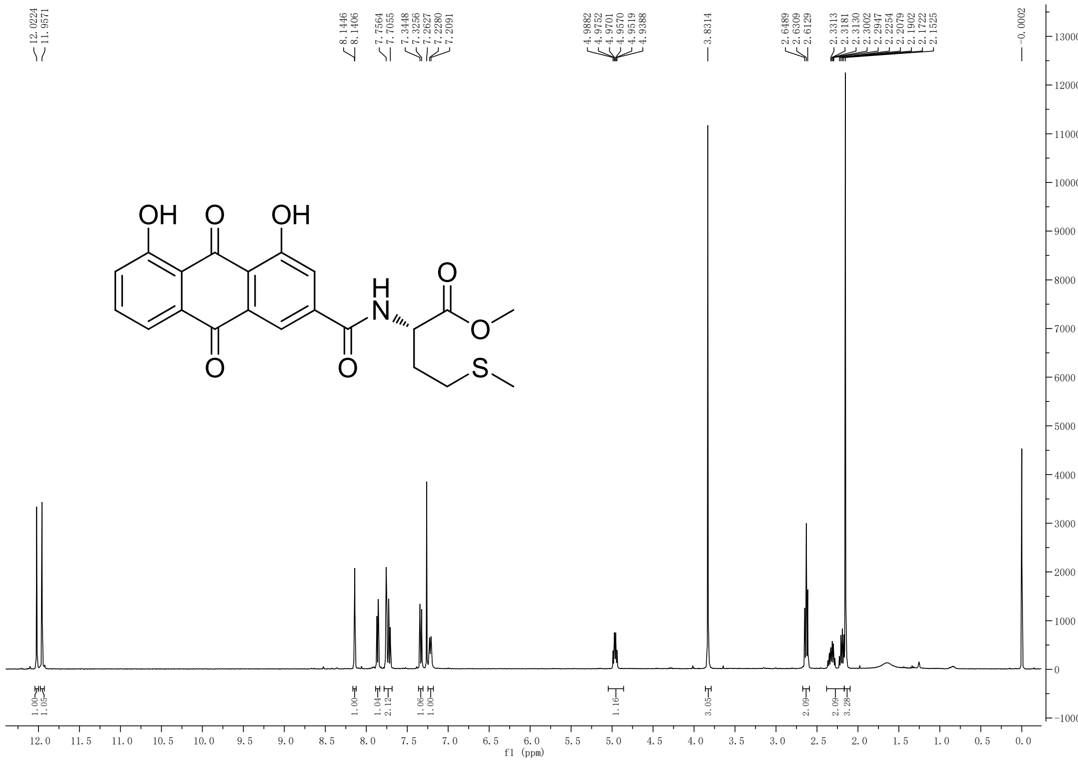

Figure S37. ^{1}H-NMR Spectrum of compound **3m**

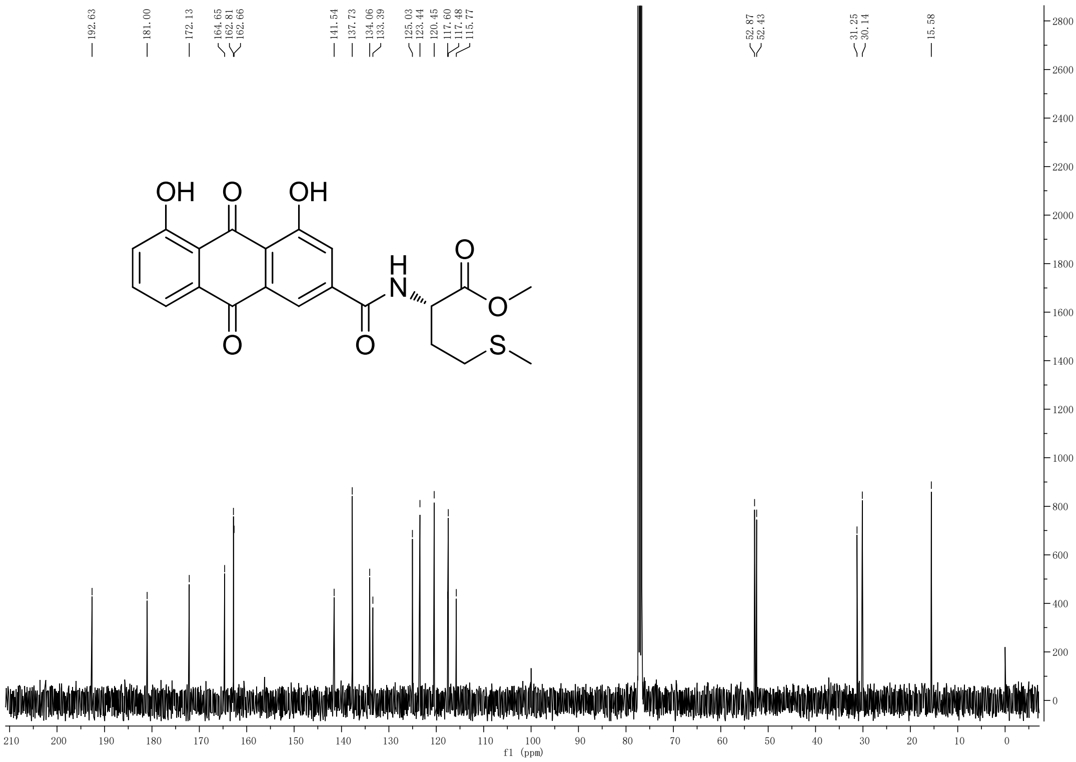

Figure S38. ^{13}C-NMR Spectrum of compound **3m**

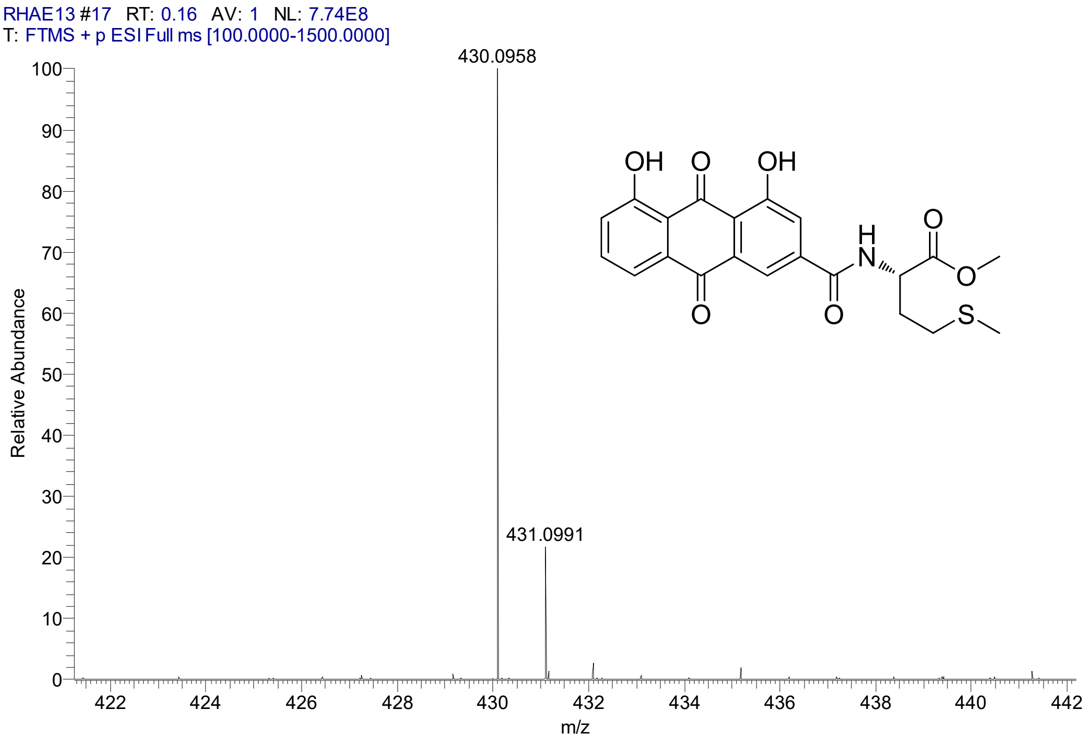

Figure S39. HRMS Spectrum of compound **3m**

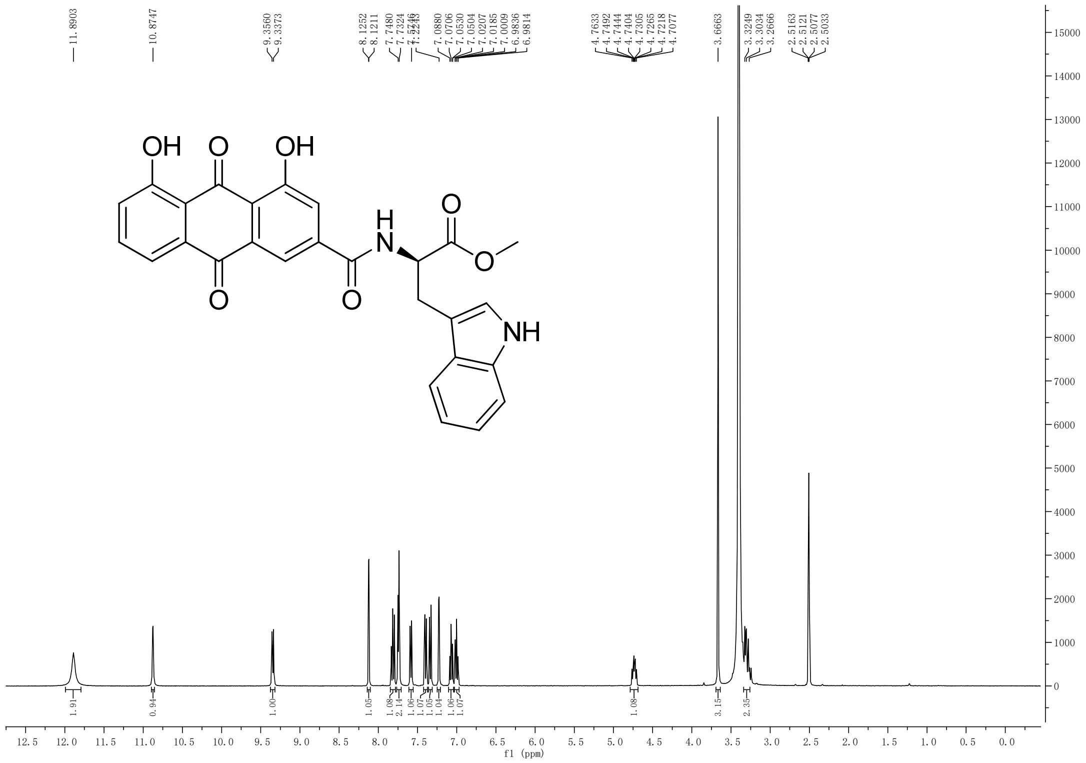

Figure S40. ^{1}H-NMR Spectrum of compound **3n**

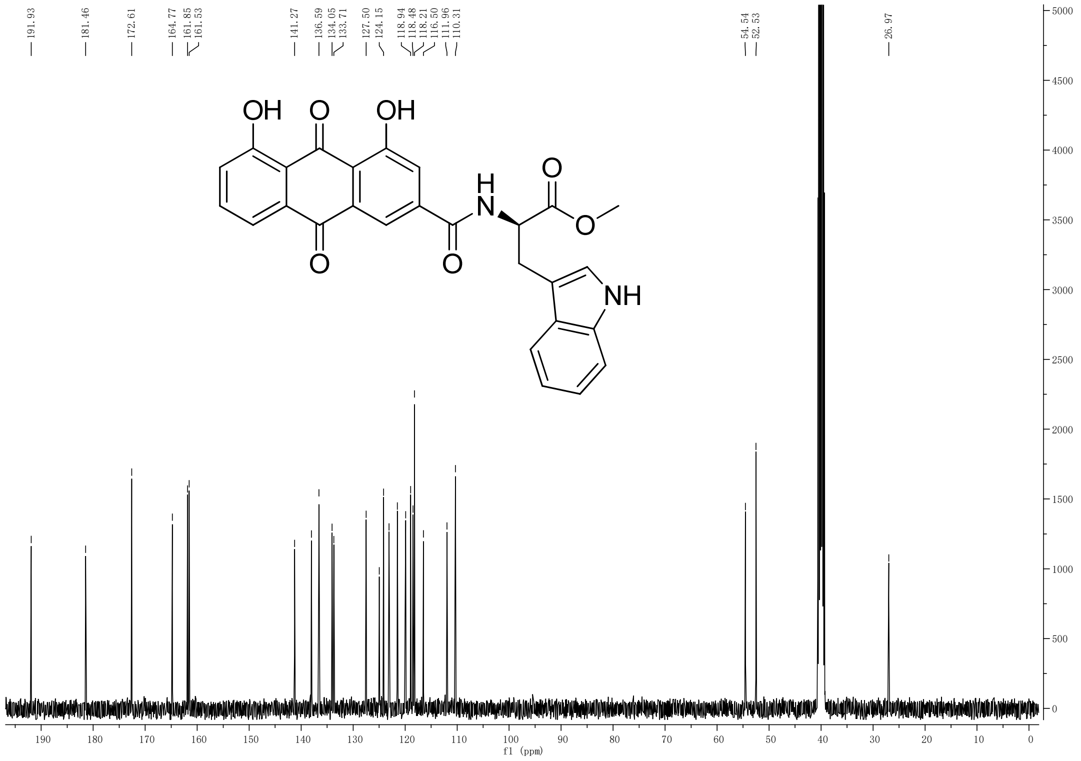

Figure S41. ^{13}C-NMR Spectrum of compound **3n**

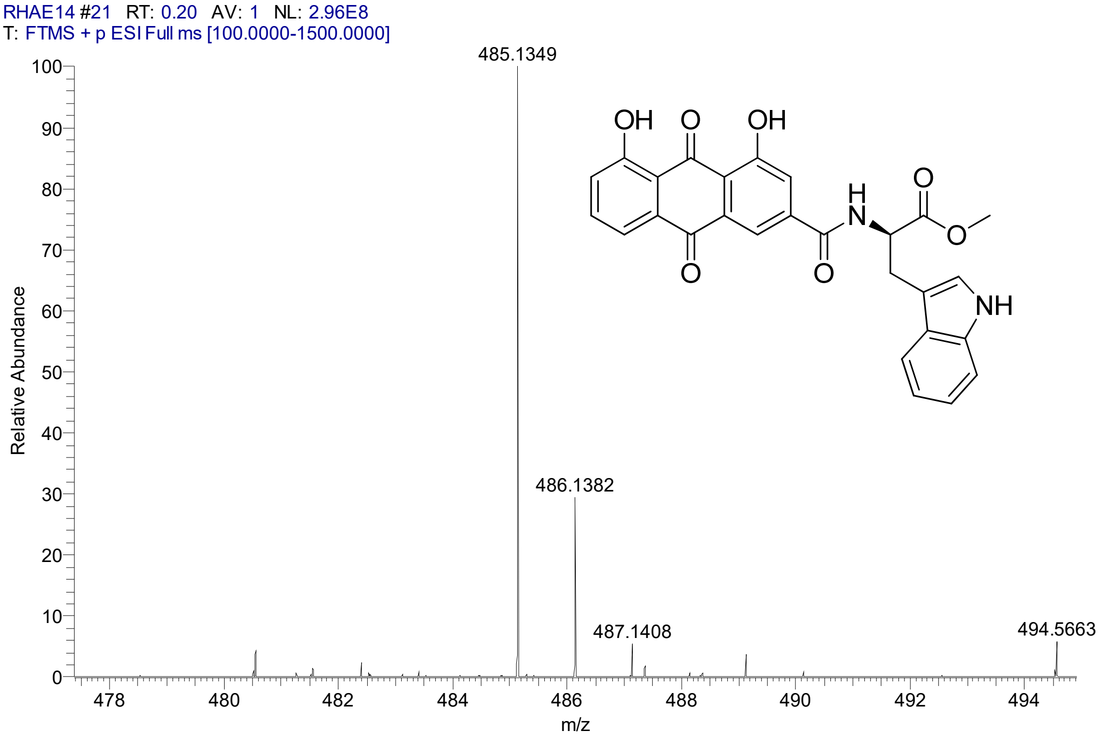

Figure S42. HRMS Spectrum of compound **3n**

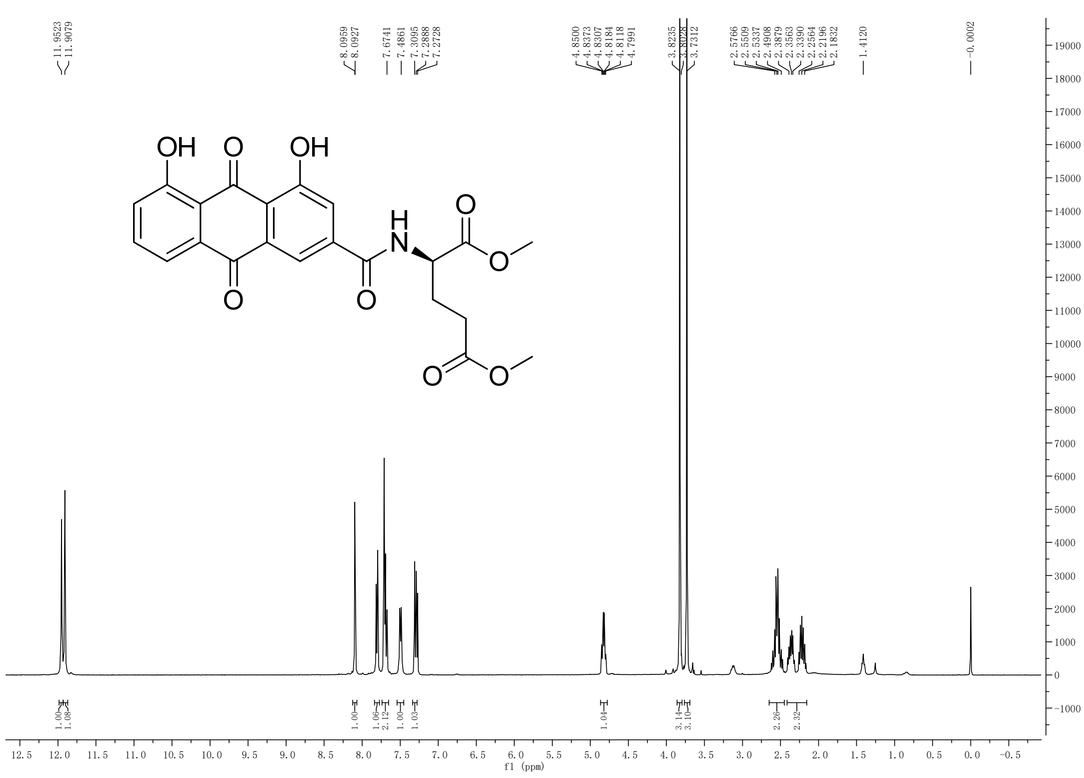

Figure S43. ^{1}H-NMR Spectrum of compound **3o**

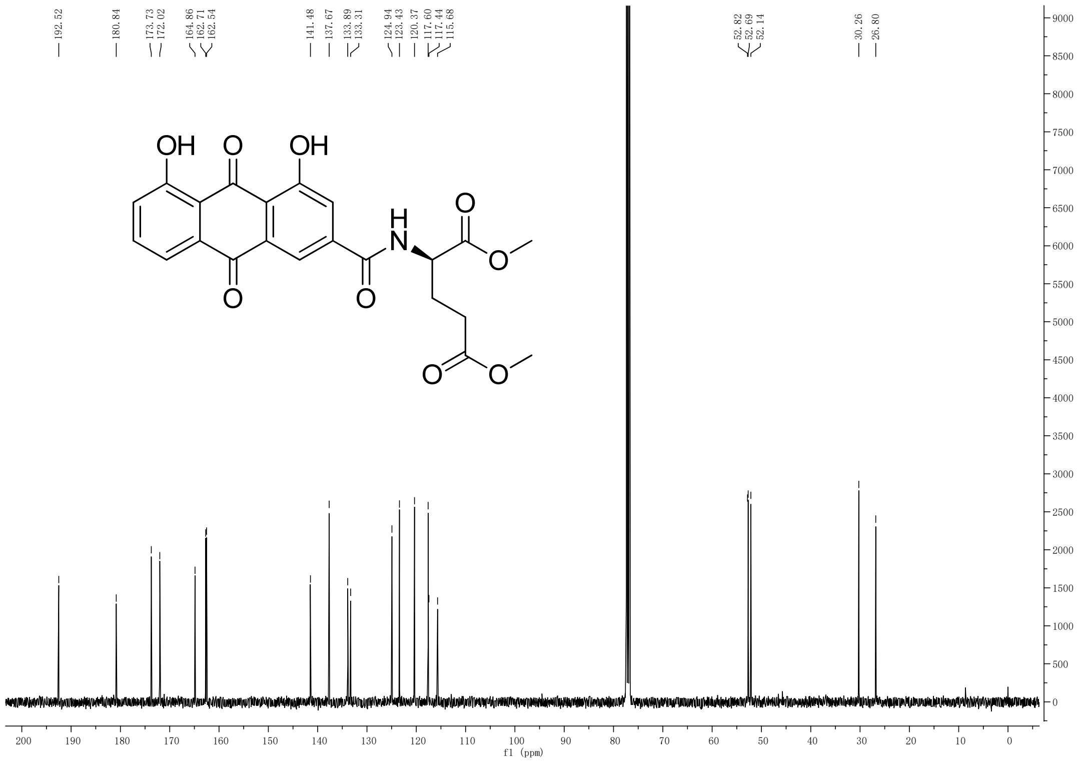

Figure S44. ^{13}C-NMR Spectrum of compound **3o**

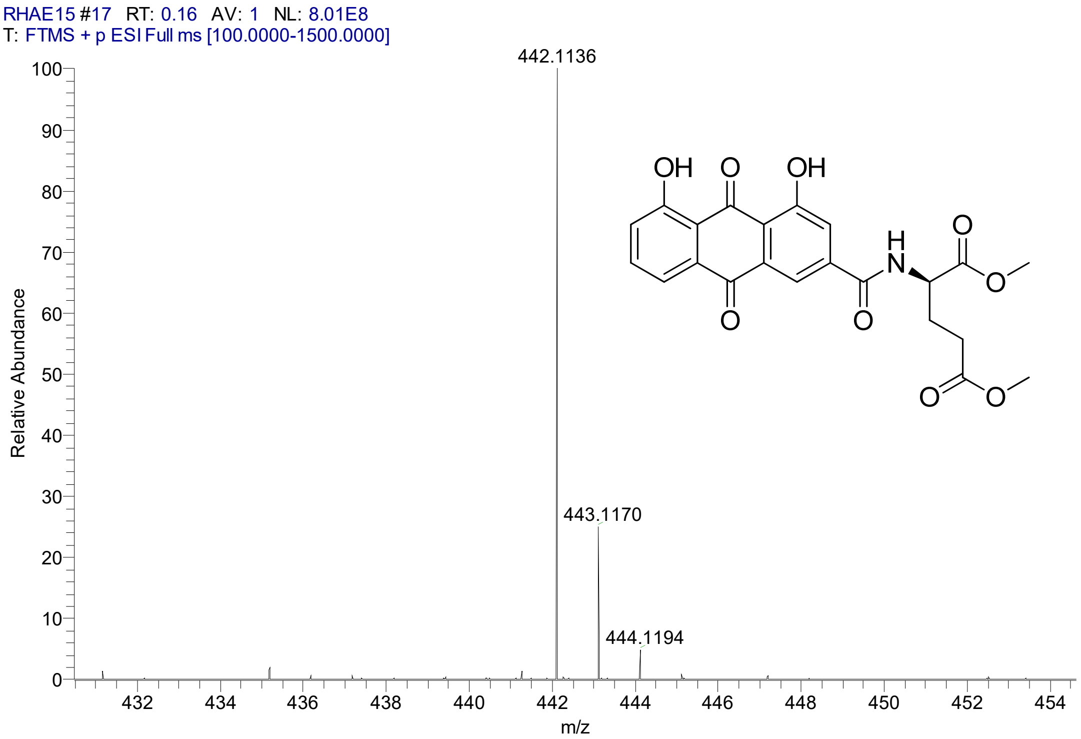

Figure S45. HRMS Spectrum of compound **3o**

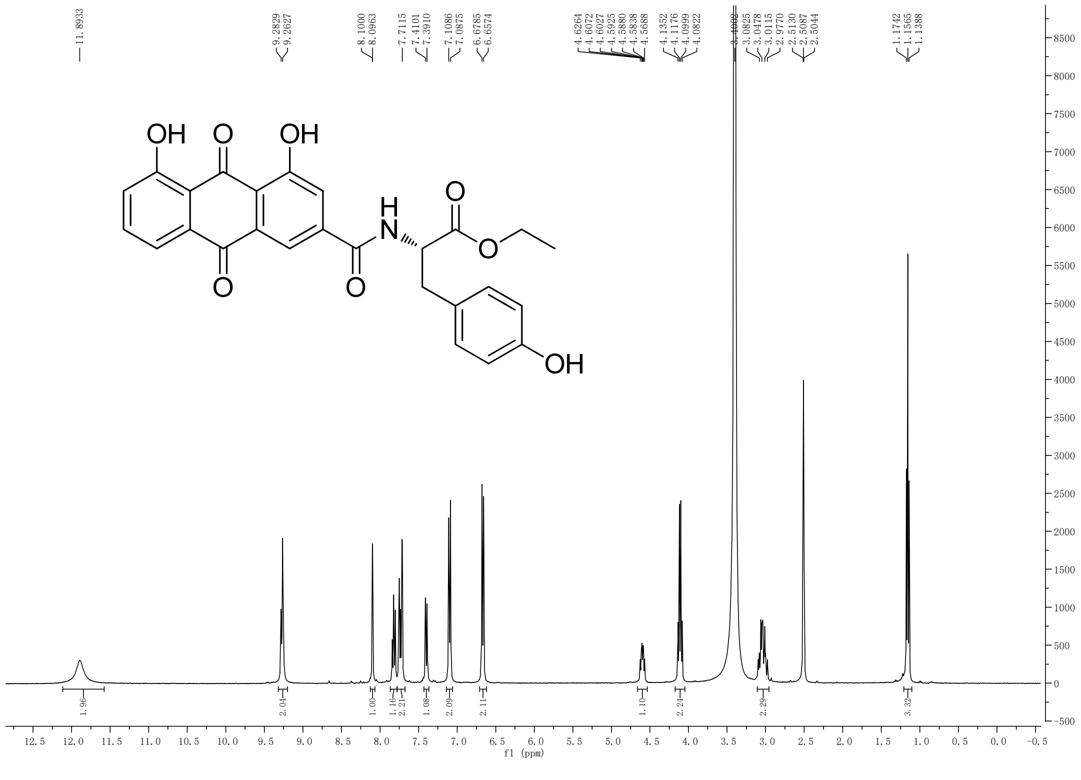

Figure S46. ^{1}H-NMR Spectrum of compound **3p**

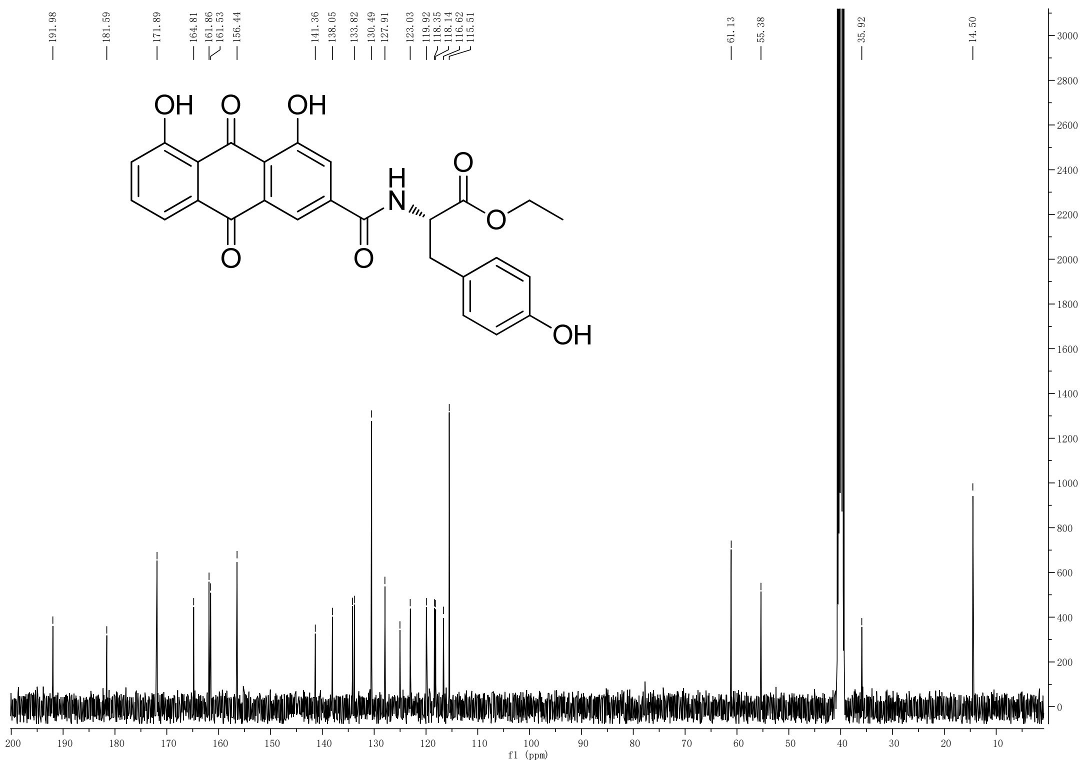

Figure S47. ^{13}C-NMR Spectrum of compound **3p**

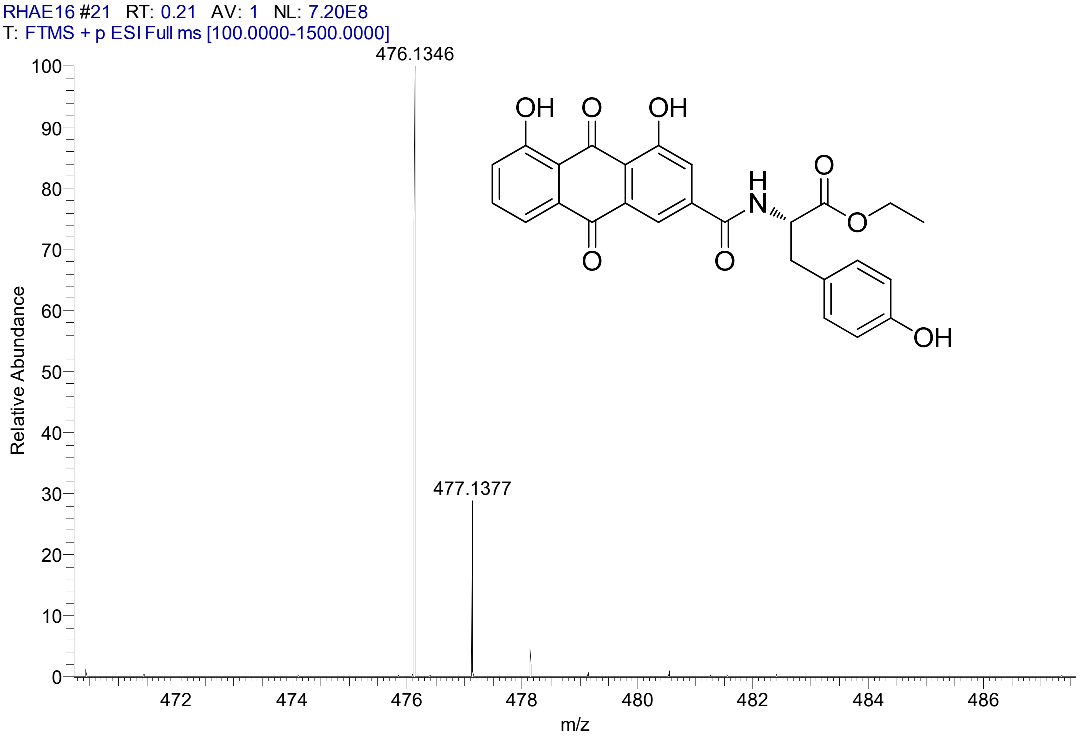

Figure S48. HRMS Spectrum of compound **3p**

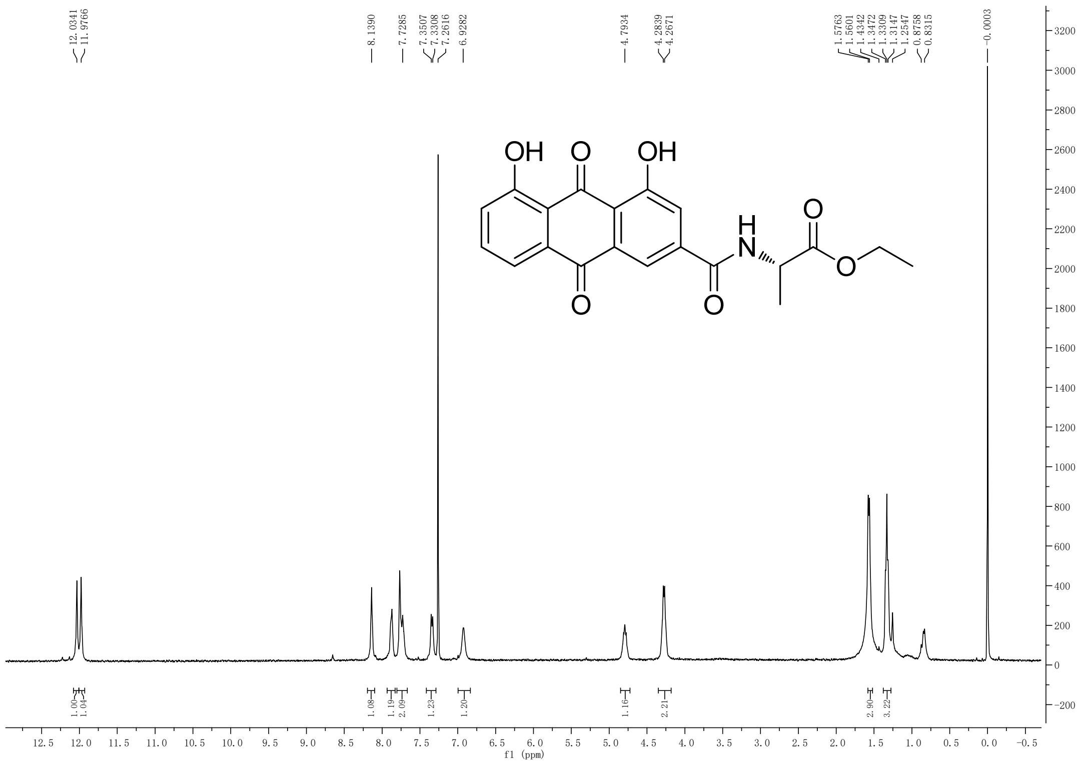

Figure S49. ^{1}H-NMR Spectrum of compound **3q**

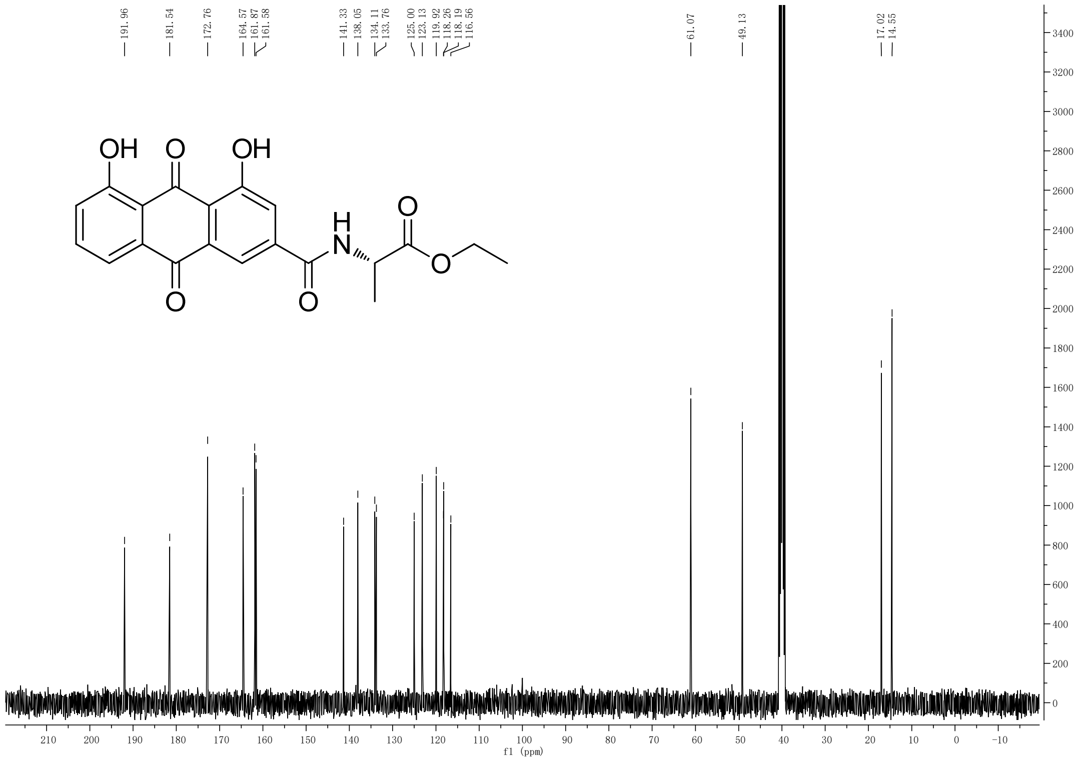

Figure S50. ^{13}C-NMR Spectrum of compound **3q**

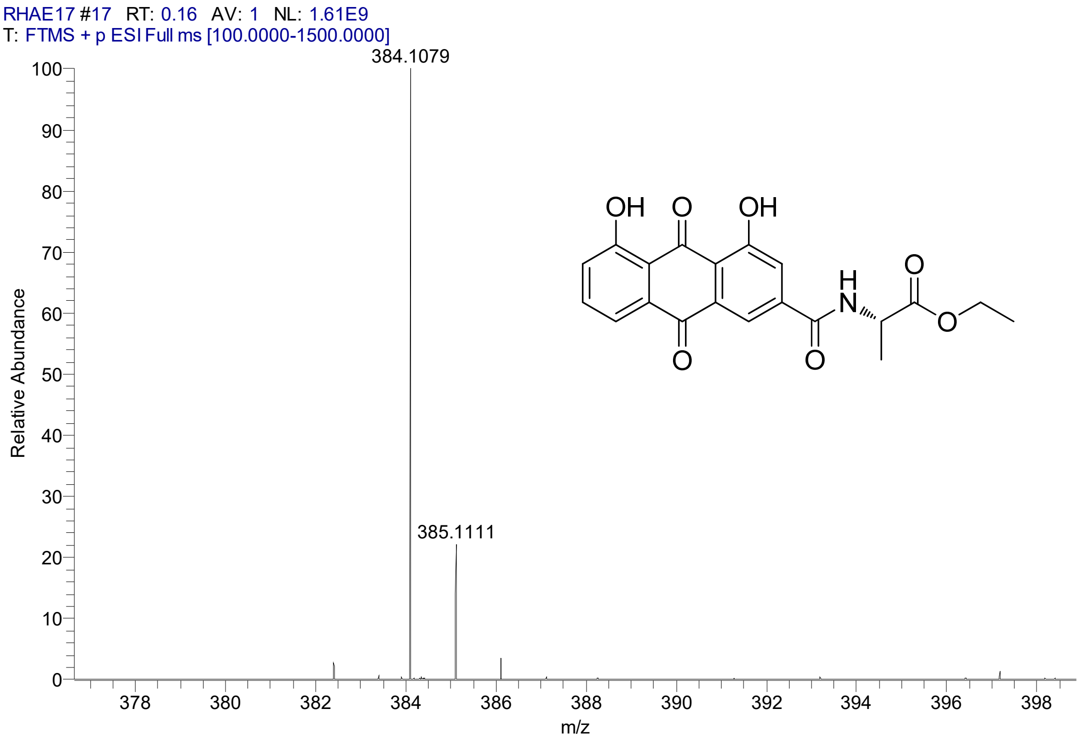

Figure S51. HRMS Spectrum of compound **3q**

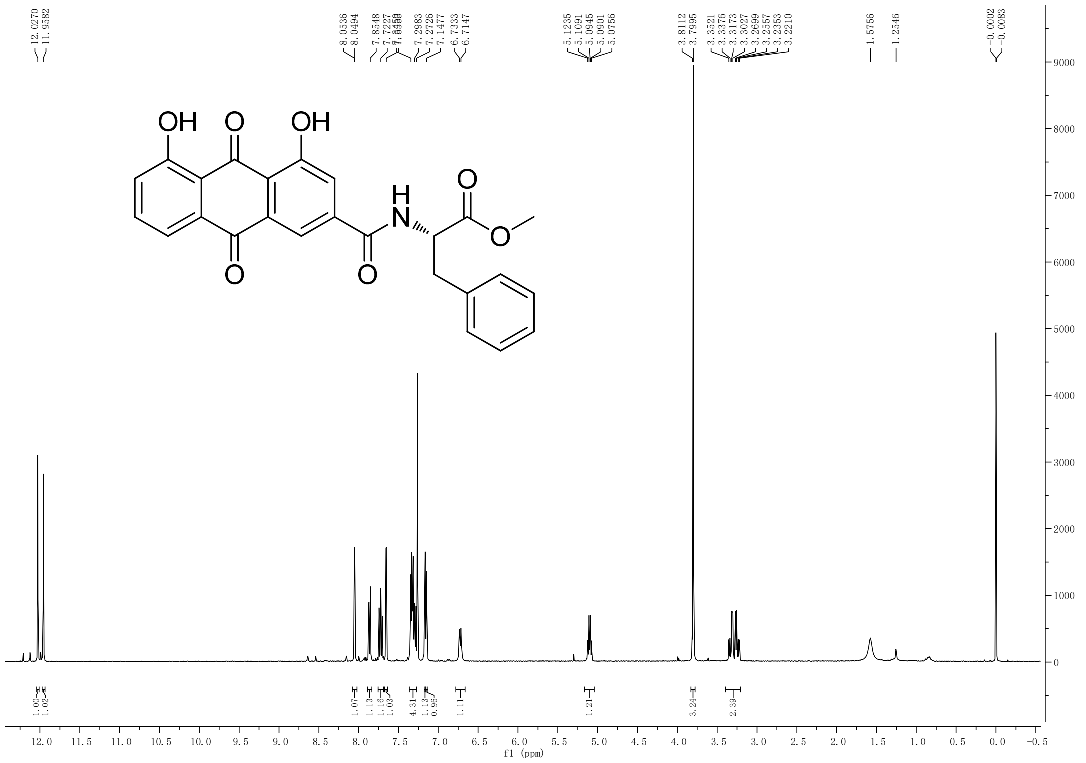

Figure S52. ^{1}H-NMR Spectrum of compound **3r**

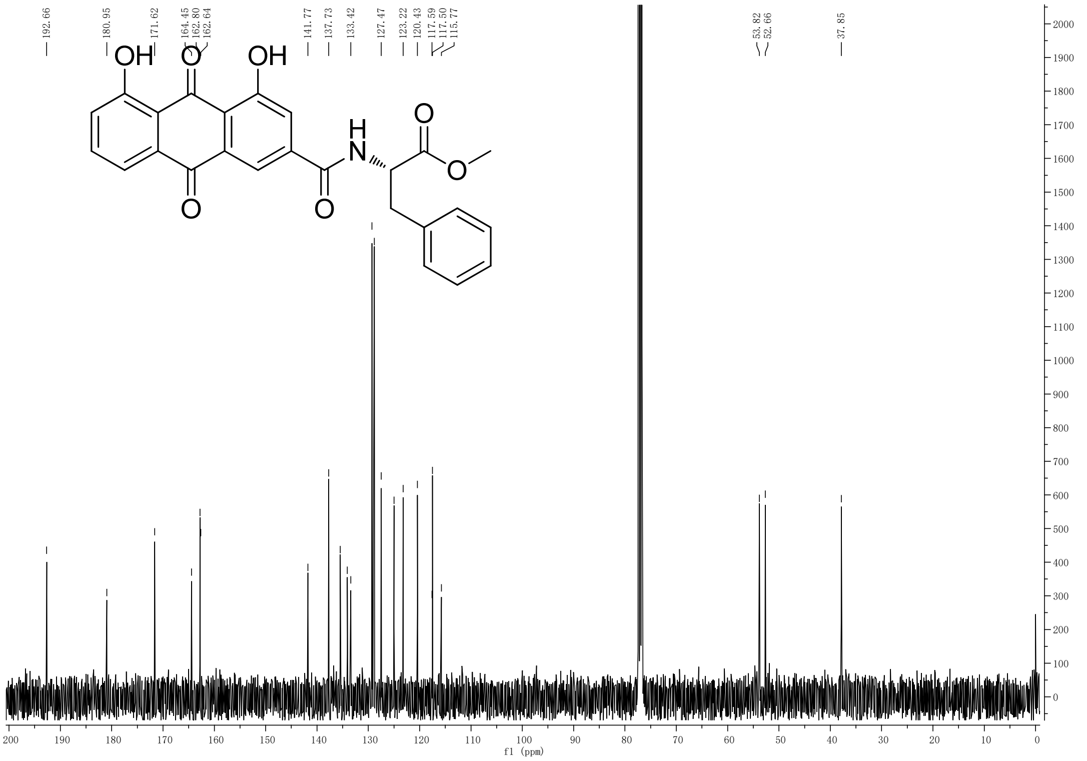

Figure S53. ^{13}C-NMR Spectrum of compound **3r**

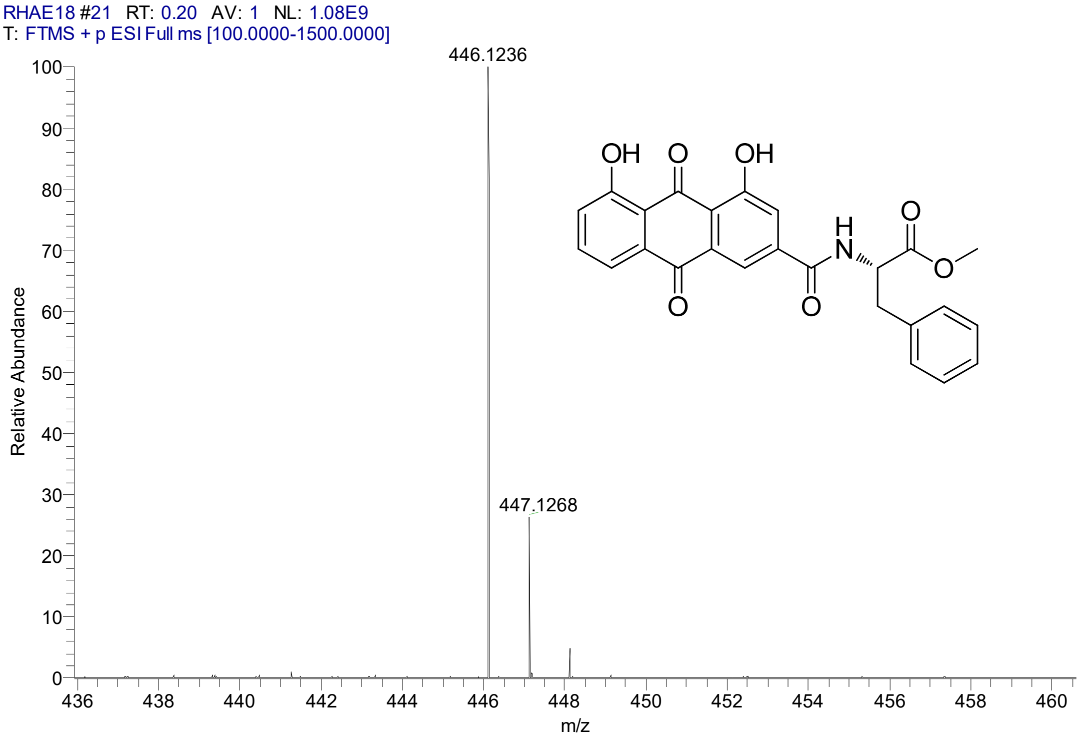

Figure S54. HRMS Spectrum of compound **3r**

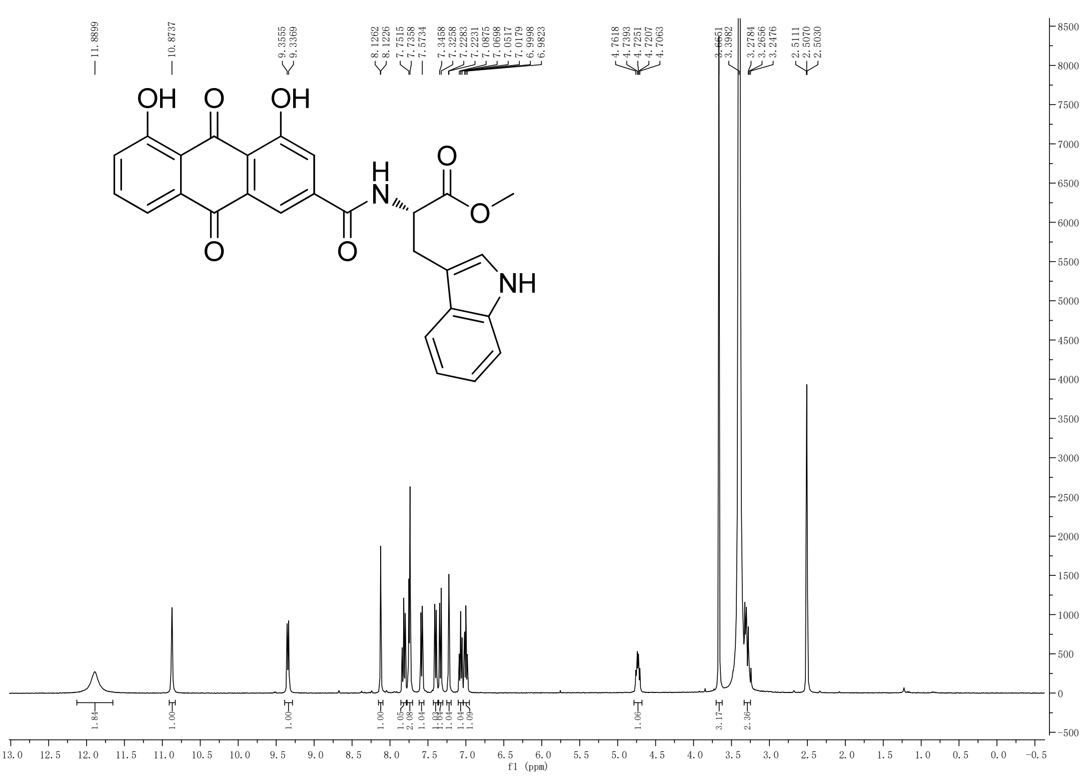

Figure S55. ^{1}H-NMR Spectrum of compound **3s**

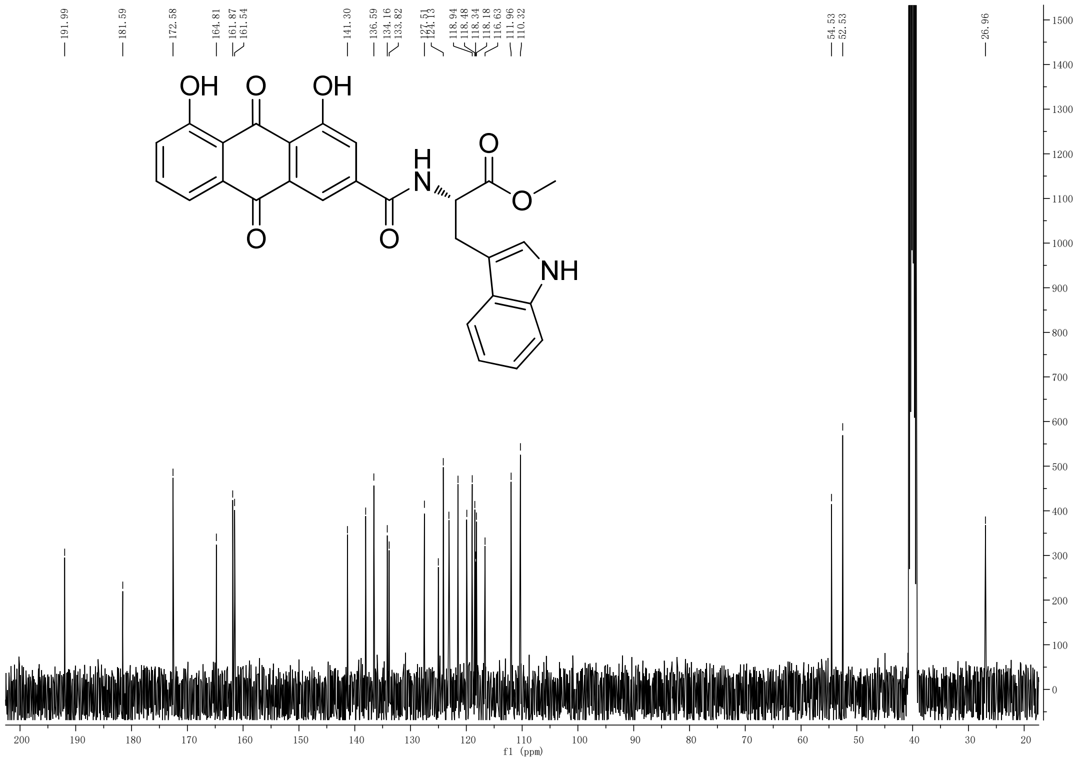

Figure S56. ^{13}C-NMR Spectrum of compound **3s**

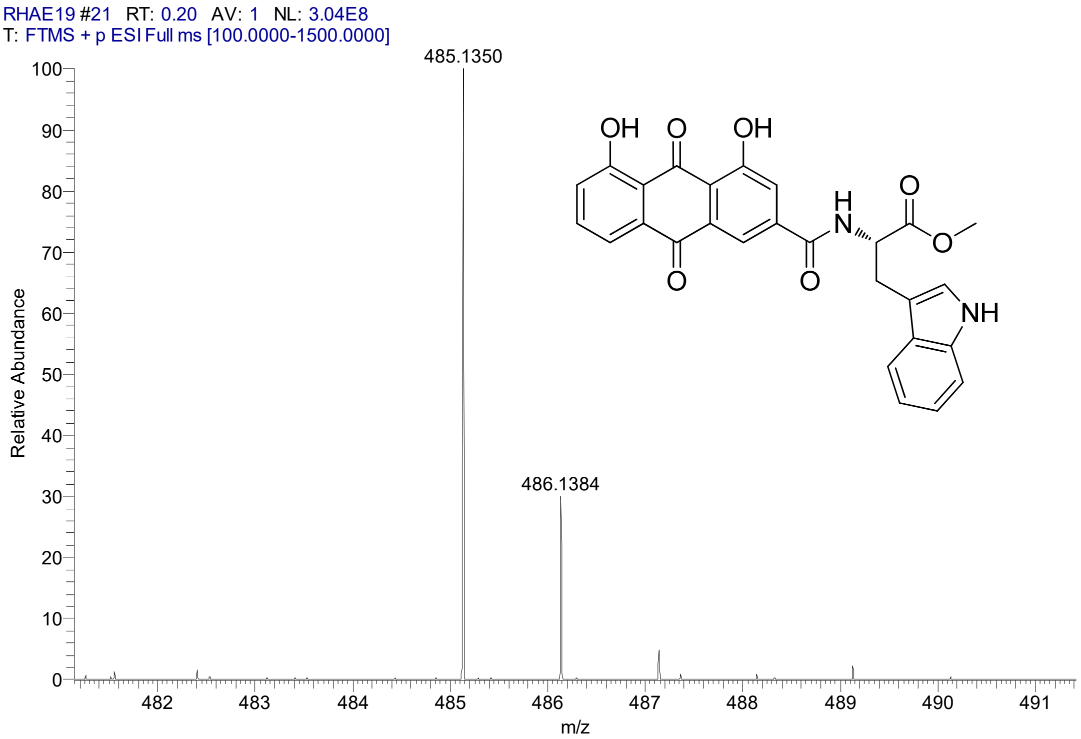

Figure S57. HRMS Spectrum of compound **3s**

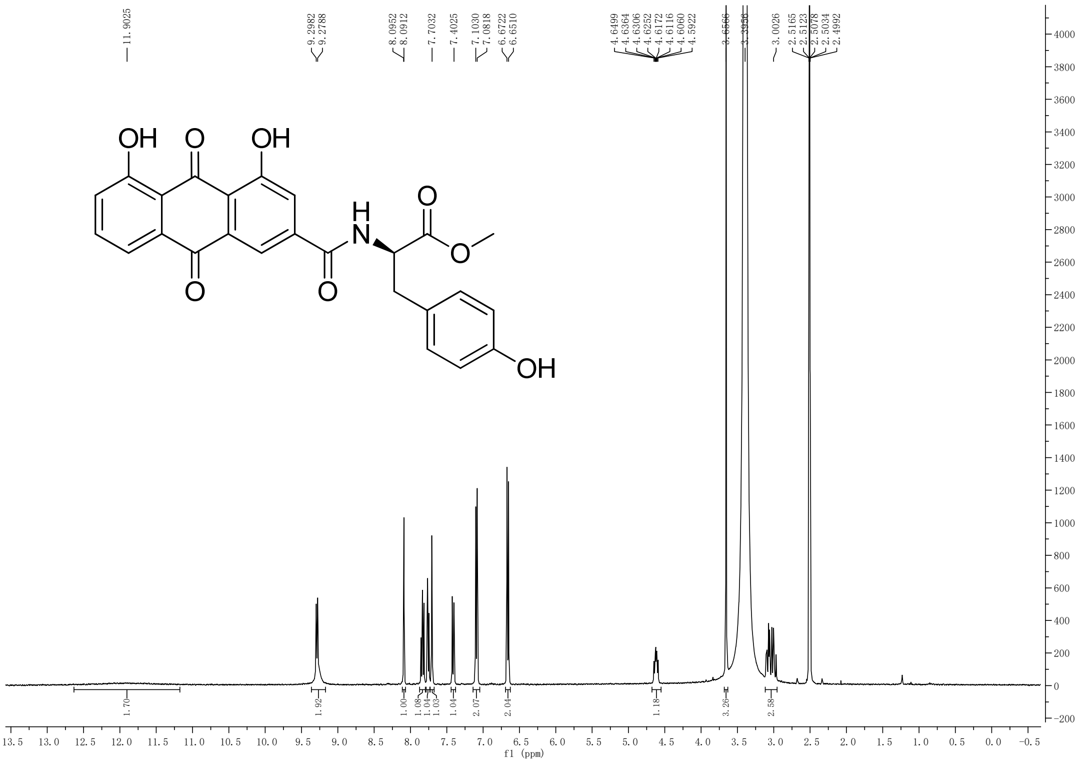

Figure S58. ^{1}H-NMR Spectrum of compound **3t**

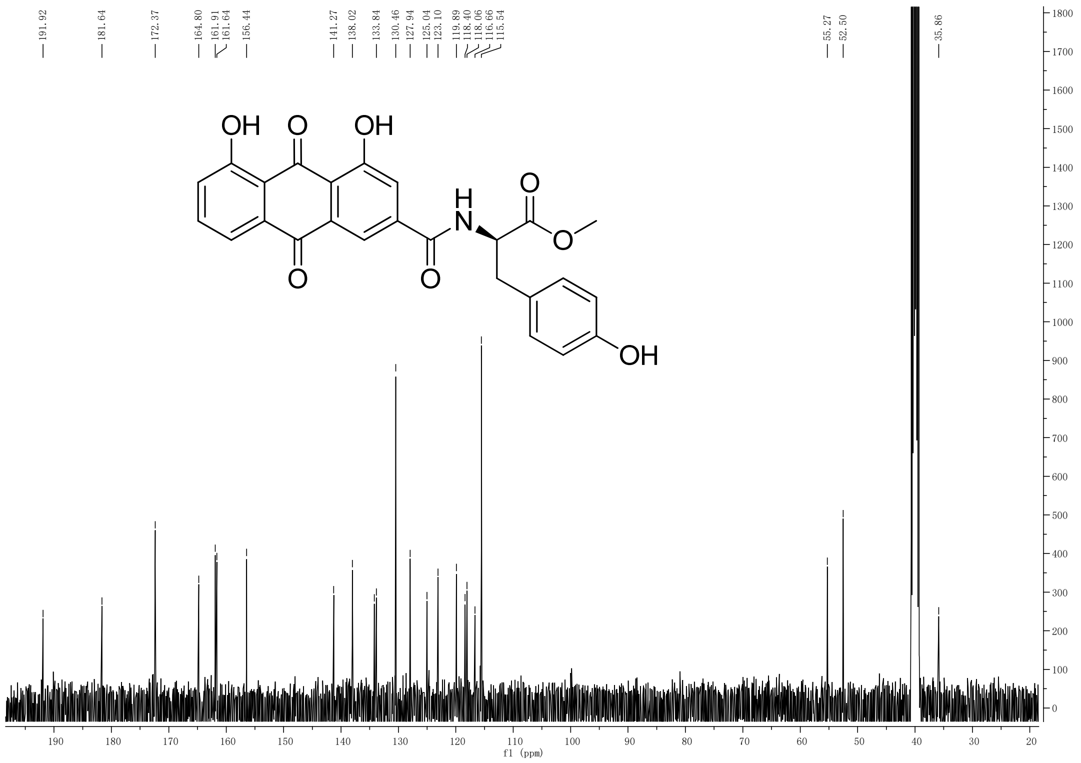

Figure S59. ^{13}C-NMR Spectrum of compound **3t**

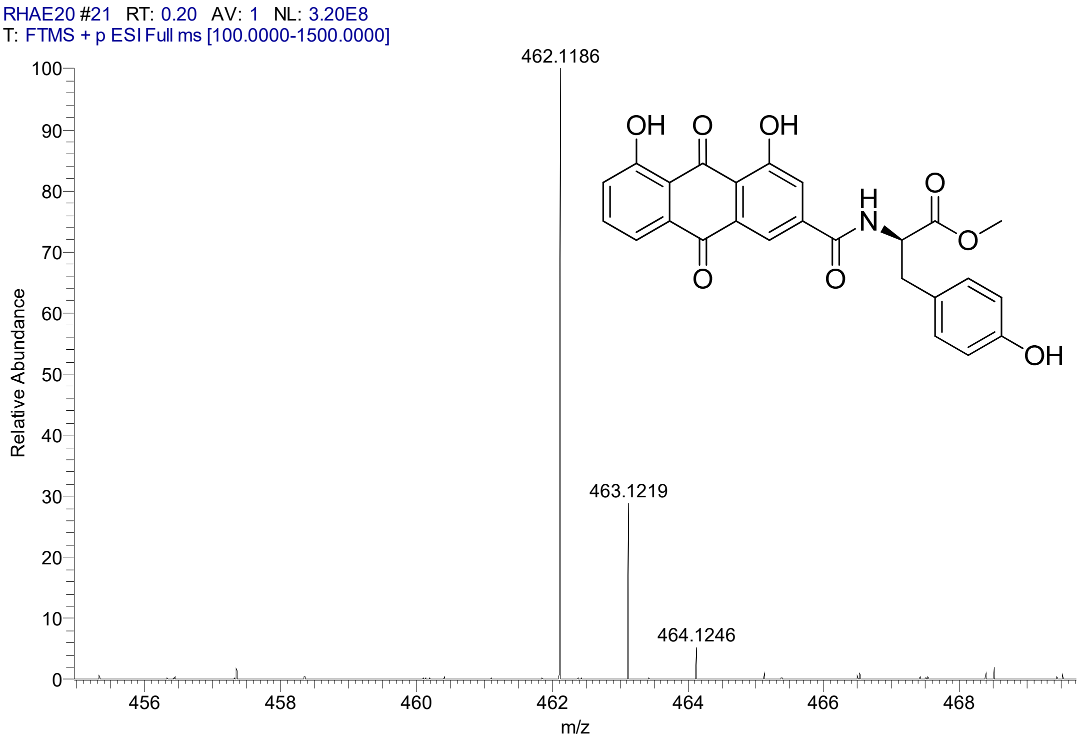

Figure S60. HRMS Spectrum of compound **3t**
